# Assessment of Changes in the Composition and Distribution of Large and Medium‐Sized Mammals in Xishuangbanna, Southwest China

**DOI:** 10.1002/ece3.70432

**Published:** 2024-11-03

**Authors:** Hui Cao, Rui‐Chang Quan, Yang Bai, Ruchuan He, Ying Geng, Ying Liu, Jiabin Li, Lin Wang

**Affiliations:** ^1^ Center for Integrative Conservation, Xishuangbanna Tropical Botanical Garden Chinese Academy of Sciences Mengla Yunnan China; ^2^ University of Chinese Academy of Sciences Beijing China; ^3^ Southeast Asia Biodiversity Research Institute Chinese Academy of Sciences Yezin Nay Pyi Taw Myanmar; ^4^ Yunnan International Joint Laboratory of Southeast Asia Biodiversity Conservation Menglun Yunnan China

**Keywords:** biodiversity, camera trap, inventory, monitoring, protected areas

## Abstract

Given the vulnerability of large and medium‐sized mammal communities to climate change and human disturbances, understanding the spatial–temporal dynamics of these communities is essential for effective conservation planning. However, in many biodiversity hotspots, precise biological community assessments are insufficient. From 2012 to 2022, we deployed 784 camera traps in eight nature reserves (including sub‐reserves) and one State Forest Farm (SFF, less strictly protected than a reserve) to study the composition and distribution of large and medium‐sized mammals in tropical Xishuangbanna. The findings revealed the following: (1) Forty‐three species, encompassing six orders, 17 families, and 37 genera, were documented. Among the species in historical data, nine species were not detected in this survey. (2) Smaller and more fragmented reserves lacked larger body‐sized predators and herbivores, and most common species showed lower relative population abundance. Conversely, the SFF exhibited high mammal diversity. (3) The community composition of large and medium‐sized mammals varied significantly across the nine sites, particularly among threatened species. Our findings highlight the uneven distribution of these mammal communities in Xishuangbanna, with rare and large‐sized species facing increased vulnerability to rapid environmental changes. Moreover, the findings demonstrate the importance of considering species specificity and uniqueness in conservation planning for maintaining regional‐scale biodiversity.

## Graphical abstract

We employed 785 camera traps to precisely assess large and medium‐sized mammal composition and distribution in tropical Xishuangbanna. Our findings highlight the uneven distribution of large and medium‐sized mammal communities in Xishuangbanna, with rare and large‐sized species being more vulnerable to rapid environmental changes. We emphasize that considering species specificity and uniqueness in conservation planning is key to maintaining biodiversity at the regional scale.

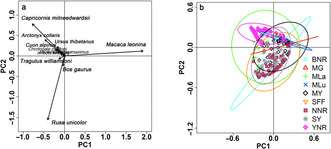

## 1 Introduction

The ongoing sixth mass extinction is occurring at a rate 1000 times higher than the background extinction rate (i.e., approximately 0.1 species per million species per year; Pimm et al. 2014), presenting an unprecedented crisis for global biodiversity. Unlike previous natural extinction events, human‐induced habitat modification has emerged as a significant driver of species extinction (Tollefson 2019). Over the past 500 years, nearly 1000 animal species have become extinct (Ceballos, García, and Ehrlich 2010). Without immediate and comprehensive restoration efforts, up to 40% of current species could face extinction in the coming decades (Pimm and Raven 2000). Protected areas (PAs) have demonstrated success in mitigating habitat degradation and providing refuge for numerous species (Geldmann et al. 2013). To combat biodiversity loss, the number of PAs worldwide increased from 217,995 to 285,525 between 2016 and 2022, now covering over 15.8% of the Earth's land area (UNEP‐WCMC 2022). Despite this growth, biodiversity monitoring within many PAs remains insufficient (Visconti et al. 2019), particularly in tropical regions (Beaudrot et al. 2016). Insufficient high‐quality data in tropical areas hinder the development of targeted conservation strategies and undermine conservation effectiveness, contributing to ongoing biodiversity decline in nearly half of the PAs (Laurance et al. 2012). Although selecting appropriate target communities and implementing long‐term monitoring present significant challenges, comprehensive studies assessing changes in species presence and community composition within PAs at Xishuangbanna are lacking.

Large and medium‐sized mammals play a crucial role in ecosystems (Lacher et al. 2019). Species such as elephants, primates, rodents, and ungulates are involved in dispersing seeds of at least 600 plant species in tropical regions (Fleming and Kress 2013). Carnivores regulate trophic level structure through predation (Williams et al. 2018; Brook, Johnson, and Ritchie 2012). However, these mammals face significant threats from hunting, deforestation, and climate change (Harrison et al. 2016; Alessa and Chapin 2008). From 1996 to 2022, the number of threatened mammal species has increased from 1096 to 1340, and the number of critically endangered species has risen from 169 to 233 (IUCN 2022). Large and medium‐sized mammals, characterized by low reproduction rates, long growth cycles, and limited resilience, are vulnerable to population declines and face a higher risk of future extinctions compared with other taxa, mainly owing to their larger home range requirements (Hedwig et al. 2018; Barlow et al. 2016; Cardillo et al. 2005). Consequently, large and medium‐sized mammals are valuable indicators of ecosystem health and are crucial for maintaining and restoring biodiversity.

Biodiversity has rapidly changed under the direct or indirect influence of land use and climate change (Chase et al. 2019), leading to alterations in community structure and ecological processes. However, owing to substantial differences in species' adaptability, species richness and population structure may require decades or even centuries to adapt to current environmental changes (Menéndez et al. 2006). Biodiversity changes, particularly in terms of species richness, are often scale‐dependent (Hillebrand et al. 2018). While global biodiversity loss is widely acknowledged, studies at regional scales have indicated that changes in various taxonomic groups are subtle and asynchronous. For example, butterfly species richness has notably increased in the United Kingdom over the past decade (Menéndez et al. 2006), while coral species richness in the tropical East Pacific has markedly declined during the same period (Gomez, Gonzalez, and Guzman 2018). Furthermore, the study across various taxa has revealed that the rate of biodiversity change at the regional level often surpasses that at the global level (Thomas et al. 2004). Therefore, investigating regional biodiversity changes is crucial given the rapid environmental transformations. Long‐term monitoring of specific taxa can enable the analysis of the causes and trends of species composition changes, leading to the development of targeted conservation management plans (Chase et al. 2019; Cardinale et al. 2018). Moreover, the contrasting species compositions found in heterogeneous habitats can inform the development of conservation networks and enhance conservation effectiveness (Rodrigues and Cazalis 2020; Xu et al. 2020).

Xishuangbanna lies within the Indo‐Burma biodiversity hotspot, harboring the largest tropical flora in China and supporting rich biodiversity. PAs encompass ~17.5% of Xishuangbanna's land area. However, the region faces challenges owing to the rapid expansion of rubber plantations, deforestation, hunting, and grazing (Yang, Xu, and Zhai 2021; Huang et al. 2020; Chang et al. 2019), leading to increased isolation and weakened connectivity among these PAs (Liu et al. 2020). Thirty years have passed since the last comprehensive scientific expedition in Xishuangbanna (Xu, Jiang, and Quan 1987), and the data from that time no longer align with current conservation efforts owing to changes in habitat environments. Furthermore, numerous studies have solely focused on rare and endangered species, with research on specific taxa still lacking. Therefore, there is an urgent need for a comprehensive study of the large and medium‐sized mammal communities and their changes in Xishuangbanna. In this study, we employed camera traps to collect distribution data on large and medium‐sized mammals in Xishuangbanna's tropical forest. Our objectives were as follows: (1) to establish a species inventory as baseline data for future conservation efforts; (2) to compare historical data to assess changes in species composition; and (3) to compare community composition, particularly focusing on threatened species, across different sites.

## 2 Materials and Methods

### 2.1 Study Area

The Xishuangbanna Dai autonomous prefecture is located in southwestern Yunnan Province, China, covering a total area of 19,322 km^2^. The elevation ranges from 390 to 2428 m and gradually decreases from west to east. The Lan Cang River, also known as the upper Mekong, flows through the prefecture and divides it into two parts. Xishuangbanna shares its border with Laos to the southeast and Myanmar to the west, totaling a border length of 966.3 km. The annual average precipitation is 1557 mm, with 13% occurring during the dry season and 87% during the rainy season. The annual average temperature is 21.4°C.

The Xishuangbanna region is home to four natural reserves: The Xishuangbanna National Nature Reserve (XNR, 2826.72 km^2^), Nabanhe Basin National Nature Reserve (NNR, 271.66 km^2^, hereafter referred to as the Nabanhe reserve), Bulong Prefectural Nature Reserve (BNR, 354.60 km^2^, hereafter referred to as the Bulong reserve), and Yiwu Prefectural Nature Reserve (YNR, 333.44 km^2^, hereafter referred to as the Yiwu reserve). XNR comprises five sub‐reserves: ManGao (MG, 94.41 km^2^), MengYang (MY, 1128.31 km^2^), MengLun (MLu, 119.04 km^2^), MengLa (MLa, 1151.79 km^2^), and ShangYong (SY, 333.17 km^2^). The elevation range and land use types of each reserve are illustrated in Figures S1–S16.

### 2.2 Camera‐Trap Survey

**FIGURE 1:**
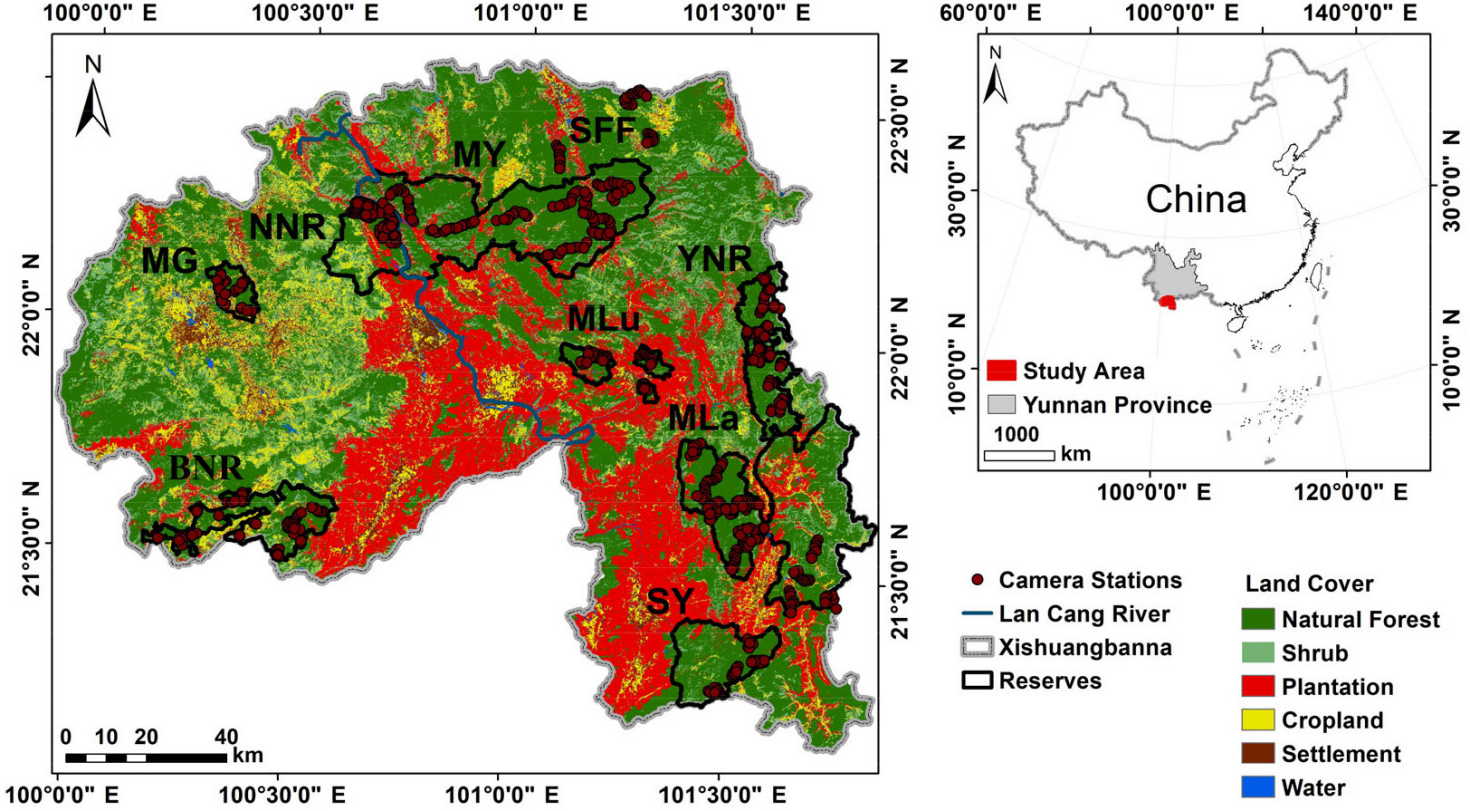
Camera stations (784) installed across all the nature reserves and a state forest farm (SFF) in Xishuangbanna. Abbreviations: BNR, Bulong Prefectural Nature Reserve; MG, ManGao sub‐reserve; MLa, MengLa sub‐reserve; MLu, MengLun sub‐reserve; MY, MengYang sub‐reserve; NNR, Nabanhe Basin National Nature Reserve; SY, ShangYong sub‐reserve; YNR, Yiwu Prefectural Nature Reserve.

From 2012 to 2022, 784 camera stations were deployed across the four reserves and one SFF (Figure 1). The distribution of the camera stations is detailed in Table 1. Factors such as vegetation types, elevation, and the intensity of human disturbance in each monitoring area were carefully considered in the deployment of the camera traps (Burton et al. 2015). The cameras were positioned at elevations ranging from 653 to 2325 m, mainly along abandoned human paths or animal trails. This placement ensured coverage of both the core and periphery of natural forest areas within the study site. To maintain sampling independence, adjacent camera stations were spaced at least 500 m apart during the same period.

**TABLE 1:** Deployment of camera traps in Xishuangbanna.

Sites	Sub‐reserves	Period	Camera‐trapping days	Camera stations	Independent events	Reserves size (km^2^)	No. of cameras per km^2^
XNR	MLu	2015.7–2021.4	31,973	50	4207	119.04	0.42
	MLa	2014.6–2014.8; 2015.6–2015.10; 2017.1–2020.8	51,270	164	7707	1151.79	0.14
	SY	2018.11–2022.11	17,752	36	5298	333.17	0.11
	MY	2019.3–2021.5	63,458	117	12,617	1128.31	0.11
	MG	2019.1–2020.3	5838	28	239	94.41	0.30
NNR	/	2012.6–2018.8	51,325	166	11,493	271.66	0.61
BNR	/	2014.3–2015.2; 2019.12–2020.11	18,617	86	1457	354.60	0.24
YNR	/	2017.7–2020.5	49,735	108	14,658	333.44	0.32
SFF	/	2019.11–2020.12	10,199	29	883	NA	NA
Total	/	2012.3–2022.11	300,167	784	58,559	3786.42	0.21

Abbreviations: /, not applicable; BNR, Bulong Prefectural Nature Reserve; MG, ManGao sub‐reserve; MLa, MengLa sub‐reserve; MLu, MengLun sub‐reserve; MY, MengYang sub‐reserve; NA, Not available; NNR, Nabanhe Basin National Nature Reserve; SFF, State Forest Farm. The total reserve size does not include SFF; SY, ShangYong sub‐reserve; XNR, Xishuangbanna National Nature Reserve; YNR, Yiwu Prefectural Nature Reserve.

The two camera models used were the Loreda L710‐940 (manufactured by Shenzhen Loreda Technology Co., Ltd., Shenzhen, China) and the Ltl Acorn Ltl‐6510 (manufactured by Zhuhai Ltl Acorn Electronics Co., Ltd., Zhuhai, China). These cameras were positioned in areas with relatively unobstructed views and signs of mammal activity. They were mounted to tree trunks at a height ranging from 0.5 to 1.5 m above the ground, with vegetation and weeds cleared from the camera's field of view. The cameras were configured to capture continuous footage 24 h a day, with three high‐definition shots taken in rapid succession, triggered with a 0‐s interval, and set to high sensitivity. No bait or lures were used in front of the cameras. Regular checks were conducted every 5–6 months to replace batteries and memory cards as needed.

### 2.3 Data Analysis

#### 2.3.1 Species Inventory

We classified mammals weighing > 2 kg as large and medium‐sized. The mammals encompassed species from Carnivora, Cetartiodactyla, Pholidota, Proboscidea, terrestrial Primates, and some large Rodentia, such as Sciuridae, Hystricidae, and Spalacidae. Indistinguishable species, species dependent on water (such as *Aonyx cinerea*, *Lutra lutra*, and *Lutrogale perspicillata*), and free‐grazing livestock were excluded from the species inventory. To avoid overestimating the detection rates of species, multiple photographs of the same species captured from the same camera stations within < 30 min were considered a single independent event (O'Brien 2008). Additionally, we collected species distribution data from the existing literature (hereafter referred to as “literature data”). Using keywords such as “Mammals,” “Xishuangbanna,” and “Animals,” we conducted searches for published papers on Web of Science, Google Scholar, and the China National Knowledge Infrastructure (CNKI, in Chinese) to compile a historical species list. This list was then compared with the current species list.

#### 2.3.2 Species Diversity

In this study, detection rates were utilized as a relative abundance index (RAI). The trap detection rates for each species were calculated as the number of independent photographs captured of a species (*N*_i_) per 100 trap days using the formula: RAI = *N*_i_/*D* × 100, where *D* represents the total number of camera‐trap days accumulated during the study (O'Brien 2008). To assess the sampling completeness of the large and medium‐sized mammal communities at each site, we employed the *SPECACCUM* function in the *VEGAN* package to conduct sparse curve analysis. Species richness for each site was estimated using four nonparametric species richness estimators: Chao1, Jacknife1, the incidence‐based coverage estimator, and the abundance‐based coverage estimator in EstimateS v.9.0 (Colwell 2013), which are widely employed for estimating species richness. We assessed the correlation between PA size and species richness using Spearman's rank correlation coefficient, with the *rcorr* function in the *HMISC* package. All of the aforementioned analyses were conducted in EstimateS v.9.0 and R v.4.1.0.

#### 2.3.3 Community Composition

Using the Bray–Curtis dissimilarity distance, we conducted PERMANOVA (permutational multivariate analysis of variance, ADONIS) with *p*‐value correction through the “Bonferroni” method using the *ADONIS* function in the *VEGAN* package to compare community composition among sites and between the east and west banks of the Lan Cang River. To ensure statistical robustness, we excluded species with limited detection (less than five occurrences). The relative abundance of threatened species (including those categorized as “critically endangered,” “endangered,” and “vulnerable”) (IUCN 2022) was compared among sites through principal component analysis (PCA) on the species‐site matrix with “Hellinger” transformed species abundance (implemented through the *RDA* function in the *VEGAN* package). Additionally, multiple comparisons on the composition of threatened species among sites were conducted using the *PAIRWISE.ADONIS* function in the *GRIDEXTRA* package. *Tragulus williamsoni* is considered a rare species in Xishuangbanna and has not been evaluated by the IUCN owing to data insufficiency; however, for the purpose of multiple comparisons, it was treated as a threatened species. All of the aforementioned analyses were performed in R v.4.1.0.

## 3 Results

### 3.1 List of the Large and Medium‐Sized Mammals in Xishuangbanna

**FIGURE 2:**
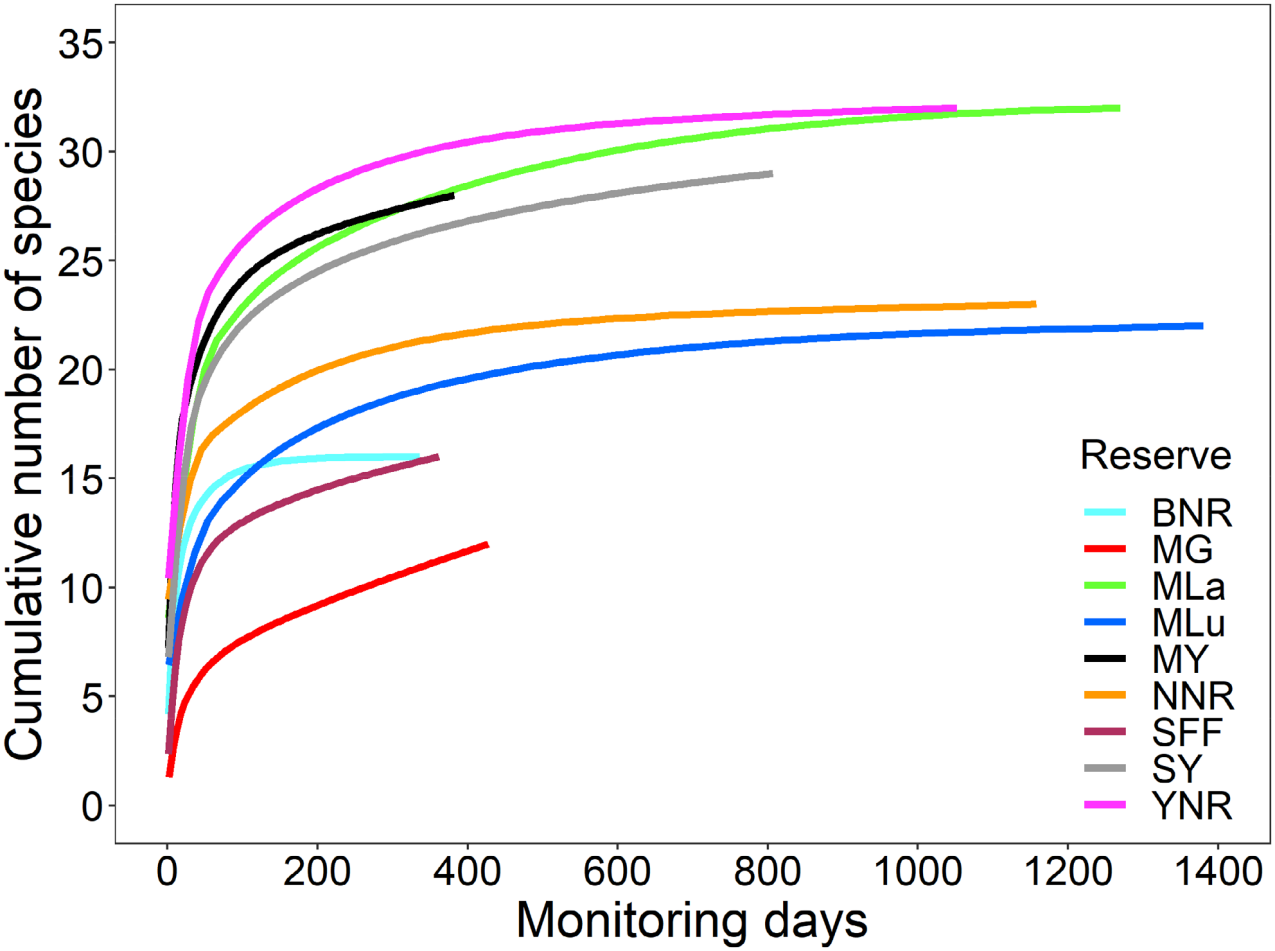
Species accumulation curves illustrating sampling efforts based on camera‐trap surveys conducted at nine sites in Xishuangbanna. Abbreviations: BNR, Bulong Prefectural Nature Reserve; MG, ManGao sub‐reserve; MLa, MengLa sub‐reserve; MLu, MengLun sub‐reserve; MY, MengYang sub‐reserve; NNR, Nabanhe Basin National Nature Reserve; SFF, state forest farm; SY, ShangYong sub‐reserve; YNR, Yiwu Prefectural Nature Reserve.

A total of 58,559 independent events of large and medium‐sized mammals were recorded over 300,167 camera‐trapping days (Table 1). Species accumulation curves indicated that the sampling effort was sufficient to reflect the composition of large and medium‐sized mammals (Figure 2). According to data from the camera‐trap surveys and information from 88 literature sources (see Data S1), a historical record of 52 large and medium‐sized mammals from seven orders, 19 families, and 43 genera was established for Xishuangbanna (Table 2). The record included 19 species categorized as threatened according to the IUCN Red List. Camera‐trap surveys captured 43 large and medium‐sized mammals from six orders, 17 families, and 37 genera, including one new distribution record (*Helarctos malayanus*). Specifically, species richness was observed as follows: 24 species in the MengLun sub‐reserve, 32 species in the MengLa sub‐reserve, 29 species in the ShangYong sub‐reserve, 28 species in the MengYang sub‐reserve, 12 species in the ManGao sub‐reserve, 23 species in the Nabanhe reserve, 16 species in the Bulong reserve, 32 species in the Yiwu reserve, and 16 species in the SFF (Tables S1–S9). Among the species in the literature data, nine were not detected in the camera‐trap survey, namely *Nomascus leucogenys*, *Canis lupus*, *Mustela sibirica*, *M. kathiah*, *Arctogalidia trivirgata*, *Felis chaus*, *Panthera corbetti*, *Elaphodus cephalophus*, and *Lepus comus*.

**TABLE 2:** List of recorded large and medium‐sized mammals in Xishuangbanna and their relative abundance index (RAI).

	Species	English name	IUCN red list	RAI									
				XNR					NNR	BNR	YNR	SFF	Total
				MLu	MLa	SY	MY	MG					
Primates													
Lorisidae													
1	*Nycticebus bengalensis*	Bengal Slow Loris	EN	—	—	—	—	—	—	—	—	—	□
Cercopithedidae													
2	*Macaca arctoides*	Stump‐tailed Macaque	VU	—	—	—	—	—	—	—	—	—	□
3	*Macaca assamensis*	Assamese Macaque	NT	0.009	0.084	0.015	0.050	—	0.008	—	0.016	0.010	0.033
4	*Macaca leonina*	Northern Pig‐tailed Macaque	VU	1.039	0.211	0.749	0.656	0.051	1.163	0.129	0.229	0.176	0.581
5	*Macaca mulatta*	Rhesus Monkey	LC	0.034	0.006	0.101	0.427	0.274	0.955	—	0.012	0.108	0.275
6	*Trachypithecus crepusculus*	Indochinese Gray Langur	EN	—	—	—	—	—	—	—	—	—	□
Hylobatidae													
7	*Nomascus leucogenys*	Northern White‐cheeked Gibbon	CR	—	—	—	—	—	—	—	—	—	▲
Pholidota													
Manidae													
8	*Manis pentadactyla*	Chinese Pangolin	CR	0.003	—	—	0.006	—	—	—	—	—	0.002
Carnivora													
Canidae													
9	*Canis lupus*	Gray Wolf	LC	—	—	—	—	—	—	—	—	—	▲
10	*Cuon alpinus*	Dhole	EN	0.013	0.012	0.023	0.022	—	0.008	—	0.281	—	0.057
11	*Vulpes vulpes*	Red Fox	LC	—	—	—	—	—	—	—	—	—	*
Ursidae													
12	*Helarctos malayanus*	Sun Bear	VU	—	—	0.006	—	—	—	—	0.002	—	0.001
13	*Ursus thibetanus*	Asiatic Black Bear	VU	0.013	0.066	0.163	0.071	—	0.014	0.156	0.189	0.029	0.082
Mustelidae													
14	*Arctonyx collaris*	Greater Hog Badger	VU	0.097	0.088	0.006	0.047	—	0.109	—	0.772	—	0.182
15	*Martes flavigula*	Yellow‐throated Marten	LC	0.063	0.246	0.253	0.295	0.223	0.018	0.172	0.569	0.314	0.249
16	*Melogale moschata*	Small‐toothed Ferret Badger	LC	1.035	0.870	0.524	0.227	0.017	0.084	0.478	0.306	0.137	0.437
17	*Mustela kathiah*	Yellow‐bellied Weasel	LC	—	—	—	—	—	—	—	—	—	▲
18	*Mustela sibirica*	Siberian Weasel	LC	—	—	—	—	—	—	—	—	—	▲
19	*Mustela strigidorsa*	Stripe‐backed Weasel	LC	0.009	0.011	0.011	0.002	—	—	0.048	0.020	0.010	0.010
Viverridae													
20	*Arctictis binturong*	Binturong	VU	0.031	0.014	0.101	0.006	—	—	—	0.010	—	0.015
21	*Arctogalidia trivirgata*	Small‐toothed Palm Civet	LC	—	—	—	—	—	—	—	—	—	▲
22	*Chrotogale owstoni*	Owston's Civet	EN	—	0.037	—	—	—	—	—	0.099	—	0.023
23	*Paguma larvata*	Masked Palm Civet	LC	0.744	1.006	0.631	1.124	1.799	0.487	1.380	1.468	0.814	1.001
24	*Paradoxurus hermaphroditus*	Common Palm Civet	LC	0.873	0.486	0.901	0.304	—	0.008	0.537	0.022	0.157	0.337
25	*Prionodon pardicolor*	Spotted Linsang	LC	0.009	0.051	0.039	0.016	—	0.008	0.430	0.050	0.010	0.030
26	*Viverra megaspila*	Large‐spotted Civet	EN	0.003	—	—	—	—	—	—	—	—	0.001
27	*Viverra zibetha*	Large Indian Civet	LC	—	0.002	0.006	—	0.017	—	—	0.099	—	0.017
28	*Viverricula indica*	Small Indian Civet	LC	0.003	0.004	—	0.047	0.017	—	—	0.125	—	0.032
Herpestidae													
29	*Herpestes javanicus*	Javan Mongoose	LC	—	—	—	—	—	—	—	—	—	□
30	*Herpestes urva*	Crab‐eating Mongoose	LC	0.394	0.217	0.744	0.247	—	0.152	0.016	0.402	0.147	0.274
Felidae													
31	*Catopuma temminckii*	Asiatic Golden Cat	NT	—	0.039	0.028	—	—	0.002	—	0.119	—	0.028
32	*Felis chaus*	Jungle Cat	LC	—	—	—	—	—	—	—	—	—	▲
33	*Neofelis nebulosa*	Clouded Leopard	VU	—	0.004	0.023	0.009	—	—	—	0.074	—	0.016
34	*Panthera pardus*	Leopard	VU	—	—	—	—	—	—	—	0.008	—	0.001
35	*Panthera corbetti*	Indochinese Tiger		—	—	—	—	—	—	—	—	—	▲
36	*Pardofelis marmorata*	Marbled Cat	NT	—	0.004	—	—	—	—	—	0.040	—	0.007
37	*Prionailurus bengalensis*	Mainland Leopard Cat	LC	0.529	0.298	0.282	0.550	0.480	0.359	0.231	0.754	0.147	0.455
Proboscidea													
Elephantidae													
38	*Elephas maximus*	Asian Elephant	EN	—	0.006	0.130	0.057	—	—	—	0.046	—	0.028
Cetartiodactyla													
Suidae													
39	*Sus scrofa*	Wild Boar	LC	3.099	2.908	2.248	2.097	1.045	4.099	1.681	5.501	1.618	3.195
Tragulidae													
40	*Tragulus williamsoni*	Williamson's Chevrotain	DD	—	0.156	4.495	—	—	—	—	0.090	—	0.307
Cervidae													
41	*Elaphodus cephalophus*	Tufted Deer	NT	—	—	—	—	—	—	—	—	—	▲
42	*Muntiacus vaginalis*	Northern Red Muntjac	LC	4.347	6.479	15.119	12.084	0.171	7.630	2.525	14.827	4.697	9.099
43	*Rusa unicolor*	Sambar	VU	—	0.269	2.191	0.295	—	4.789	0.097	—	—	1.062
Bovidae													
44	*Bos gaurus*	Gaur	VU	—	—	—	0.002	0.017	1.040	—	—	—	0.179
45	*Capricornis milneedwardsii*	Mainland Serow	VU	0.031	0.242	0.107	0.088	—	0.183	0.048	0.742	—	0.227
46	*Naemorhedus evansi*	Burmese Goral		—	—	—	—	—	0.425	—	—	—	0.072
Rodentia													
Sciuridae													
47	*Ratufa bicolor*	Black Giant Squirrel	NT	—	0.012	0.299	0.052	—	0.018	—	0.004	0.029	0.035
Spalacidae													
48	*Rhizomys pruinosus*	Bamboo Rat	LC	—	0.008	—	—	—	—	—	—	—	0.001
49	*Rhizomys sumatrensis*	Indomalayan Bamboo Rat	LC	0.031	0.043	0.011	0.016	—	—	—	0.010	—	0.016
Hystricidae													
50	*Atherurus macrourus*	Asiatic Brush‐tailed Porcupine	LC	0.641	1.003	0.361	0.545	—	0.425	0.252	0.649	—	0.572
51	*Hystrix brachyura*	Malayan Porcupine	LC	0.113	0.160	0.242	0.542	0.017	0.401	0.032	1.940	0.255	0.569
Lagomorpha													
Leporidae													
52	*Lepus comus*	Yunnan Hare	LC	—	—	—	—	—	—	—	—	—	▲
	Total number of species (From present survey)			24	32	29	28	12	23	16	32	16	43

*Note:* – Species not recorded by camera trap in the sites; *species recorded by Huang et al. (2020) with photo evidence; **□** species that were recorded by our team with photos but outside the nine survey sites; ▲ species that were recorded in the earlier literature data but not detected by this camera‐trap survey.

Abbreviations: BNR, Bulong Prefectural Nature Reserve; CR, critically endangered; DD, data deficient; EN, endangered; IUCN red list: LC, least concern; MG, ManGao sub‐reserve; MLa, MengLa sub‐reserve; MLu, MengLun sub‐reserve; MY, MengYang sub‐reserve; NNR, Nabanhe Basin National Nature Reserve; NT, near threatened; SFF, State Forest Farm; SY, ShangYong sub‐reserve; VU, vulnerable; XNR, Xishuangbanna National Nature Reserve; YNR, Yiwu Prefectural Nature Reserve.

### 3.2 Species Diversity and Distribution

The following common species were recorded across all nine sites in Xishuangbanna, representing various orders: *Macaca leonine* (Primates); *Martes flavigula*, *Melogale moschata*, *Paguma larvata*, and *Prionailurus bengalensis* (Carnivora); *Sus scrofa* and *Muntiacus vaginalis* (Artiodactyla); and *Hystrix brachyura* (Rodentia). Among them, *M. vaginalis* and *S. scrofa* were the most prevalent species in Xishuangbanna with RAI values of 9.099 and 3.195, respectively, collectively accounting for 61.42% of the occurrence frequency of large and medium‐sized mammals. However, certain species displayed restricted distributions. For example, *Helarctos malayanus* was solely recorded in the ShangYong sub‐reserve and the Yiwu reserve; *Panthera pardus* was exclusively recorded in the Yiwu reserve; and *Manis pentadactyla* was only observed in the MengLun sub‐reserve and the MengYang sub‐reserve. *T. williamsoni* was found in the MengLa sub‐reserve, ShangYong sub‐reserve, and Yiwu reserve (Table 2).

The results of the estimated species richness showed that the estimated values from Chao1 were the most similar to the observed species richness (Table 3). Overall, species richness showed a positive correlation with the size of the PAs (*r* = 0.61; *p* = 0.11; Figure S17). The highest species richness was estimated in the MengLa sub‐reserve ($\hat{S}$ = 32) and the Yiwu reserve ($\hat{S}$ = 32), followed by the MengYang sub‐reserve ($\hat{S}$ = 30). In contrast, the ManGao sub‐reserve, situated as a small fragment on the western side of Xishuangbanna, exhibited the lowest richness ($\hat{S}$ = 14).

**TABLE 3:** Observed and estimated species richness.

Sites		Species richness				
		Observed	Jacknife 1	Chao 1	ACE	ICE
XNR	MLu	24	22.98	22.00	22.47	22.32
	MLa	32	33.99	32.00	32.34	33.09
	SY	29	32.89	29.09	31.23	31.46
	MY	28	30.97	29.50	29.73	29.85
	MG	12	16.82	13.90	27.98	18.46
NNR	/	23	23.99	23.00	23.29	23.27
BNR	/	16	16.99	16.00	16.00	16.30
YNR	/	32	32.99	32.06	32.26	32.29
SFF	/	16	18.90	19.00	19.63	17.72

Abbreviations: /, not applicable; ACE, abundance‐based coverage estimator; BNR, Bulong Prefectural Nature Reserve; ICE, incidence‐based coverage estimator; MG, ManGao sub‐reserve; MLa, MengLa sub‐reserve; MLu, MengLun sub‐reserve; MY, MengYang sub‐reserve; NNR, Nabanhe Basin National Nature Reserve; SFF, state forest farm; SY: ShangYong sub‐reserve; XNR, Xishuangbanna National Nature Reserve; YNR, Yiwu Prefectural Nature Reserve.

### 3.3 Community Composition Differences Among Sites

The community composition differed significantly among sites (df = 8, *F* = 18.04, *p* = 0.001). Similarly, the community composition between the east and west banks of the Lan Cang River showed significant differences (df = 1, *F* = 31.99, *p* = 0.001), highlighting the pronounced barrier effect of the Lan Cang River on species distribution. The analysis of community composition for 12 threatened and rare species revealed significant differences among different sites (df = 8, *F* = 15.80, *p* = 0.001), as well as between the east and west banks of the Lan Cang River (df = 1, *F* = 23.40, *p* = 0.001). Pairwise ADONIS analysis revealed no significant difference in the community composition of threatened and rare species between the ManGao sub‐reserve, SFF, and the majority of other sites (Table 4). This suggests that small, isolated reserves, and nonprotected areas with high habitat quality, also play a role in conserving threatened and rare species.

**TABLE 4:** Pairwise ADONIS analysis of threatened and rare tropical species for nine sites in Xishuangbanna.

	BNR	SFF	MG	MLa	MLu	MY	NNR	SY	YNR
BNR									
SFF	5.81*								
MG	3.54	1.33							
MLa	5.08*	5.49*	2.41						
MLu	14.86*	2.92	2.18	11.86*					
MY	13.17*	2.45	2.27	16.71*	4.00*				
NNR	21.43*	10.35*	4.67	21.17*	18.45*	21.48*			
SY	10.33*	9.93*	4.50*	6.55*	16.50*	17.26*	8.28*		
YNR	13.05*	10.49*	4.70*	10.96*	21.81*	40.23*	55.85*	20.94*	

*Note:* The numbers shown in the table represent the statistical *F*, and *represents a *p*‐value less than 0.05.

Abbreviations: BNR, Bulong Prefectural Nature Reserve; MG, ManGao sub‐reserve; MLa, MengLa sub‐reserve; MLu, MengLun sub‐reserve; MY, MengYang sub‐reserve; NNR, Nabanhe Basin National Nature Reserve; SFF, state forest farm; SY, ShangYong sub‐reserve; YNR, Yiwu Prefectural Nature Reserve.

**FIGURE 3:**
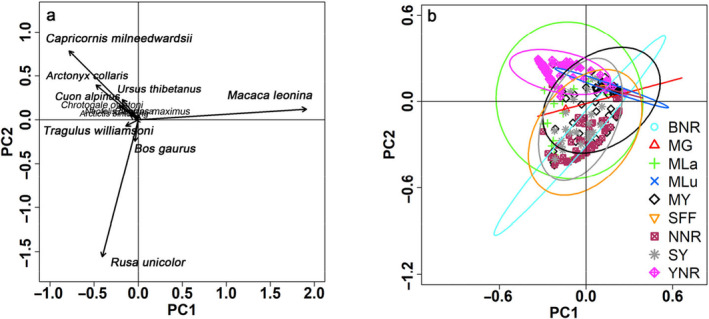
PCA outcomes for large and medium‐sized threatened and rare mammal composition, according to detection rates from camera traps across nine sites in Xishuangbanna. Panel (a) Species‐specific results, with arrows indicating the direction of maximum relative abundance change across the ordination plot (the arrow length represents the magnitude of change). Panel (b) 95% confidence interval fitting curve illustrating the variation in large and medium‐sized threatened and rare mammal composition among sites. Abbreviations: BNR, Bulong Prefectural Nature Reserve; MG, ManGao sub‐reserve; MLa, MengLa sub‐reserve; MLu, MengLun sub‐reserve; MY, MengYang sub‐reserve; NNR, Nabanhe Basin National Nature Reserve; SFF, state forest farm; SY, ShangYong sub‐reserve; YNR, Yiwu Prefectural Nature Reserve.

In PCA, the first two axes of the ordination diagram explained 54.79*%* of the total variation in the community composition of threatened and rare species among sites. The first axis accounted for 32.15% of this variation, while the second axis contributed 22.64%. The relative abundance of species was categorized into three groups (Figure 3). The first group included only one Primates species: *M. leonine*. The relative abundance of *M. leonina* increased along the first axis and was the highest in the larger and continuous tropical mountain evergreen broad‐leaved forest (i.e., ShangYong sub‐reserve, MengYang sub‐reserve, and Nabanhe reserve). The second group comprised herbivores *T. williamsoni*, *Bos gaurus*, and *Rusa unicolor*, which were not detected in the central region of Xishuangbanna (MengLun sub‐reserve). Particularly, the first two species exhibited a limited range and were rare. *T. williamsoni* was monitored only in the southeast and south of Xishuangbanna, while *Bos gaurus* was recorded solely in the north of Xishuangbanna. The third group included predominantly carnivore species, concentrated notably in the Yiwu reserve and the MengLa sub‐reserve. The greater variation in elevation in these areas creates a more complex and diverse habitat environment capable of supporting the coexistence of more carnivore species, particularly Felidae (Table 2). These species also encounter increased hunting pressure and are at risk of extinction.

## 4 Discussion

We assessed the large and medium‐sized mammal community in Xishuangbanna using extensive camera‐trap data. For species not recorded in this extensive survey, their absence possibly indicated either local extinction or a severe reduction in their population and distribution range. At the spatial scale, species distribution was found to be uneven, with large bodied species mainly distributed in larger forest patches. This study highlights the importance of periodically assessing the composition of large and medium‐sized mammals, as they serve as important indicators for evaluating the effectiveness of biodiversity conservation efforts.

### 4.1 Change in Species Composition

The historical status of *B. javanicus* in Xishuangbanna remains uncertain, and recent findings by Jiang (2021) confirm its regional extinction in China. Consequently, this species has been removed from the list of large and medium‐sized mammals in Xishuangbanna. Over the past 30 years, the natural forest in Xishuangbanna has undergone extensive human modification, with the rapid expansion of rubber plantations emerging as the primary driver of deforestation in the region (Yang, Xu, and Zhai 2021). Given the increased fragmentation and isolation among forest patches, some species may be confined to specific patches that were not covered by our field survey. Hence, the absence of species such as *C. lupus*, *F. chaus*, *E. cephalophus*, *A. trivirgata*, *L. comus*, *M. kathiah*, and *M. sibirica* in our current survey does not necessarily imply their complete disappearance from Xishuangbanna.

Certain species, such as *Vulpes vulpes*, with very low population densities, might evade detection in camera‐trap surveys. Although Huang et al. (2020) recorded *V. vulpes* in the MengLun sub‐reserve, our survey could not detect it, even though it covered the same area. However, our survey recorded newly discovered rare species such as *Chrotogale owstoni*. This finding suggests a significant expansion in the distribution range of this species compared with previous surveys (Tongkok et al. 2019). *Naemorhedus evansi* was previously considered a subspecies of *N. griseus* until Li et al. (2020) recognized *N. evansi* as a distinct species. In our survey, *N. evansi* was only detected in the Nabanhe reserve. Additionally, our investigation provided the first accurate distribution data for *H. malayanus* which was found in the ShangYong sub‐reserve and the Yiwu reserve. According to the 2022 “Assessment Report on the Endangered Status of Primates in China” by Li (2022), *N. leucogenys* has been declared extinct in the wild, and this species was also not detected during our survey. Furthermore, hunting remains a persistent issue in Xishuangbanna. During the survey, we frequently observed the presence of armed hunters (Figure S18), and in March 2024, we rescued a *P. larvata* injured by a trap near the MengLun sub‐reserve (Figure S19). Moreover, hunting has contributed to the disappearance of *P. corbetti* in Xishuangbanna. Since as early as 1984, the population of *P. corbetti* began to decline significantly, showing signs of extinction (Ji, Zhang, and Yang 1984). In March 2009, a village resident killed *P. corbetti* in the ShangYong sub‐reserve, and there have been no further sightings since (Feng et al. 2013). Currently, the status of this species remains unknown, and it may have disappeared from Xishuangbanna.

### 4.2 Uneven Distribution of Species

The correlation observed between the size of PAs and species richness indicates that larger reserves tend to harbor a greater diversity of species than smaller reserves. Larger reserves can encompass diverse habitats, providing shelter for a wider range of species. They also preserve extensive tropical mountain evergreen broad‐leaved forests, mainly found at elevations above 1200 m, making them suitable habitats for species such as *M. leonine* (Sun et al. 2020; Yang et al. 2019; Zhu et al. 2015). Smaller reserves such as ManGao and Bulong feature lower species richness and lower relative abundance of common species such as *M. vaginalis*, *S. scrofa*, *A. macrourus*, and *H. brachyura*. In contrast, larger reserves host a higher abundance of common species and serve as exclusive habitats for rare species. However, the positive correlation between PA size and species richness lacks statistical significance, possibly due to the limited sample size (eight reserves). Nonetheless, this indicates that species richness is not solely dictated by the size of PAs. Factors such as elevation, forest connectivity, topographical barriers, and land use changes also play pivotal roles (Snider et al. 2024; Freeman, Roehrdanz, and Peterson 2019; Badgley et al. 2017; Newbold, Hudson, and Hill 2015). Specifically, the Yiwu reserve ranges in elevation from 600 to 2030 m. The extensive elevation gradient and complex mountain environment offer diverse habitats, facilitating the coexistence of five Felidae species and contributing to the highest relative species abundance of other carnivores, such as *Cuon alpinus* and *Ursus thibetanus*, in this reserve.

Nangong Mountain in the MengLa sub‐reserve serves as a natural barrier to the northward distribution of certain species, such as *T. williamsoni* and *C. owstoni*, which were only detected south of the mountain. Similarly, the Lan Cang River acts as a barrier to species movement, resulting in significant differences in community composition between the east and west banks. For example, *N. evansi* was exclusively recorded on the west bank. Human disturbances further impede species movement beyond disturbed PAs. Over the past decades, land use changes have significantly accelerated in western Xishuangbanna compared with other regions (Xu et al. 2023). These reserves are predominantly surrounded by rubber plantations, croplands, and buildings, causing severe fragmentation and restricted connectivity. This results in significantly lower species diversity compared with other areas in the southeast.

The distribution of rare species in Xishuangbanna aligns closely with patterns of species richness. Rare species, such as *P. marmorata*, *H. malayanus*, *T. williamsoni*, and *C. owstoni*, were predominantly found in sites with high species richness. This contrasts with global‐scale patterns where species richness is often influenced by widespread species (Loiseau et al. 2020). Rare species play a crucial role in maintaining community stability and fulfilling unique ecological functions that cannot be substituted by common species (Mouillot et al. 2013). In Xishuangbanna, rare species significantly contribute to overall species richness, representing a substantial proportion of threatened species. These species typically have restricted ranges, with recorded elevations averaging from above 1200 to 1700 m. This highlights the vulnerability of threatened species in Xishuangbanna to the impacts of global climate change and increasing human disturbances.

For over two decades, Xishuangbanna has grappled with significant human–wildlife conflict, resulting in a gradual decline in the population density of certain large and medium‐sized mammals such as Dhole, Sambar, Serow, Gaur, and Pangolin. Additionally, despite an estimated population of nearly 300 *E. maximus* in Xishuangbanna (Wang et al. 2017), the detection rate of *E. maximus* is relatively low (RAI = 0.028; Table 2), mainly owing to the expansion in the distribution of the species beyond reserve boundaries (Li et al. 2023). Globally, ~20% of threatened species are not protected within the PAs, mainly because of their limited ranges extending beyond the reserve boundaries (Venter et al. 2014; Rodrigues et al. 2004). In our study, two threatened species, *Macaca leonine* and *Ursus thibetanus*, were frequently observed outside the reserves. These findings highlight the importance of optimizing and maintaining reserves according to species‐specific requirements, natural distribution patterns, and habitat connectivity to effectively enhance biodiversity protection.

### 4.3 Conservation Suggestions

For communities marked by a high proportion of rare and threatened species, it is crucial to evaluate the risk factors and geographical distribution of these species at regional scales to enhance conservation effectiveness. The composition of threatened species in Xishuangbanna exhibited significant variation, underscoring the irreplaceable and complementary roles played by each site. Encroachment, combined with disturbance, only hastens species extinction. Furthermore, recognizing that reserves cannot operate as isolated islands within the natural landscape is imperative, given that large and medium‐sized mammals can migrate over extensive distances. Non‐networked reserves fail to provide an optimal habitat for biodiversity conservation.

We propose the following recommendations: (1) Landscape surrounding reserves, which provide shelter and buffers and facilitate species dispersal, are important features. Accurately mapping the distribution ranges of species, especially rare and threatened species, is crucial to ensure sufficient reserve coverage. (2) Large and medium‐sized mammals exhibit strong migration abilities. As habitats become increasingly isolated, there is a greater risk of populations becoming isolated. Establishing effective buffer zones, implementing robust protection networks, and maintaining connectivity are essential strategies to prevent reserves from becoming isolated from their surrounding environments. (3) Reducing deforestation, the conversion rate of forest cover types, and hunting activities are crucial measures. Given Xishuangbanna's strategic location at the convergence of three countries, it presents an excellent opportunity for regional integrated conservation efforts (Wang et al. 2021) and fosters international cooperation in governance and coordination to mitigate the adverse impacts of human disturbances. (4) Enhancing environmental education among local communities is vital. Empowering local communities with knowledge about environmental protection can contribute to sustainable development. This involves raising awareness about the importance of reducing hunting activities and instilling a greater sense of responsibility for biodiversity conservation and environmental protection.

## Author Contributions

**Hui Cao:** conceptualization (equal), data curation (equal), formal analysis (lead), writing – original draft (lead). **Rui‐Chang Quan:** conceptualization (lead), funding acquisition (lead), investigation (equal), methodology (equal), supervision (lead), writing – review and editing (lead). **Yang Bai:** conceptualization (equal), supervision (equal), writing – review and editing (equal). **Ruchuan He:** data curation (equal), formal analysis (supporting). **Ying Geng:** data curation (equal). **Ying Liu:** data curation (equal). **Jiabin Li:** data curation (equal). **Lin Wang:** conceptualization (lead), supervision (lead), writing – review and editing (lead).

## Conflicts of Interest

The authors declare no conflicts of interest.

## Supporting information


Data S1.

## Data Availability

Data have been uploaded as Supporting Information for review and publication.
