# Supplementary material for: Assessment of Changes in the Composition and Distribution of Large and Medium‐Sized Mammals in Xishuangbanna, Southwest China
Source: Ecol Evol. 2024 Nov 3;14(11):e70432. doi: 10.1002/ece3.70432 (PMC11531880; doi:10.1002/ece3.70432)
Supplement: Supplementary file 1 — Data S1. [file ECE3-14-e70432-s001.docx]

**Supplementary material**

**Assessment of changes in the composition and distribution of large and medium-sized mammals in Xishuangbanna, Southwest China**

This study used the keywords “Mammals,” “Xishuangbanna,” and “Animals” to gather species distribution data from existing literature on Web of Science, Google Scholar and China National Knowledge Infrastructure (CNKI, in Chinese) (literature data as follows).

**References (Literature data)**

Bai DF, Chen Y, Li JS, Tao Q, Wang LF, Piao Y, Shi K (2018) Mammal diversity in Shangyong Nature Reserve, Xishuangbanna, Yunnan Province. Biodiv Sci 26: 75-78. <https://doi.org/10.17520/biods.2017223> (in Chinese)

Bai DF, Wan XR, Zhang L, Campos-Arceiz A, Wei FW, Zhang ZB (2022) The recent Asian elephant range expansion in Yunnan, China, is associated with climate change and enforced protection efforts in human-dominated landscapes. Front Ecol Evol 10: 879. <https://doi.org/10.3389/fevo.2022.889077>

Campos-Arceiz A, de la Torre JA, Wei K, Wu XO, Zhu Y, Zhao M, Chen S, Bai Y, Corlett RT, Chen F (2021) The return of the elephants: How two groups of dispersing elephants attracted the attention of billions and what can we learn from their behavior. Conserv Lett 14: e12836. <https://doi.org/10.1111/conl.12836>

Cao M, Zhou W, Bai B, Zhang Q, Wang B, Chen MY (2010) Habitat Use of Williamson’s Mouse-deer (*Tragulus williamsoni*) in Mengla Area, Southern Yunnan. Zoological Research 31: 303-309. <https://doi.org/10.3724/SP.J.1141.2010.03303> (in Chinese)

Chen BPL, Li F, Ban D (2020) Two sighting records of Stripe-backed Weasel *Mustela strigidorsa* in Yunnan Province, China. Small Carniv Conserv 58: e58001.

Chen C, Quan RC, Cao G, Yang H, Burton AC, Meitner M, Brodie JF (2018) Effects of law enforcement and community outreach on mammal diversity in a biodiversity hotspot. Conserv Biol 33: 612-622. <https://doi.org/10.1111/cobi.13232>

Chen S, Yi ZF, Campos-Arceiz A, Chen MY, Webb EL (2013) Developing a spatially-explicit, sustainable and risk-based insurance scheme to mitigate human-wildlife conflict. Biol Conserv 168: 31-39. <http://dx.doi.org/10.1016/j.biocon.2013.09.017>

Dao DQ, Tong W (1955) Several animals of Xishuangbanna. Bulletin of Biology 1955: 10-11. (in Chinese)

Deng ZY, Li YS, Huang GL, Wan ZL, Duan WR, Zhang HR (2022) Summary of conservation and management of *Elephas maximus* in Xishuangbanna. Forest Inventory and Planning 47: 68-71. <https://doi.org/10.3969/j.issn.1671-3168.2022.03.012> (in Chinese)

Ding CC, Hu YM, Li CW, Jiang ZG (2018) Distribution and habitat suitability assessment of the gaur *Bos gaurus* in China. Biodiv Sci 26: 951-961. <https://doi.org/10.17520/biods.2018012> (in Chinese)

Fan PF, Fei HL, Luo AD (2014) Ecological extinction of the northern white-cheeked gibbon (*Nomascus leucogenys*) in China. Oryx 48: 52-55. <https://doi.org/10.1017/S0030605312001305>

Feng LM, Sun SH, Mao C, Zheng Y (2011) Tropical birds and mammals in Xishuangbanna. Forest & Humankind 2011: 26-35. (in Chinese)

Feng LM, Wang LF, Wang B, Smith JLD, Zhang L (2013) Population status of the Indochinese tiger (*Panthera tigris cobetti*) and density of the three primary ungulate prey species in Shangyong Nature Reserve, Xishuangbanna, China. Acta Theriologica Sinica 33: 308-318. <https://doi.org/10.16829/j.slxb.2013.04.004> (in Chinese)

Feng LM, Zhang L (2005) Habitat selection by Asian Elephant (*Elephas maximus*) in Xishuangbanna Yunnan, China. Acta Theriologica Sinica 25: 229-236. <https://doi.org/10.16829/j.slxb.2005.03.004> (in Chinese)

Gan HX, Hu HB (2008) Biodiversity conservation corridor design based on habitat selection of gaur (*Bos gaurus*): A case study from Xishuangbanna, China. Chinese Journal of Ecology, 27: 2153-2158. <https://doi.org/10.13292/j.1000-4890.2008.0053> (in Chinese)

Gao YT, Deng BG (1959) Xishuangbanna mainly economic birds and mammals. Chinese Journal of Zoology 1959: 398-404. <https://doi.org/10.13859/j.cjz.1959.09.005> (in Chinese)

Gao YT, Lu CK, Zhang J, Wang S (1962) Investigation report of mammals in Xishuangbanna, Yunnan Province. Acta Zoologica Sinica 1962: 180-196. (in Chinese)

Guo W, Cao G, Quan RC (2017) Population dynamics and space use of wild boar in a tropical forest, Southwest China. Glob Ecol Conserv 11: 115-124. <https://doi.org/10.1016/j.gecco.2017.04.005>

Guo W, Zhang M, Zhou L, Quan RC (2017) The rediscovery of Large-spotted Civet *Viverra megaspila* in China. Small Carniv Conserv 55: 88-90.

Guo XM, He Q, Wang LX, Yang ZB, Li ZY, Zhu ZY (2012) Effect of Asian elephant food source base on the mitigation of human-elephant conflict in Xishuangbanna of Yunnan Province, Southwest China. Chinese Journal of Ecology 31: 3133-3137. <https://doi.org/10.13292/j.1000-4890.2012.0427> (in Chinese)

Guo YL, Zhang L, Dong YH (2006) Foraging behavior of Asian elephants in Xishuangbanna, Yunnan, China. Acta Theriologica Sinica 26: 54-58. <https://doi.org/10.16829/j.slxb.2006.01.010> (in Chinese)

He C, Du J, Zhu D, Zhang L (2020) Population viability analysis of small population: A case study for Asian elephant in China. Integr Zool 15: 350-362. <https://doi.org/10.1111/1749-4877.12432>

He R, Wang L, Quan RC (2020) Introduction to transboundary animal diversity monitoring platform of Southern Yunnan, China and Southeast Asia. Biodiv Sci 28: 1097-1103. <https://doi.org/10.17520/biods.2020154> (in Chinese)

He SQ (2008) Overview of wildlife resource monitoring in Xishuangbanna National Nature Reserve. Forest Inventory and Planning 33: 84-86. (in Chinese)

Hu JC (1995) The bear resource and its protection in Southwest China. Journal of Sichuan Teachers College (Natural Science) 4: 274-278. (in Chinese)

Hu JS, Jiang WG, Wu JL, Li ZQ, Li CH (1999) Status of Indochinese tigers in Xishuangbanna. Chinese Journal of Wildlife 1999: 8-19. <https://doi.org/10.19711/j.cnki.issn2310-1490.1999.02.005> (in Chinese)

Hu Y, Chen JW, Feng J (2016) White-cheeked gibbon community activities in Xishuangbanna. Forest & Humankind 2016: 55-57+54. (in Chinese)

Huang C, Li X, Hu W, Jiang X (2019) Predicting indirect effects of transportation network expansion on Asian elephants: Implications for environmental impact assessments. Biotropica 52: 196-202. <https://doi.org/10.1111/btp.12726>

Huang G, Sreekar R, Velho N, Corlett RT, Quan RC, Tomlinson KW (2020) Combining camera-trap surveys and hunter interviews to determine the status of mammals in protected rainforests and rubber plantations of Menglun, Xishuangbanna, SW China. Anim Conserv 23: 689-699. <https://doi.org/10.1111/acv.12588> (in Chinese)

Ji BQ, Zhang CJ, Yang DH (1984) Recent status and population estimation of Indochinese tigers in Xishuangbanna. Forest Inventory and Planning 1984: 31-33. (in Chinese)

Li G, Sun N, Swa K, Zhang M, Lwin YH (2020) Phylogenetic reassessment of gorals with new evidence from northern Myanmar reveals five distinct species. Mammal Rev 50: 325-330. <https://doi.org/10.1111/mam.12200>

Li LL, Wang QY, Yang HP, Tao YX, Wang LX, Yang ZB, Campos-Arceiz A, Quan RC, (2022) Mobile animals and immobile protected areas: improving the coverage of nature reserves for Asian elephant conservation in China. Oryx 57: 532-539. <https://10.1017/S0030605322000515>

Li W, Liu P, Guo X, Wang L, Wang Q, Yu Y, Dai Y, Li L, Zhang L (2018) Human-elephant conflict in Xishuangbanna Prefecture, China: Distribution, diffusion, and mitigation. Glob Ecol Conserv 16: e00462. <https://doi.org/10.1016/j.gecco.2018.e00462>

Li WW, Yu Y, Li L, Zhang L (2017) Risk assessment of Human-elephant conflict in Xishuangbanna, Yunnan Province. Abstracts of the 13th National Symposium on Wildlife Ecology and Resource Conservation and the sixth Symposium on Zoology in West China, 2017: 142. (in Chinese)

Li X, Hu W, Pu C, Li Q, Yu Q, Hu Z, Bleisch WV, Jiang X (2020) Camera-trapping monitoring platform for mammals and pheasants in the Longitudinal Range and Gorge Region of Southwest China: Protocol, progress and future outlook. Biodiv Sci 28: 1090-1096. <https://doi.org/10.17520/biods.2020105> (in Chinese)

Li ZL, Chen MY, Wu ZL, Wang Q, Dong YH (2009) Perception and attitude of rural community to the construction of Asian elephant conservation corridors in Xishuangbanna. Chinese Journal of Applied Ecology 20: 1483-1487. <https://doi.org/10.13287/j.1001-9332.2009.0223> (in Chinese)

Liang DJ (1989) Species and characteristics of wildlife in Xishuangbanna. Tropical Geography 1989: 257-264. <https://doi.org/10.13284/j.cnki.rddl.002008> (in Chinese)

Liu J, Wang Y, Zhang L, Cui N, Lv T, Huang Y, Dai R, Li Z, Chen M (2022) Potential effect on migration of Asian elephants and human-elephant conflict by the Meng’a reservoir in Menghai County, Xishuangbanna. Acta Theriologica Sinica 42: 227-239. <https://doi.org/10.16829/j.slxb.150578> (in Chinese)

Liu JL, Xu TY, Ze D (2020) Study on rationality of "Closed" protection of Nature Reserve-Reflections on the Asian elephant accident in Xishuangbanna. Issues of Forestry Economics 40: 1-7. <https://doi.org/10.16832/j.cnki.1005-9709.2020.01.001> (in Chinese)

Liu L, Guo X, Luo A, Zhang L (2016) The impact of Asian elephant browsing on five plant species at Wild Elephant Valley, Xishuangbannan, Yunnan of China. Acta Theriologica Sinica 36: 129-137. <https://doi.org/10.16829/j.slxb.201602001> (in Chinese)

Liu L, Jin YF, Yang HP, Luo AD, Guo XM, Wang LF, Zhang L (2015) Evaluation of habitat for Asian elephants (*Elephas maximus*) in Xishuangbanna, Yunnan, China. Acta Theriologica Sinica 35: 1-13. <https://doi.org/10.16829/j.slxb.2015.01.001> (in Chinese)

Liu L, Zhang L, Luo A, Wang L, Zhang L (2011) Population dynamics, structure and seasonal distribution pattern of Asian elephant (*Elephas maximus*) in Shangyong Protected Area, Yunnan, China. Acta Theriologica Sinica 31: 226-234. <https://doi.org/10.16829/j.slxb.2011.03.002> (in Chinese)

Liu L, Zhu WQ, Zhang LT, Feng LM, Wang LF, Tao Q, Zhang L (2008) The opening up and utilization of a new movement corridor by Asian elephants (*Elephas maximus*) in Shangyong Nature Reserve, Yunnan. Acta Theriologica Sinica 28: 325-332. <https://doi.org/10.16829/j.slxb.2008.04.001> (in Chinese)

Liu P, Wen H, Harich FK, He C, Wang L, Guo X, Zhao J, Luo A, Yang H, Sun X, Yu Y, Zheng S, Guo J, Li L, Zhang L (2017) Conflict between conservation and development: cash forest encroachment in Asian elephant distributions. Sci Rep 7: 6404. <https://doi.org/10.1038/s41598-017-06751-6>

Liu P, Wen H, Lin L, Liu J, Zhang L (2016) Habitat evaluation for Asian elephants (*Elephas maximus*) in Lincang: Conservation planning for an extremely small population of elephants in China. Biol Conserv 198: 113-121. <https://doi.org/10.1016/j.biocon.2016.04.005>

Liu S, Dong Y, Cheng F, Zhang Y, Hou X, Dong S, Coxixo A (2017) Effects of road network on Asian elephant habitat and connectivity between the nature reserves in Xishuangbanna, Southwest China. J Na Conserv. 38: 11-20. <http://dx.doi.org/10.1016/j.jnc.2017.05.001>

Luo AD, Huang JG, Dong YH, Song JP, Tang AJ, Feng GQ (1999) *Tragulus javanicus williamsoni* Kloss habitat selection in Xishuangbanna Nature Reserve: A preliminary study. Yunnan Forestry Science and Technology 1999: 67-71. <https://doi.org/10.16473/j.cnki.xblykx1972.1999.02.013> (in Chinese)

Lv T, Zheng X, Zhu GF, Tang YJ, Jiang GL, Li ZL, Chen MY (2019) Dynamics analysis of Xishuangbanna Mengla Asian elephant population. Forestry Construction 2019: 91-96. (in Chinese)

Ma Z, He Z, Wang Y, Song D, Xia F, Cui S, Su H, Deng J, Li P, Li S (2022) An update on the current distribution and key habitats of the clouded leopard (*Neofelis nebulosa*) populations in China. Biodiv Sci 30: 59-73. <https://doi.org/10.17520/biods.2022349> (in Chinese)

Pan W, Lin L, Luo A, Zhang L (2009) Corridor use by Asian elephants. Integr Zool 4: 220-231. <https://doi.org/10.1111/j.1749-4877.2009.00154.x>

Shen QZ, Wang B, Bao MW, Xiao Y, Ai HL (2021) Monitoring of Asian elephant activities in dry season in Sancha River of the National Reserve. Agriculture and Technology 41: 111-114. <https://doi.org/10.19754/j.nyyjs.20210228031>. (in Chinese)

Shen QZ, Wang B, Bao MW, Xiong CY, Xiao Y (2021) Preliminary study on rescue work of wild Asian elephant in Xishuangbanna. China Animal Health 23: 129-131. <https://doi.org/10.3969/j.issn.1008-4754.2021.04.087>. (in Chinese)

Song DZ (2022) Wildlife-New discoveries of infrared cameras. Forest & Humankind 2022: 28-33. (in Chinese)

Song DZ, Xiao SB, Xiao F (2021) Mainland Leopard Cat, Marbled Cat, Asiatic Golden Cat, Chinese Mountain Cat, Jungle Cat, why they are "upgraded". Forest & Humankind 2021: 72-81. (in Chinese)

Song DZ, Wang Z.S (2016) Clouded leopard reappearance. Forest & Humankind 2016: 142-145. (in Chinese)

Song ZY, Luo JS, Liu TJ, Ai B (2020) Investigation on *Macaca mulatta* population in Xishuangbanna. Forest Inventory and Planning 40: 75-79. <https://doi.org/10.3969/j.issn.1671-3168.2020.05.013> (in Chinese)

Song ZY, Wen SR, Li J (2020) Population distribution of *Macaca leonina* in Xishuangbanna. Forest Inventory and Planning 45: 68-71. <https://doi.org/10.3969/j.issn.1671-3168.2020.04.013> (in Chinese)

Song ZY, Yang HP, Yang ZB, Yu DL, Yang ZC (2017) Population status and conservation of *Nomascus leucogenys* in Xishuangbanna. Journal of West China Forestry Science 46: 18-22+27. <https://doi.org/10.16473/j.cnki.xblykx1972.2017.03.004> (in Chinese)

Song ZY, Yu DL, Yang HP, Yang ZB (2020) Resources investigation of Ceropithecidae in Xishuangbanna. Forest Inventory and Planning 45: 82-88. <https://doi.org/10.3969/j.issn.1671-3168.2020.03.016> (in Chinese)

Su K, Ren J, Yang J, Hou Y, Wen Y (2020) Human-Elephant conflicts and villagers’ attitudes and knowledge in the Xishuangbanna Nature Reserve, China. Int J Environ Res Public Health 17: 8910. <https://doi.org/10.3390/ijerph17238910>

Sun N, Cao G, Li G, Liu Z, Quan RC (2020) *Macaca leonina* has a wider niche breadth than sympatric *M. mulatta* in a fragmented tropical forest in southwest China. Am J Primatol 82: e23100. <https://doi.org/10.1002/ajp.23100>

Tang R, Li W, Zhu D, Shang X, Guo X, Zhang L (2020) Raging elephants: effects of human disturbance on physiological stress and reproductive potential in wild Asian elephants. Conserv Physiol 8: coz106. <https://doi.org/10.1093/conphys/coz106>

Tongkok S, He X, Alcantara MJM, Saralamba C, Nathalang A, Chanthorn W, Brockelman WY, Lin L (2020) Composition of frugivores of *Baccaurea ramiflora* (Phyllanthaceae) and effects of environmental factors on frugivory in two tropical forests of China and Thailand. Glob Ecol Conserv 23: e01096. <https://doi.org/10.1016/j.gecco.2020.e01096>

Tongkok S, Yuan SD, Alcantara MJM, Deng XB, Guo XM, He XL, Lin LX (2019) Owston's Civet (*Chrotogale owstoni*) Found in Xishuangbanna, Yunnan Province. Chinese Journal of Zoology 54: 603-604. <https://doi.org/10.13859/j.cjz.201904021> (in Chinese)

Wang B, Tao Q, Yang SJ (2007) The impact of Asian elephants and other wildlife species on the villages around the Shangyong Nature Reserve, Xishuangbanna. Ecological Economy 2007: 31-34. (in Chinese)

Wang QY, He YC, Zhang MX, Song JP, Tao YX, Zhang ZY, Ai ZJ, Yang HP (2018) Application of infrared cameras for wildlife surveys in the Bulong Nature Reserve, Xishuangbanna Prefecture, Yunnan Province. Acta Theriologica Sinica 38: 513-518. <https://doi.org/10.16829/j.slxb.150143> (in Chinese)

Wang QY, Tao YX, Li JS, Dong Z, Ai B, Yang PH (2017) Factors on transition of distribution of the wild Asian Elephant in Xishuangbanna. Forest Inventory and Planning 42: 113-118. <https://doi.org/10.3969/j.issn.1671-3168.2017.04.025> (in Chinese)

Wang YX (2003) A complete list and distribution of Mammalian species and subspecies in China. China Forestry Press, Beijing. (in Chinese)

Wang ZH, Zhang HZ, Zhao WL, Zheng X, Ai WB, Li ZL, Chen MY (2019) Dynamics analysis of Xishuangbanna Menghai-Puer Lancang Asian elephant population. Forestry Construction 2019: 79-84. (in Chinese)

Wang ZJ, Chen J, Deng XB, Bai ZL, Yang Q, Liu ZQ, Liu Y (2000) The relationship between *Choerospondias axillaris* and wildlife in Xishuangbanna. Journal of Northeast Forestry University 2000: 55-57. <https://doi.org/10.13759/j.cnki.dlxb.2000.06.014> (in Chinese)

Wang ZQ (2006) Xishuangbanna National Nature Reserve. Yunnan Education Press, Kunming. (in Chinese)

Wei M (2009) The rare and endangered sun bear. Grand Garden of Science, 2009: 6.

Wu J, Han JQ, Shi LQ, Zou Y, Li Z, Yang JF, Huang CQ, Zou FC (2018) Prevalence, genotypes, and risk factors of *Enterocytozoon bieneusi* in Asiatic black bear (*Ursus thibetanus*) in Yunnan Province, Southwestern China. Parasitol Res 117: 1139-1145. <https://doi.org/10.1007/s00436-018-5791-0>

Wu JL, Jiang WG, Hu JS, Li ZQ, Li CH (1999) Distribution changes of Asian elephants in Xishuangbanna during the last 40 years. Chinese Journal of Wildlife 1999: 8-9. <https://doi.org/10.19711/j.cnki.issn2310-1490.1999.03.007> (in Chinese)

Xiong YX (1996) Discussion on the protection of wild Asian elephant in Xishuangbanna. Man and the Biosphere, 1996: 27-29. (in Chinese)

Xu Y, Jiang H, Quan F (1987) Overview of mammals in Xishuangbanna Nature PAs. Pp. 289-311 in Synthesis of biodiversity field surveys in Xishuangbanna Nature Reserves. Kunming, Yunnan Scientific Technology Publisher, China. (in Chinese)

Yang DH (1993) Xishuangbanna Fauna. Yunnan University Press, Kunming. (in Chinese)

Yang DH, Xu PS (1988) Observations on the white-cheeked gibbon of Xishuangbanna. Sichuan Journal of Zoology 7: 36-38. (in Chinese)

Yang DH, Zhang JY, Li C (1988) The population distribution of *Bos* in Yunnan. Chinese Journal of Zoology 1988: 36-38+54. <https://doi.org/10.13859/j.cjz.1988.01.015> (in Chinese)

Yang HP, Song ZY, Yang ZJ, Yang ZB, Li JS (2017) Study on population distribution of *Tragulus javanicus* in Xishuangbanna. Forest Inventory and Planning 42: 23-27+36. <https://doi.org/10.3969/j.issn.1671-3168.2017.04.005> (in Chinese)

Yang Z, Chen Y, Li J, Wang L, Piao Y, Song Z, Shi K (2018) Individual identification and population size assessment for Asian elephant based on camera-trapping techniques. Acta Theriologica Sinica 38: 18-27. <https://doi.org/10.16829/j.slxb.150109> (in Chinese)

Yin F, Ma K, Liu DX (2015) Population status, ecological habits and threatened factors of Wild gaur (*Bos gaurus*): a review. Journal of Beijing Normal University (Natural Science) 51: 504-510. (in Chinese)

Yuan SD, Deng Y, Luo AD, Guo XM, Wen MC, Wang B, Ze D, Yang F, Wen SR (2019) A preliminary study of wireless infrared camera technology in alleviating the human-elephant conflict. Forestry construction 2019: 61-66. (in Chinese)

Yuan ZQ, Zhang L (2006) Population and activity characteristic of wild Asian elephants in the Wild Elephant Valley, Xishuangbanna National Nature Reserve Yunnan, China. Acta Theriologica Sinica 26: 359-367. <https://doi.org/10.16829/j.slxb.2006.04.007> (in Chinese)

Zhang HL, Wang RC, Li ZX (1999) GIS-based habitat types and their relationship with survival of *Bos gaurus readei*. Chinese Journal of Applied Ecology 10: 619-622. <https://doi.org/10.13287/j.1001-9332.1999.0162> (in Chinese)

Zhang MX, Cao L, Quan RC, Xiao ZS, Yang XF, Zhang WF, Wang XZ, Deng XB (2014) Camera trap survey of animals in Xishuangbanna Forest Dynamics Plot, Yunnan. Biodiv Sci 22: 830-832. <https://doi.org/10.3724/SP.J.1003.2014.14064> (in Chinese)

Zhang ZY, Yang HP, Luo QY, Zhang L (2018) Research status of *Bos gaurus* in Xishuangbanna Prefecture. Forestry Inventory and Planning 43: 117-119. <https://doi.org/10.3969/j.issn.1671-3168.2018.02.021> (in Chinese)

Zhu GF, Zheng X, Lv T, Jiang GL, Tang YJ, Li ZL, Chen MY (2019) A dynamics analysis of Xishuangbanna-Puer Asian elephant population. Forestry Construction 2019: 85-90. (in Chinese)

| Table S1. List of MengLun sub-reserve’s large and medium-sized mammals from camera traps | | | |
| --- | --- | --- | --- |
|  | Species | English name | IUCN red list |
|  | **Primates** |  |  |
|  | **Cercopithedidae** |  |  |
| 1 | Macaca assamensis | Assamese Macaque | NT |
| 2 | Macaca leonina | Northern Pig-tailed Macaque | VU |
| 3 | Macaca mulatta | Rhesus Monkey | LC |
|  |  |  |  |
|  | **Pholidota** |  |  |
|  | **Manidae** |  |  |
| 4 | Manis pentadactyla | Chinese Pangolin | CR |
|  |  |  |  |
|  | **Carnivora** |  |  |
|  | **Canidae** |  |  |
| 5 | Cuon alpinus | Dhole | EN |
|  | **Ursidae** |  |  |
| 6 | Ursus thibetanus | Asiatic Black Bear | VU |
|  | **Mustelidae** |  |  |
| 7 | Arctonyx collaris | Greater Hog Badger | VU |
| 8 | Martes flavigula | Yellow-throated Marten | LC |
| 9 | Melogale moschata | Small-toothed Ferret Badger | LC |
| 10 | *Mustela strigidorsa* | Stripe-backed Weasel | LC |
|  | **Viverridae** |  |  |
| 11 | Arctictis binturong | Binturong | VU |
| 12 | Paguma larvata | Masked Palm Civet | LC |
| 13 | Paradoxurus hermaphroditus | Common Palm Civet | LC |
| 14 | Prionodon pardicolor | Spotted Linsang | LC |
| 15 | *Viverra megaspila* | Large-spotted Civet | EN |
| 16 | *Viverricula indica* | Small Indian Civet | LC |
|  | **Herpestidae** |  |  |
| 17 | Herpestes urva |  | LC |
|  | **Felidae** |  |  |
| 18 | Prionailurus bengalensis | Crab-eating Mongoose | LC |
|  |  |  |  |
|  | **Cet**artiodactyla |  |  |
|  | **Suidae** |  |  |
| 19 | Sus scrofa | Wild Boar | LC |
|  | **Cervidae** |  |  |
| 20 | Muntiacus vaginalis | Northern Red Muntjac | LC |
|  | Bovidae |  |  |
| 21 | Capricornis milneedwardsii | Mainland Serow | VU |
|  |  |  |  |
|  | **Rodentia** |  |  |
|  | **Spalacidae** |  |  |
| 22 | *Rhizomys sumatrensis* | Indomalayan Bamboo Rat | LC |
|  | **Hystricidae** |  |  |
| 23 | Atherurus macrourus | Asiatic Brush-tailed Porcupine | LC |
| 24 | Hystrix brachyura | Malayan Porcupine | LC |

IUCN red list: LC: Least Concern; NT: Near Threatened; EN: Endangered; VU: Vulnerable; CR: Critically Endangered

| Table S2. List of MengLa sub-reserve’s large and medium-sized mammals from camera traps | | | |
| --- | --- | --- | --- |
|  | Species | English name | IUCN red list |
|  | **Primates** |  |  |
|  | **Cercopithedidae** |  |  |
| 1 | *Macaca assamensis* | Assamese Macaque | NT |
| 2 | *Macaca leonina* | Northern Pig-tailed Macaque | VU |
| 3 | *Macaca mulatta* | Rhesus Monkey | LC |
|  |  |  |  |
|  | **Carnivora** |  |  |
|  | **Canidae** |  |  |
| 4 | *Cuon alpinus* | Dhole | EN |
|  | **Ursidae** |  |  |
| 5 | *Ursus thibetanus* | Asiatic Black Bear | VU |
|  | **Mustelidae** |  |  |
| 6 | *Arctonyx collaris* | Greater Hog Badger | VU |
| 7 | *Martes flavigula* | Yellow-throated Marten | LC |
| 8 | *Melogale moschata* | Small-toothed Ferret Badger | LC |
| 9 | *Mustela strigidorsa* | Stripe-backed Weasel | LC |
|  | **Viverridae** |  |  |
| 10 | Arctictis binturong | Binturong | VU |
| 11 | *Chrotogale owstoni* | Owston’s Civet | EN |
| 12 | *Paguma larvata* | Masked Palm Civet | LC |
| 13 | *Paradoxurus hermaphroditus* | Common Palm Civet | LC |
| 14 | *Prionodon pardicolor* | Spotted Linsang | LC |
| 15 | *Viverra zibetha* | Large Indian Civet | LC |
| 16 | *Viverricula indica* | Small Indian Civet | LC |
|  | **Herpestidae** |  |  |
| 17 | *Herpestes urva* | Crab-eating Mongoose | LC |
|  | **Felidae** |  |  |
| 18 | *Pardofelis temminckii* | Asiatic Golden Cat | NT |
| 19 | *Neofelis nebulosa* | Clouded Leopard | VU |
| 20 | *Pardofelis marmorata* | Marbled Cat | NT |
| 21 | *Prionailurus bengalensis* | Mainland Leopard Cat | LC |
|  |  |  |  |
|  | Proboscidea |  |  |
|  | **Elephantidae** |  |  |
| 22 | Elephas maximus | Asian Elephant | EN |
|  | **Cet**artiodactyla |  |  |
|  | **Suidae** |  |  |
| 23 | *Sus scrofa* | Wild Boar | LC |
|  | **Tragulidae** |  |  |
| 24 | *Tragulus williamsoni* | Williamson’s Chevrotain | DD |
|  | **Cervidae** |  |  |
| 25 | *Muntiacus vaginalis* | Northern Red Muntjac | LC |
| 26 | *Rusa unicolor* | Sambar | VU |
|  | **Bovidae** |  |  |
| 27 | *Capricornis milneedwardsii* | Mainland Serow | VU |
|  |  |  |  |
|  | **Rodentia** |  |  |
|  | **Sciuridae** |  |  |
| 28 | *Ratufa bicolor* | Black Giant Squirrel | NT |
|  | **Spalacidae** |  |  |
| 29 | *Rhizomys pruinosus* | Bamboo Rat | LC |
| 30 | *Rhizomys sumatrensis* | Indomalayan Bamboo Rat | LC |
|  | **Hystricidae** |  |  |
| 31 | *Atherurus macrourus* | Asiatic Brush-tailed Porcupine | LC |
| 32 | *Hystrix* brachyura | Malayan Porcupine | LC |

IUCN red list: LC: Least Concern; NT: Near Threatened; EN: Endangered; VU: Vulnerable; DD: Data Deficient

| Table S3. List of ShangYong sub-reserve’s large and medium-sized mammals from camera traps | | | |
| --- | --- | --- | --- |
|  | Species | English name | IUCN red list |
|  | **Primates** |  |  |
|  | **Cercopithedidae** |  |  |
| 1 | Macaca assamensis | Assamese Macaque | NT |
| 2 | *Macaca leonina* | Northern Pig-tailed Macaque | VU |
| 3 | *Macaca mulatta* | Rhesus Monkey | LC |
|  |  |  |  |
|  | **Carnivora** |  |  |
|  | **Canidae** |  |  |
| 4 | Cuon alpinus | Dhole | EN |
|  | **Ursidae** |  |  |
| 5 | *Helarctos malayanus* | Sun Bear | VU |
| 6 | *Ursus thibetanus* | Asiatic Black Bear | VU |
|  | **Mustelidae** |  |  |
| 7 | Arctonyx collaris | Greater Hog Badger | VU |
| 8 | *Martes flavigula* | Yellow-throated Marten | LC |
| 9 | *Melogale moschata* | Small-toothed Ferret Badger | LC |
| 10 | *Mustela strigidorsa* | Siberian Weasel | LC |
|  | **Viverridae** |  |  |
| 11 | *Arctictis binturong* | Binturong | VU |
| 12 | *Paguma larvata* | Masked Palm Civet | LC |
| 13 | Paradoxurus hermaphroditus | Common Palm Civet | LC |
| 14 | *Prionodon pardicolor* | Spotted Linsang | LC |
| 15 | Viverra zibetha | Large Indian Civet | LC |
|  | **Herpestidae** |  |  |
| 16 | *Herpestes urva* | Crab-eating Mongoose | LC |
|  | **Felidae** |  |  |
| 17 | *Pardofelis temminckii* | Asiatic Golden Cat | NT |
| 18 | *Neofelis nebulosa* | Clouded Leopard | VU |
| 19 | *Prionailurus bengalensis* | Mainland Leopard Cat | LC |
|  |  |  |  |
|  | **Proboscidea** |  |  |
|  | **Elephantidae** |  |  |
| 20 | *Elephas maximus* | Asian Elephant | EN |
|  |  |  |  |
|  | **Cet**artiodactyla |  |  |
|  | **Suidae** |  |  |
| 21 | *Sus scrofa* | Wild Boar | LC |
|  | **Tragulidae** |  |  |
| 22 | *Tragulus williamsoni* | Williamson’s Chevrotain | DD |
|  | **Cervidae** |  |  |
| 23 | *Muntiacus vaginalis* | Northern Red Muntjac | LC |
| 24 | *Rusa unicolor* | Sambar | VU |
|  | **Bovidae** |  |  |
| 25 | *Capricornis milneedwardsii* | Mainland Serow | VU |
|  |  |  |  |
|  | **Rodentia** |  |  |
|  | **Sciuridae** |  |  |
| 26 | *Ratufa bicolor* | Black Giant Squirrel | NT |
|  | **Spalacidae** |  |  |
| 27 | *Rhizomys sumatrensis* | Indomalayan Bamboo Rat | LC |
|  | **Hystricidae** |  |  |
| 28 | *Atherurus macrourus* | Asiatic Brush-tailed Porcupine | LC |
| 29 | *Hystrix* brachyura | Malayan Porcupine | LC |

IUCN red list: LC: Least Concern; NT: Near Threatened; EN: Endangered; VU: Vulnerable; DD: Data Deficient

| Table S4. List of MengYang sub-reserve’s large and medium-sized mammals from this survey | | | |
| --- | --- | --- | --- |
|  | Species | English name | IUCN red list |
|  | **Primates** |  |  |
|  | **Cercopithedidae** |  |  |
| 1 | Macaca assamensis | Assamese Macaque | NT |
| 2 | *Macaca leonina* | Northern Pig-tailed Macaque | VU |
| 3 | *Macaca mulatta* | Rhesus Monkey | LC |
|  |  |  |  |
|  | **Pholidota** |  |  |
|  | **Manidae** |  |  |
| 4 | Manis pentadactyla | Chinese Pangolin | CR |
|  |  |  |  |
|  | **Carnivora** |  |  |
|  | **Canidae** |  |  |
| 5 | *Cuon alpinus* | Dhole | EN |
|  | **Ursidae** |  |  |
| 6 | *Ursus thibetanus* | Asiatic Black Bear | VU |
|  | **Mustelidae** |  |  |
| 7 | *Arctonyx collaris* | Greater Hog Badger | VU |
| 8 | *Martes flavigula* | Yellow-throated Marten | LC |
| 9 | *Melogale moschata* | Small-toothed Ferret Badger | LC |
| 10 | *Mustela strigidorsa* | Stripe-backed Weasel | LC |
|  | **Viverridae** |  |  |
| 11 | *Arctictis binturong* | Binturong | VU |
| 12 | *Paguma larvata* | Masked Palm Civet | LC |
| 13 | *Paradoxurus hermaphroditus* | Common Palm Civet | LC |
| 14 | *Prionodon pardicolor* | Spotted Linsang | LC |
| 15 | *Viverricula indica* | Small Indian Civet | LC |
|  | **Herpestidae** |  |  |
| 16 | *Herpestes urva* | Crab-eating Mongoose | LC |
|  | **Felidae** |  |  |
| 17 | Neofelis nebulosa | Clouded Leopard | VU |
| 18 | *Prionailurus bengalensis* | Mainland Leopard Cat | LC |
|  |  |  |  |
|  | **Proboscidea** |  |  |
|  | **Elephantidae** |  |  |
| 19 | *Elephas maximus* | Asian Elephant | EN |
|  |  |  |  |
|  | **Cet**artiodactyla |  |  |
|  | **Suidae** |  |  |
| 20 | *Sus scrofa* | Wild Boar | LC |
|  | **Cervidae** |  |  |
| 21 | *Muntiacus vaginalis* | Northern Red Muntjac | LC |
| 22 | *Rusa unicolor* | Sambar | VU |
|  | **Bovidae** |  |  |
| 23 | *Bos gaurus* | Gaur | VU |
| 24 | *Capricornis milneedwardsii* | Mainland Serow | VU |
|  |  |  |  |
|  | **Rodentia** |  |  |
|  | **Sciuridae** |  |  |
| 25 | *Ratufa bicolor* | Black Giant Squirrel | NT |
|  | **Spalacidae** |  |  |
| 26 | *Rhizomys sumatrensis* | Indomalayan Bamboo Rat | LC |
|  | **Hystricidae** |  |  |
| 27 | *Atherurus macrourus* | Asiatic Brush-tailed Porcupine | LC |
| 28 | *Hystrix* brachyura | Malayan Porcupine | LC |

IUCN red list: LC: Least Concern; NT: Near Threatened; EN: Endangered; VU: Vulnerable; CR: Critically Endangered

| Table S5. List of ManGao sub-reserve’s large and medium-sized mammals from camera traps | | | |
| --- | --- | --- | --- |
|  | Species | English name | IUCN red list |
|  | **Primates** |  |  |
|  | **Cercopithedidae** |  |  |
| 1 | *Macaca leonina* | Northern Pig-tailed Macaque | VU |
| 2 | Macaca mulatta | Rhesus Monkey | LC |
|  |  |  |  |
|  | **Carnivora** |  |  |
|  | **Mustelidae** |  |  |
| 3 | *Martes flavigula* | Yellow-throated Marten | LC |
| 4 | *Melogale moschata* | Small-toothed Ferret Badger | LC |
|  | **Viverridae** |  |  |
| 5 | *Paguma larvata* | Masked Palm Civet | LC |
| 6 | *Viverra zibetha* | Large Indian Civet | LC |
| 7 | Viverricula indica | Small Indian Civet | LC |
|  | **Felidae** |  |  |
| 8 | *Prionailurus bengalensis* | Mainland Leopard Cat | LC |
|  |  |  |  |
|  | **Cet**artiodactyla |  |  |
|  | **Suidae** |  |  |
| 9 | *Sus scrofa* | Wild Boar | LC |
|  | **Cervidae** |  |  |
| 10 | *Muntiacus vaginalis* | Northern Red Muntjac | LC |
|  | Bovidae |  |  |
| 11 | *Bos gaurus* | Gaur | VU |
|  |  |  |  |
|  | **Rodentia** |  |  |
|  | **Hystricidae** |  |  |
| 12 | *Hystrix* brachyura | Malayan Porcupine | LC |

IUCN red list: LC: Least Concern; NT: Near Threatened; EN: Endangered; VU: Vulnerable

| Table S6. List of Bulong Prefectural Nature Reserve’s large and medium-sized mammals from camera traps | | | |
| --- | --- | --- | --- |
|  | Species | English name | IUCN red list |
|  | Primates |  |  |
| 1 | Macaca leonina | Northern Pig-tailed Macaque | VU |
|  |  |  |  |
|  | **Carnivora** |  |  |
|  | **Ursidae** |  |  |
| 2 | Ursus thibetanus | Asiatic Black Bear | VU |
|  | **Mustelidae** |  |  |
| 4 | *Martes flavigula* | Yellow-throated Marten | LC |
| 5 | *Melogale moschata* | Small-toothed Ferret Badger | LC |
| 6 | *Mustela strigidorsa* | Stripe-backed Weasel | LC |
|  | **Viverridae** |  |  |
| 7 | *Paguma larvata* | Masked Palm Civet | LC |
| 8 | Paradoxurus hermaphroditus | Common Palm Civet | LC |
| 9 | Prionodon pardicolor | Spotted Linsang | LC |
|  | Herpestidae |  |  |
| 3 | *Herpestes urva* | Crab-eating Mongoose | LC |
|  | **Felidae** |  |  |
| 10 | *Prionailurus bengalensis* | Mainland Leopard Cat | LC |
|  |  |  |  |
|  | **Cet**artiodactyla |  |  |
|  | **Suidae** |  |  |
| 11 | *Sus scrofa* | Wild Boar | LC |
|  | **Cervidae** |  |  |
| 12 | *Muntiacus vaginalis* | Northern Red Muntjac | LC |
| 13 | Rusa unicolor | Sambar | VU |
|  | **Bovidae** |  |  |
| 14 | Capricornis milneedwardsii | Mainland Serow | VU |
|  |  |  |  |
|  | **Rodentia** |  |  |
|  | **Hystricidae** |  |  |
| 15 | Atherurus macrourus | Asiatic Brush-tailed Porcupine | LC |
| 16 | Hystrix brachyura | Malayan Porcupine | LC |

IUCN red list: LC: Least Concern; NT: Near Threatened; VU: Vulnerable

| Table S7. List of Yiwu Prefectural Nature Reserve’s large and medium-sized mammals from camera traps | | | |
| --- | --- | --- | --- |
|  | Species | English name | IUCN red list |
|  | **Primates** |  |  |
|  | **Cercopithedidae** |  |  |
| 1 | Macaca assamensis | Assamese Macaque | NT |
| 2 | *Macaca leonina* | Northern Pig-tailed Macaque | VU |
| 3 | Macaca mulatta | Rhesus Monkey | LC |
|  |  |  |  |
|  | **Carnivora** |  |  |
|  | **Canidae** |  |  |
| 4 | *Cuon alpinus* | Dhole | EN |
|  | **Ursidae** |  |  |
| 5 | *Helarctos malayanus* | Sun Bear | VU |
| 6 | Ursus thibetanus | Asiatic Black Bear | VU |
|  | **Mustelidae** |  |  |
| 7 | Arctonyx collaris | Greater Hog Badger | VU |
| 8 | *Martes flavigula* | Yellow-throated Marten | LC |
| 9 | *Melogale moschata* | Small-toothed Ferret Badger | LC |
| 10 | *Mustela strigidorsa* | Stripe-backed Weasel | LC |
|  | **Viverridae** |  |  |
| 11 | *Arctictis binturong* | Binturong | VU |
| 12 | *Chrotogale owstoni* | Owston’s Civet | EN |
| 13 | *Paguma larvata* | Masked Palm Civet | LC |
| 14 | Paradoxurus hermaphroditus | Common Palm Civet | LC |
| 15 | Prionodon pardicolor | Spotted Linsang | LC |
| 16 | *Viverra zibetha* | Large Indian Civet | LC |
| 17 | Viverricula indica | Small Indian Civet | LC |
|  | **Herpestidae** |  |  |
| 18 | *Herpestes urva* | Crab-eating Mongoose | LC |
|  | **Felidae** |  |  |
| 19 | *Pardofelis temminckii* | Asiatic Golden Cat | NT |
| 20 | *Neofelis nebulosa* | Clouded Leopard | VU |
| 21 | *Panthera pardus* | Leopard | VU |
| 22 | *Pardofelis marmorata* | Marbled Cat | NT |
| 23 | *Prionailurus bengalensis* | Mainland Leopard Cat | LC |
|  |  |  |  |
|  | Proboscidea |  |  |
|  | **Elephantidae** |  |  |
| 24 | *Elephas maximus* | Asian Elephant | EN |
|  |  |  |  |
|  | **Cet**artiodactyla |  |  |
|  | **Suidae** |  |  |
| 25 | *Sus scrofa* | Wild Boar | LC |
|  | **Tragulidae** |  |  |
| 26 | *Tragulus williamsoni* | Williamson’s Chevrotain | DD |
|  |  |  |  |
|  | **Cervidae** |  |  |
| 27 | *Muntiacus vaginalis* | Northern Red Muntjac | LC |
|  | **Bovidae** |  |  |
| 28 | *Capricornis milneedwardsii* | Mainland Serow | VU |
|  |  |  |  |
|  | **Rodentia** |  |  |
|  | **Sciuridae** |  |  |
| 29 | *Ratufa bicolor* | Black Giant Squirrel | NT |
|  | **Spalacidae** |  |  |
| 30 | *Rhizomys sumatrensis* |  | LC |
|  | **Hystricidae** |  |  |
| 31 | Atherurus macrourus | Asiatic Brush-tailed Porcupine | LC |
| 32 | *Hystrix* brachyura | Malayan Porcupine | LC |

IUCN red list: LC: Least Concern; NT: Near Threatened; EN: Endangered; VU: Vulnerable; DD: Data Deficient

| Table S8. List of Nabanhe Basin National Nature Reserve’s large and medium-sized mammals from camera traps | | | |
| --- | --- | --- | --- |
|  | Species | English name | IUCN red list |
|  | **Primates** |  |  |
|  | **Cercopithedidae** |  |  |
| 1 | Macaca assamensis | Assamese Macaque | NT |
| 2 | *Macaca leonina* | Northern Pig-tailed Macaque | VU |
| 3 | Macaca mulatta | Rhesus Monkey | LC |
|  |  |  |  |
|  | **Carnivora** |  |  |
|  | **Canidae** |  |  |
| 4 | *Cuon alpinus* | Dhole | EN |
|  | **Ursidae** |  |  |
| 5 | Ursus thibetanus | Asiatic Black Bear | VU |
|  | **Mustelidae** |  |  |
| 6 | Arctonyx collaris | Greater Hog Badger | VU |
| 7 | *Martes flavigula* | Yellow-throated Marten | LC |
| 8 | *Melogale moschata* | Small-toothed Ferret Badger | LC |
|  | **Viverridae** |  |  |
| 9 | *Paguma larvata* | Masked Palm Civet | LC |
| 10 | Paradoxurus hermaphroditus | Common Palm Civet | LC |
| 11 | Prionodon pardicolor | Spotted Linsang | LC |
|  | **Herpestidae** |  |  |
| 12 | *Herpestes urva* | Crab-eating Mongoose | LC |
|  | **Felidae** |  |  |
| 13 | *Catopuma temminckii* | Asiatic Golden Cat | NT |
| 14 | *Prionailurus bengalensis* | Mainland Leopard Cat | LC |
|  | **Artiodactyla** |  |  |
|  | **Suidae** |  |  |
| 15 | *Sus scrofa* | Wild Boar | LC |
|  | **Cervidae** |  |  |
| 16 | *Muntiacus* vaginalis | Northern Red Muntjac | LC |
| 17 | Rusa unicolor | Sambar | VU |
|  | **Bovidae** |  |  |
| 18 | *Bos gaurus* | Gaur | VU |
| 19 | *Capricornis milneedwardsii* | Mainland Serow | VU |
| 20 | *Naemorhedus evansi* | Burmese Goral |  |
|  |  |  |  |
|  | **Rodentia** |  |  |
|  | **Sciuridae** |  |  |
| 21 | *Ratufa bicolor* | Black Giant Squirrel | NT |
|  | **Hystricidae** |  |  |
| 22 | Atherurus macrourus | Asiatic Brush-tailed Porcupine | LC |
| 23 | *Hystrix* brachyura | Malayan Porcupine | LC |

IUCN red list: LC: Least Concern; NT: Near Threatened; EN: Endangered; VU: Vulnerable

| Table S9. List of State Forest Farm’s large and medium-sized mammals from this camera traps | | | |
| --- | --- | --- | --- |
|  | Species | English name | IUCN red list |
|  | **Primates** |  |  |
|  | **Cercopithedidae** |  |  |
| 1 | Macaca assamensis | Assamese Macaque | NT |
| 2 | *Macaca leonina* | Northern Pig-tailed Macaque | VU |
| 3 | Macaca mulatta | Rhesus Monkey | LC |
|  |  |  |  |
|  | **Carnivora** |  |  |
|  | **Ursidae** |  |  |
| 5 | Ursus thibetanus | Asiatic Black Bear | VU |
|  | **Mustelidae** |  |  |
| 6 | *Martes flavigula* | Yellow-throated Marten | LC |
| 7 | *Melogale moschata* | Small-toothed Ferret Badger | LC |
| 8 | *Mustela strigidorsa* | Stripe-backed Weasel | LC |
|  | **Viverridae** |  |  |
| 9 | *Paguma larvata* | Masked Palm Civet | LC |
| 10 | Paradoxurus hermaphroditus | Common Palm Civet | LC |
| 11 | Prionodon pardicolor | Spotted Linsang | LC |
|  | **Herpestidae** |  |  |
| 4 | *Herpestes urva* | Crab-eating Mongoose | LC |
|  | **Felidae** |  |  |
| 12 | *Prionailurus bengalensis* | Mainland Leopard Cat | LC |
|  |  |  |  |
|  | **Cet**artiodactyla |  |  |
|  | **Suidae** |  |  |
| 13 | *Sus scrofa* | Wild Boar | LC |
|  | **Cervidae** |  |  |
| 14 | *Muntiacus vaginalis* | Northern Red Muntjac | LC |
|  |  |  |  |
|  | **Rodentia** |  |  |
|  | **Sciuridae** |  |  |
| 15 | *Ratufa bicolor* | Black Giant Squirrel | NT |
|  | **Hystricidae** |  |  |
| 16 | *Hystrix* brachyura | Malayan Porcupine | LC |

IUCN red list: LC: Least Concern; NT: Near Threatened; VU: Vulnerable
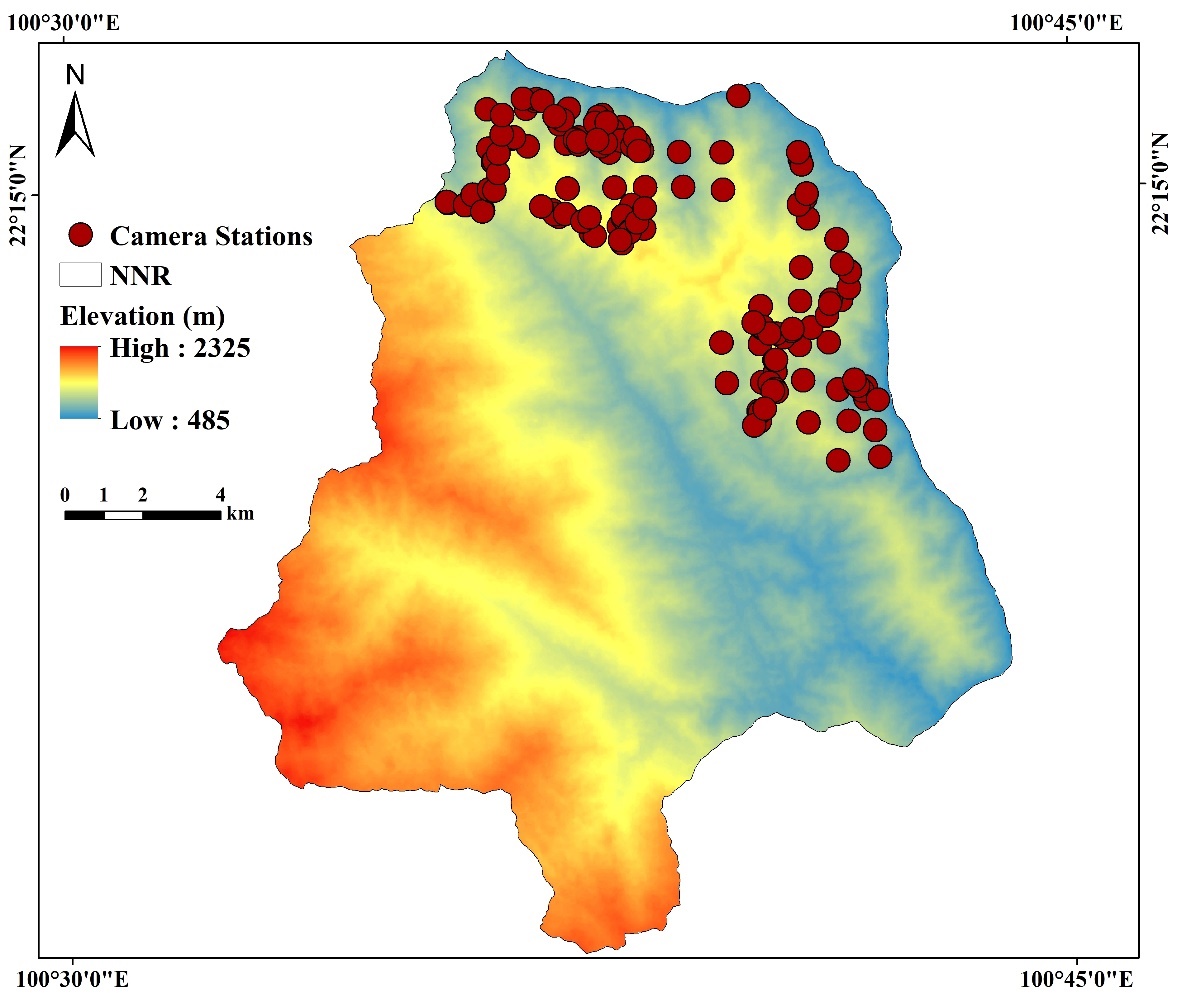


Fig. S1. The elevation range of NNR. NNR represents Nabanhe Basin National Nature Reserve.


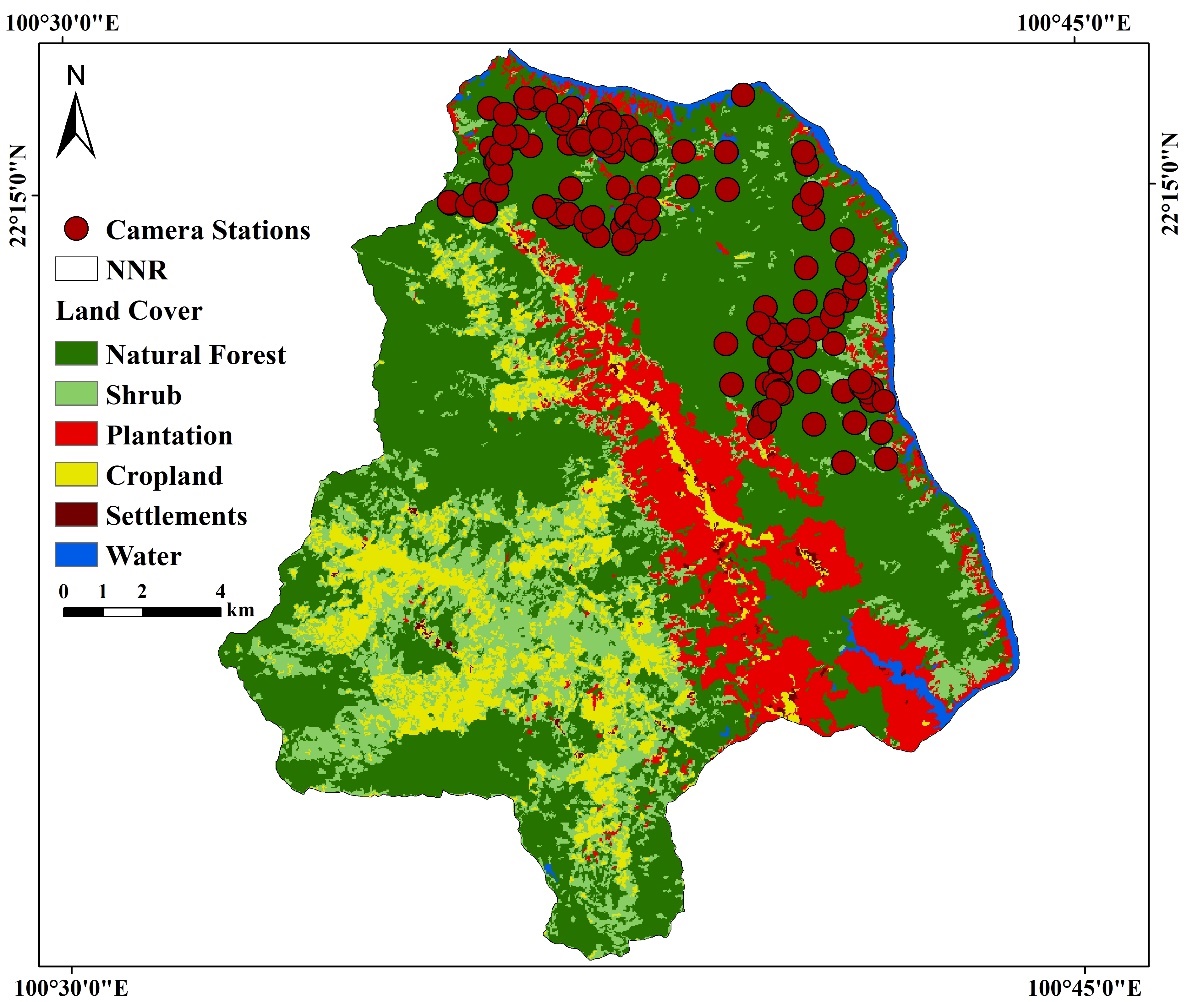


Fig. S2. The land use types of NNR. NNR represents Nabanhe Basin National Nature Reserve.


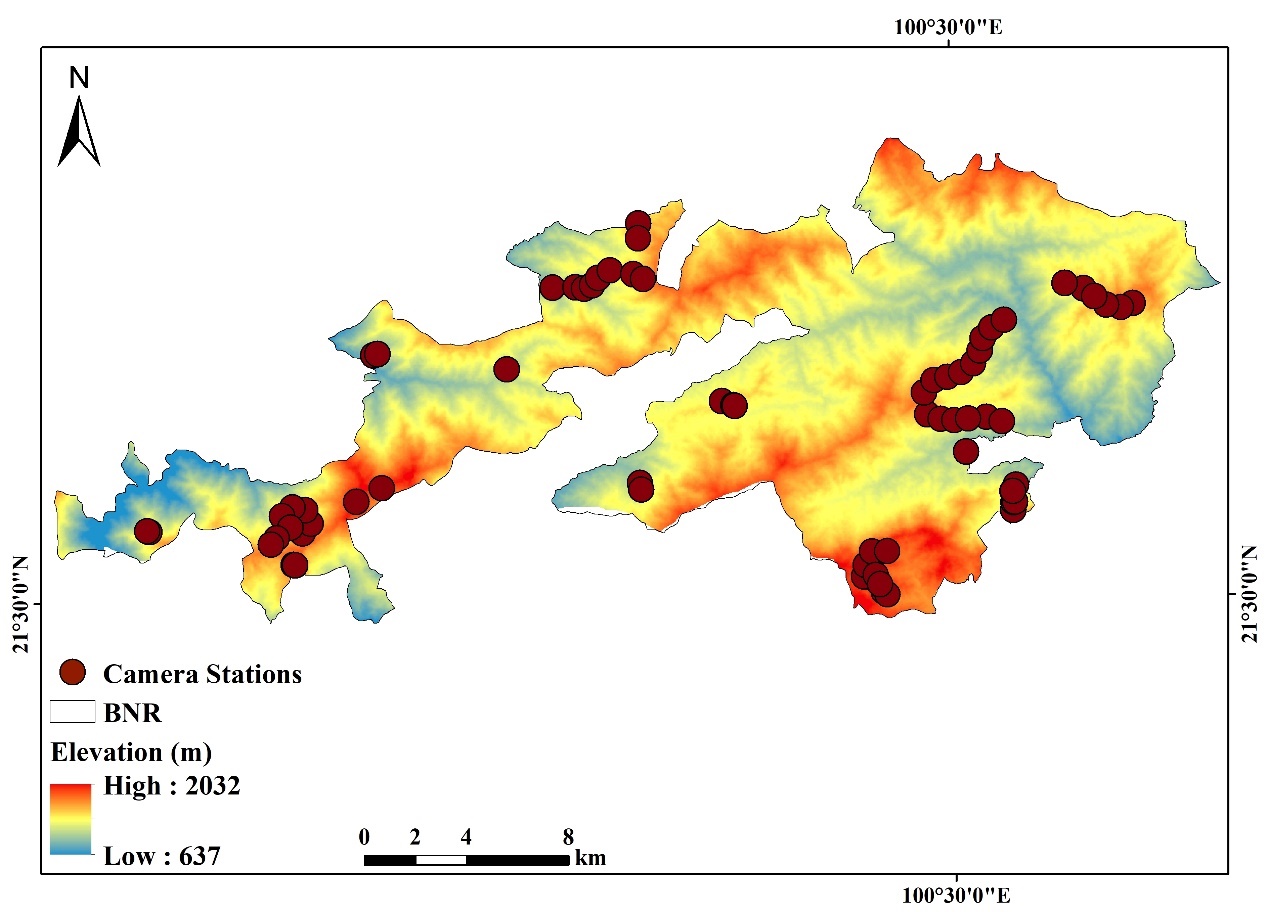


Fig. S3. The elevation range of BNR. BNR represents Bulong Prefectural Nature Reserve.


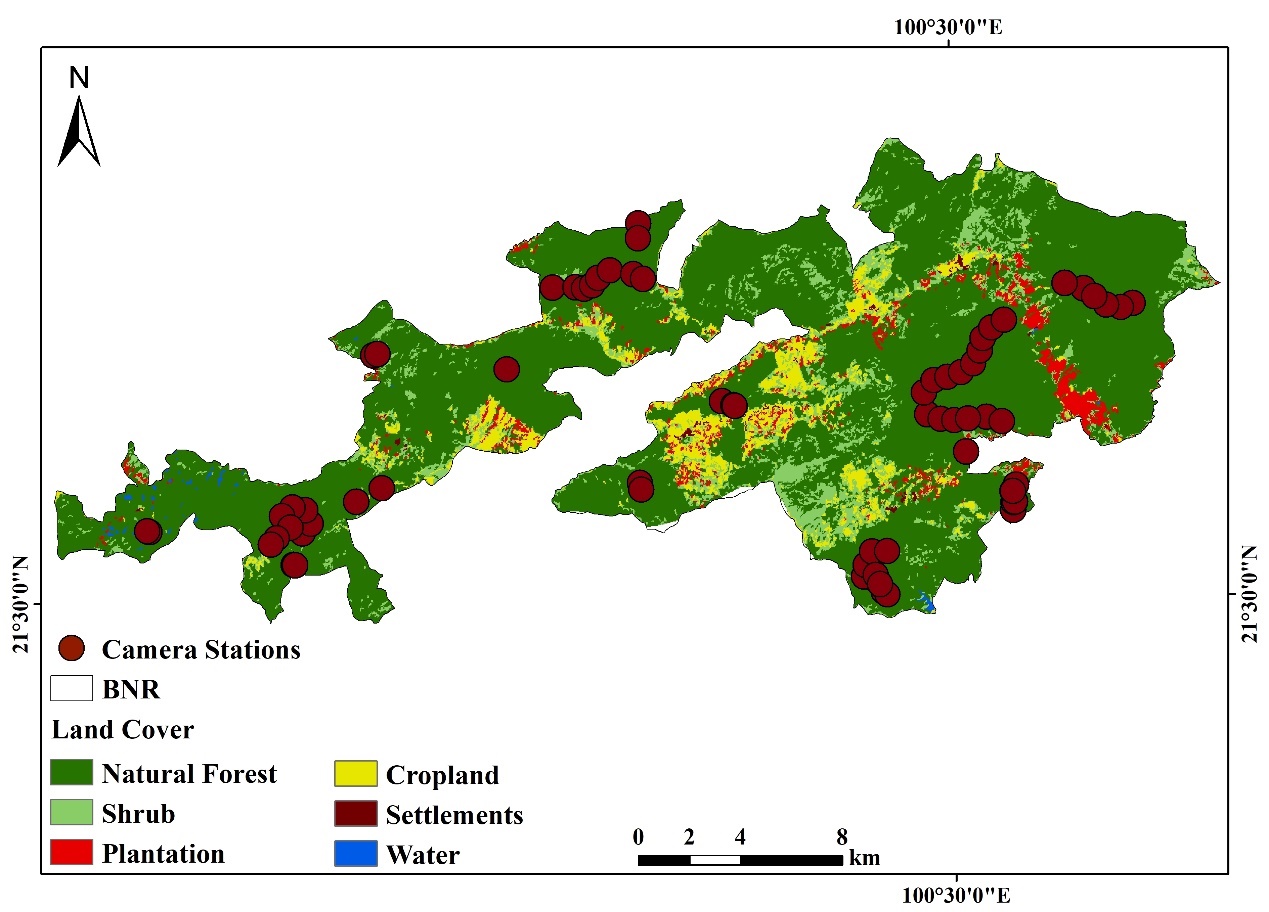


Fig. S4. The land use types of BNR. BNR represents Bulong Prefectural Nature Reserve.


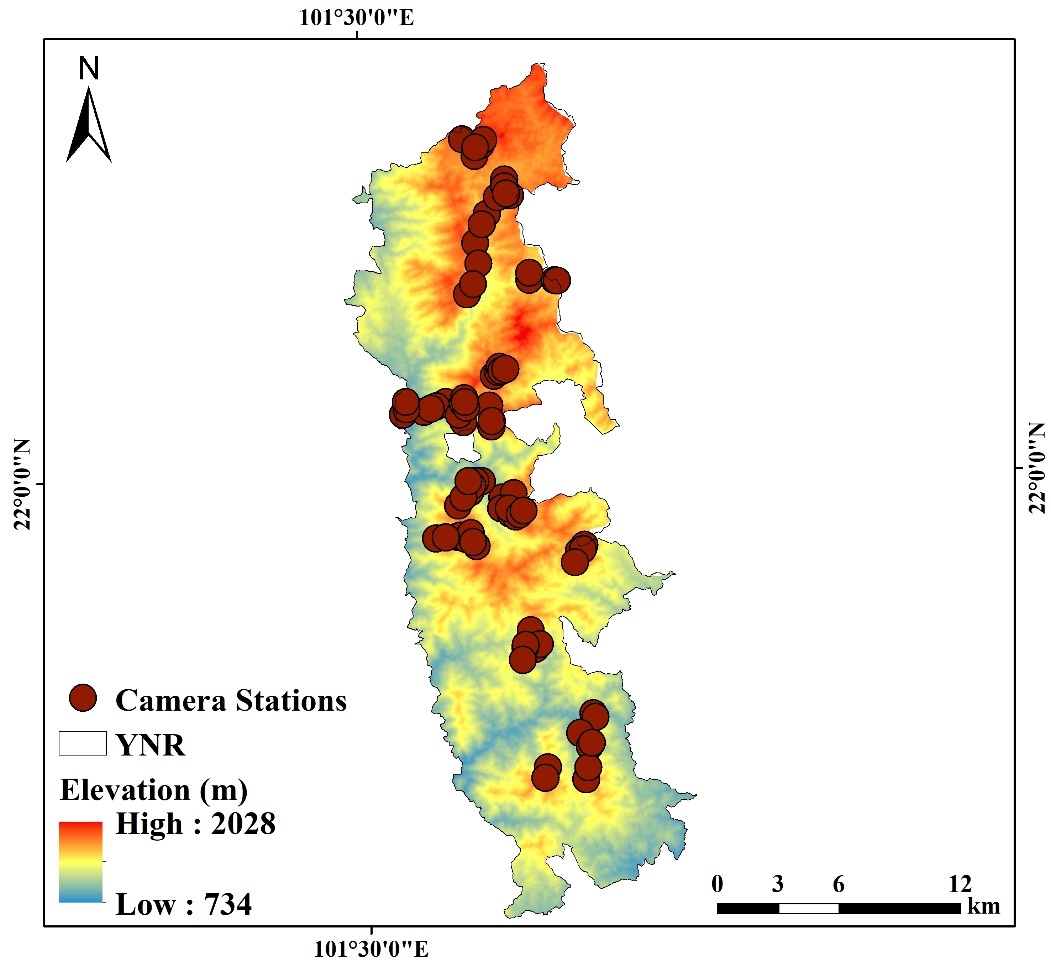


Fig. S5. The elevation range of YNR. YNR represents Yiwu Prefectural Nature Reserve.


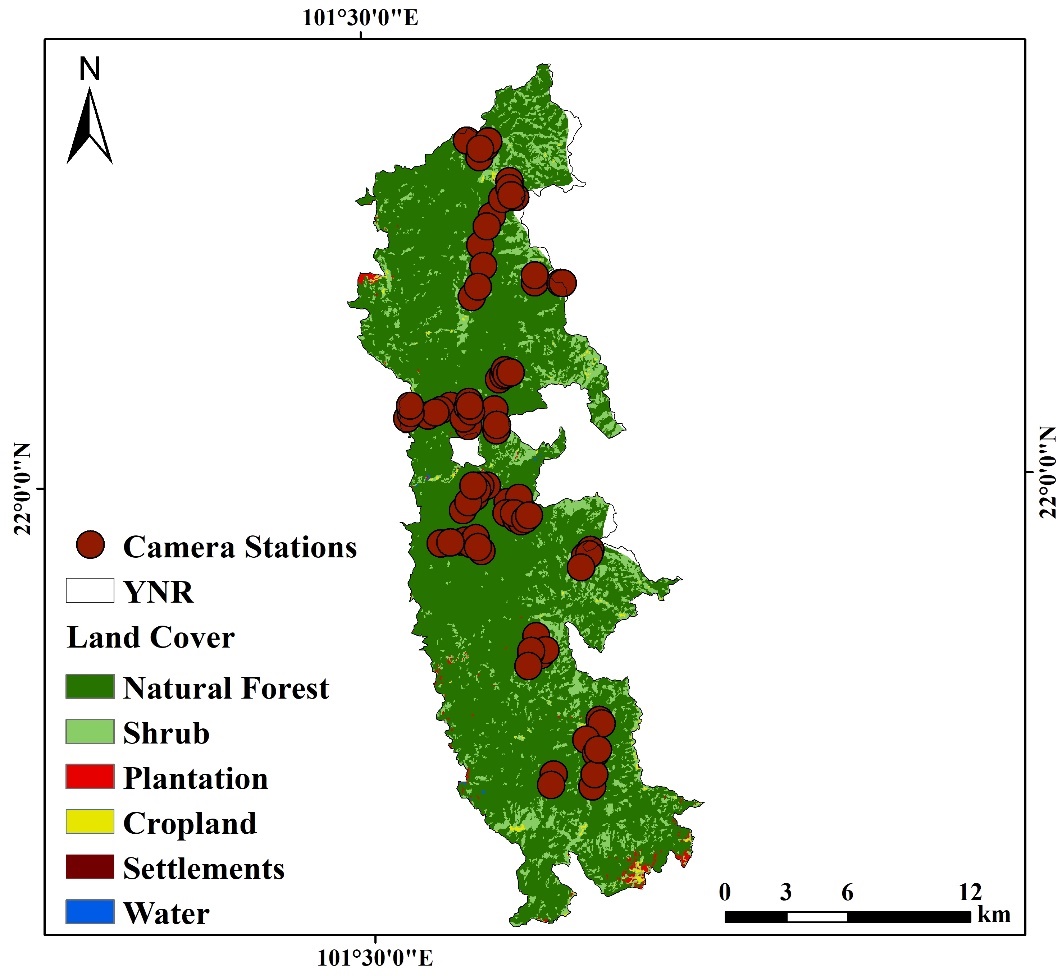


Fig. S6. The land use types of YNR. YNR represents Yiwu Prefectural Nature Reserve.


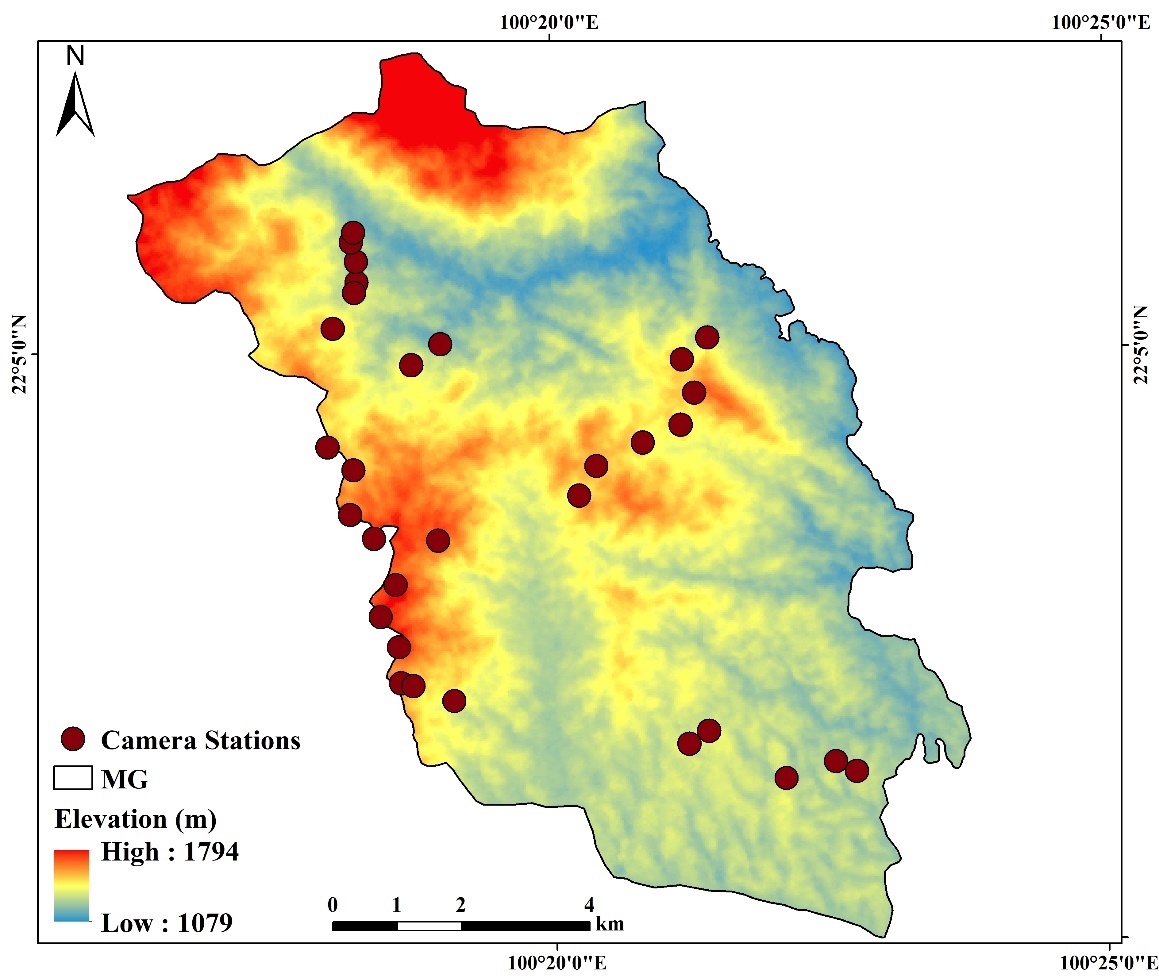


Fig. S7. The elevation range of MG. MG represents ManGao sub-reserve.


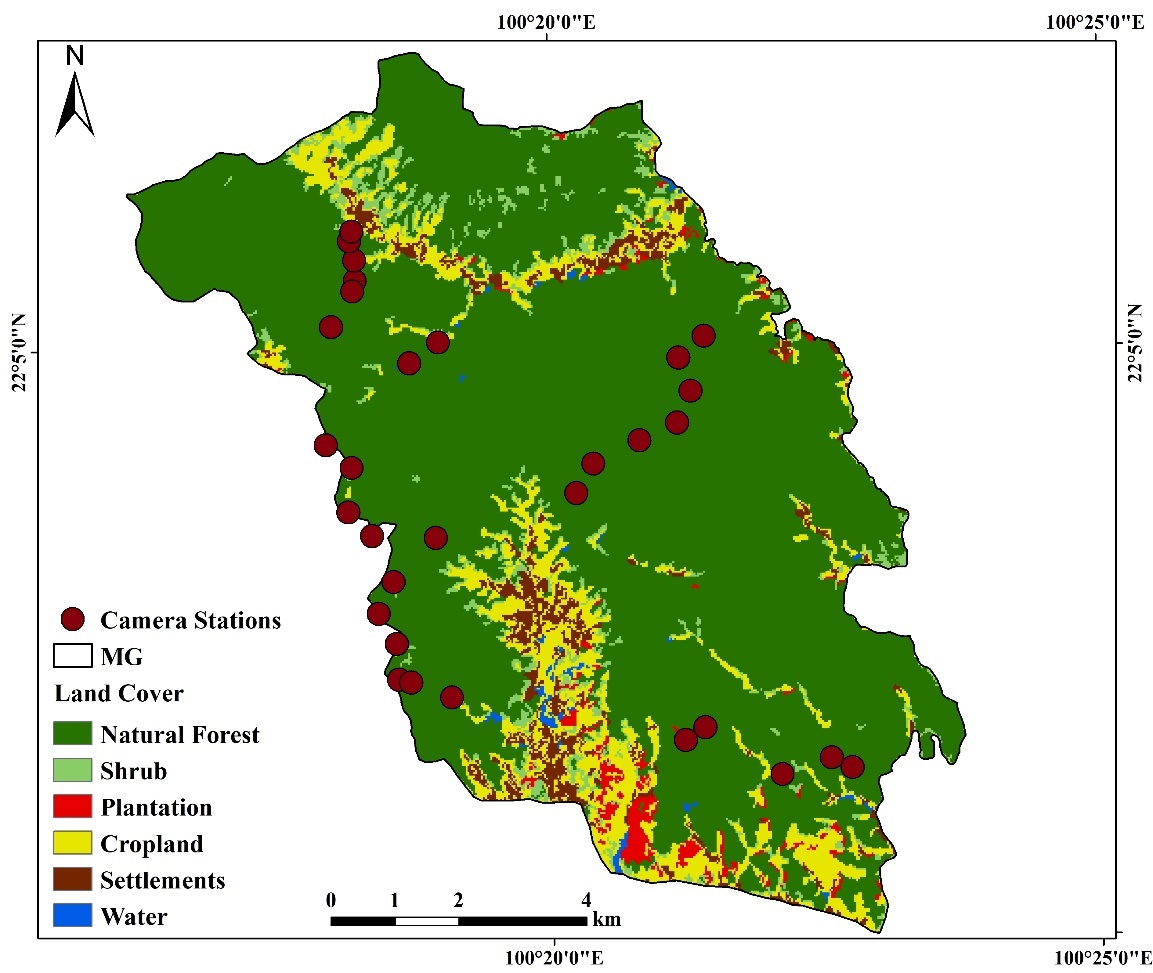


Fig. S8. The land use types of MG. MG represents ManGao sub-reserve.


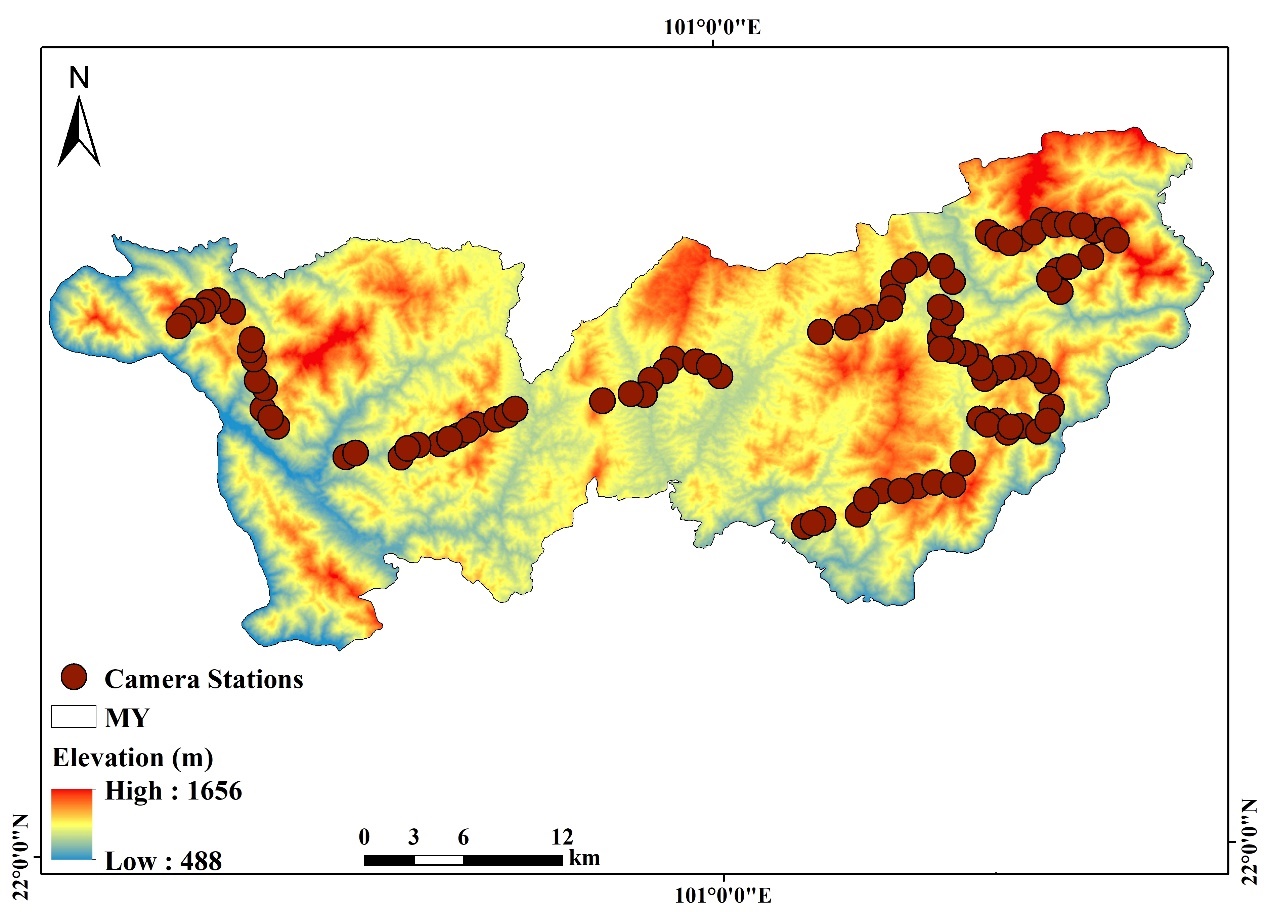


Fig. S9. The elevation range of MY. MY represents MengYang sub-reserve.


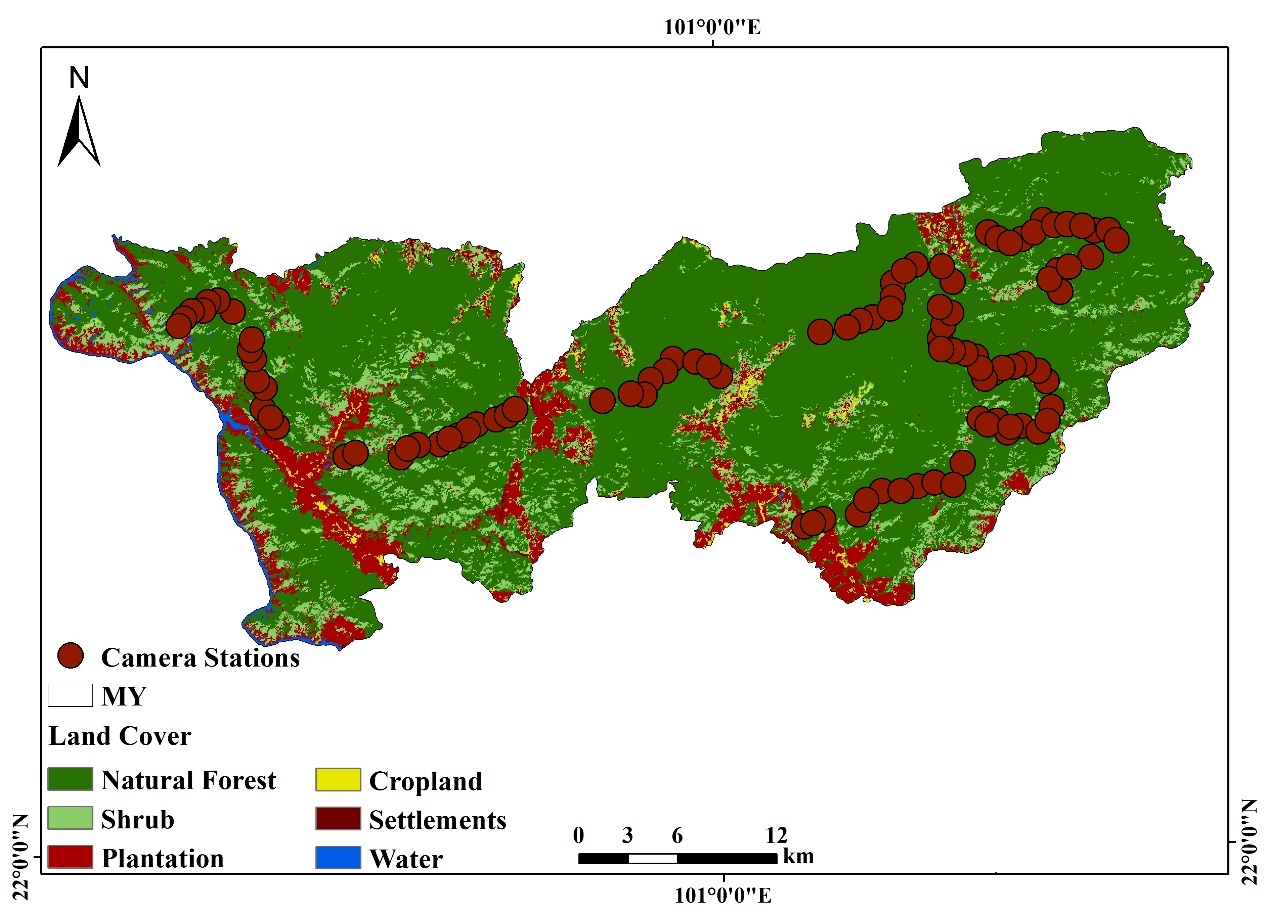


Fig. S10. The land use types of MY. MY represents MengYang sub-reserve.


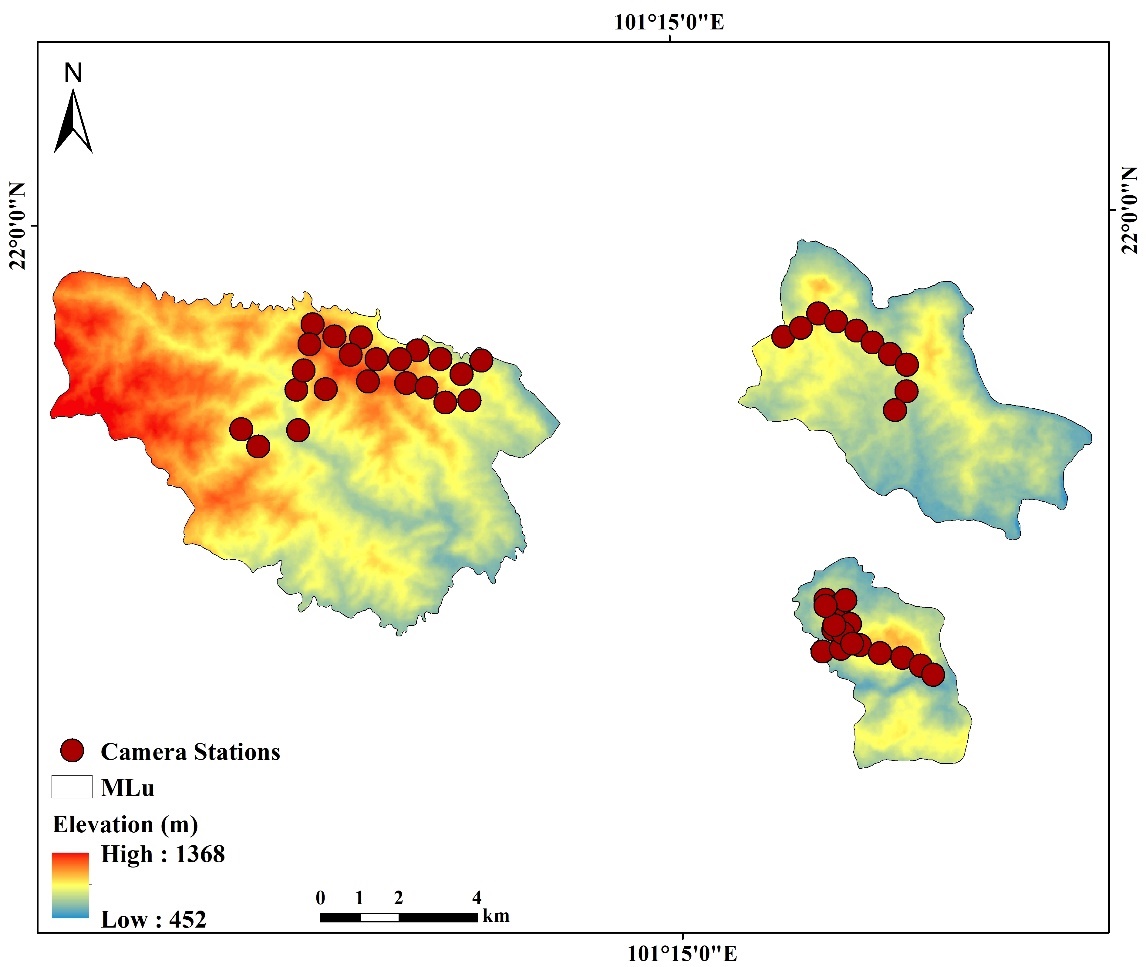


Fig. S11. The elevation range of MLu. MLu represents MengLun sub-reserve.


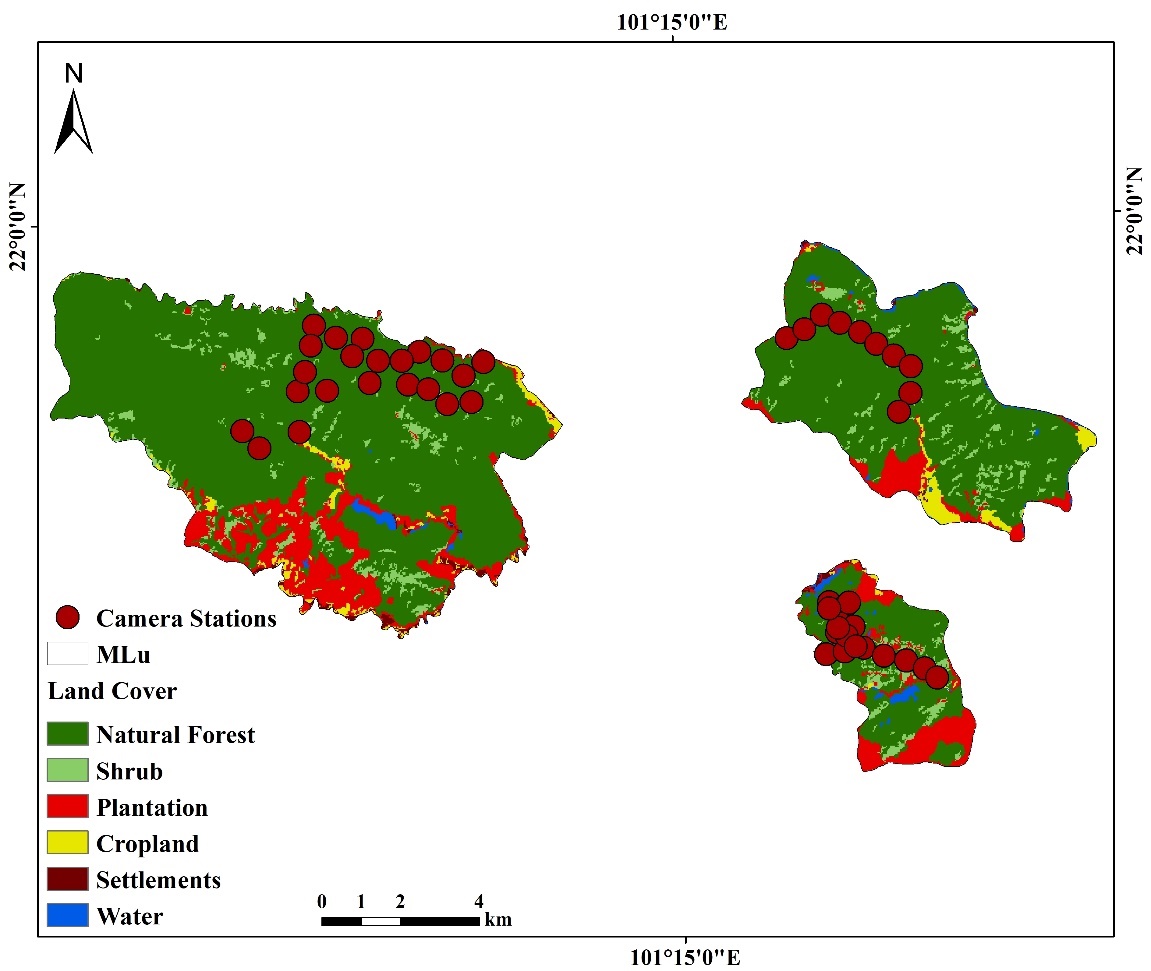


Fig. S12. The land use types of MLu. MLu represents MengLun sub-reserve.


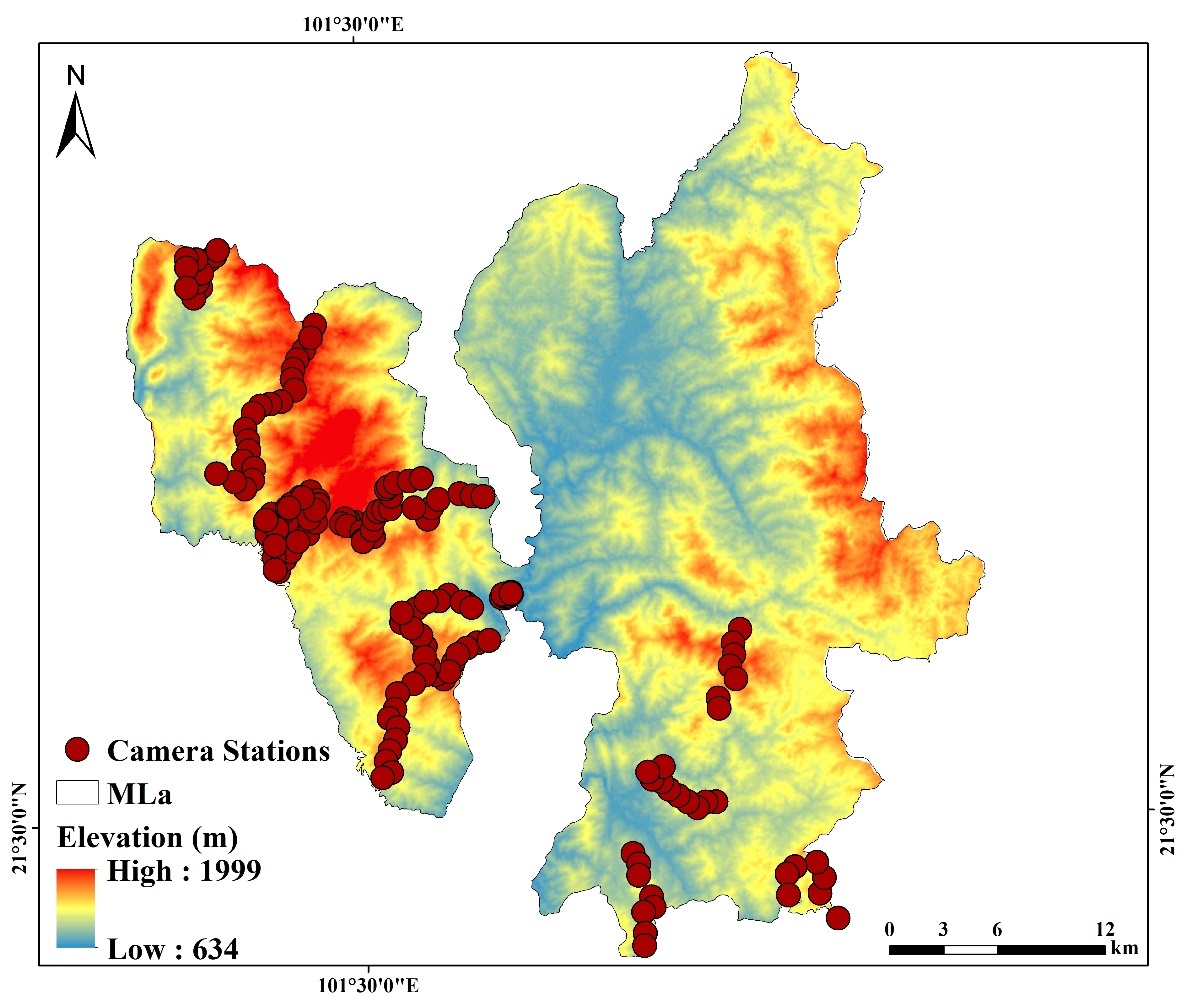


Fig. S13. The elevation range of MLa. MLa represents MengLa sub-reserve.


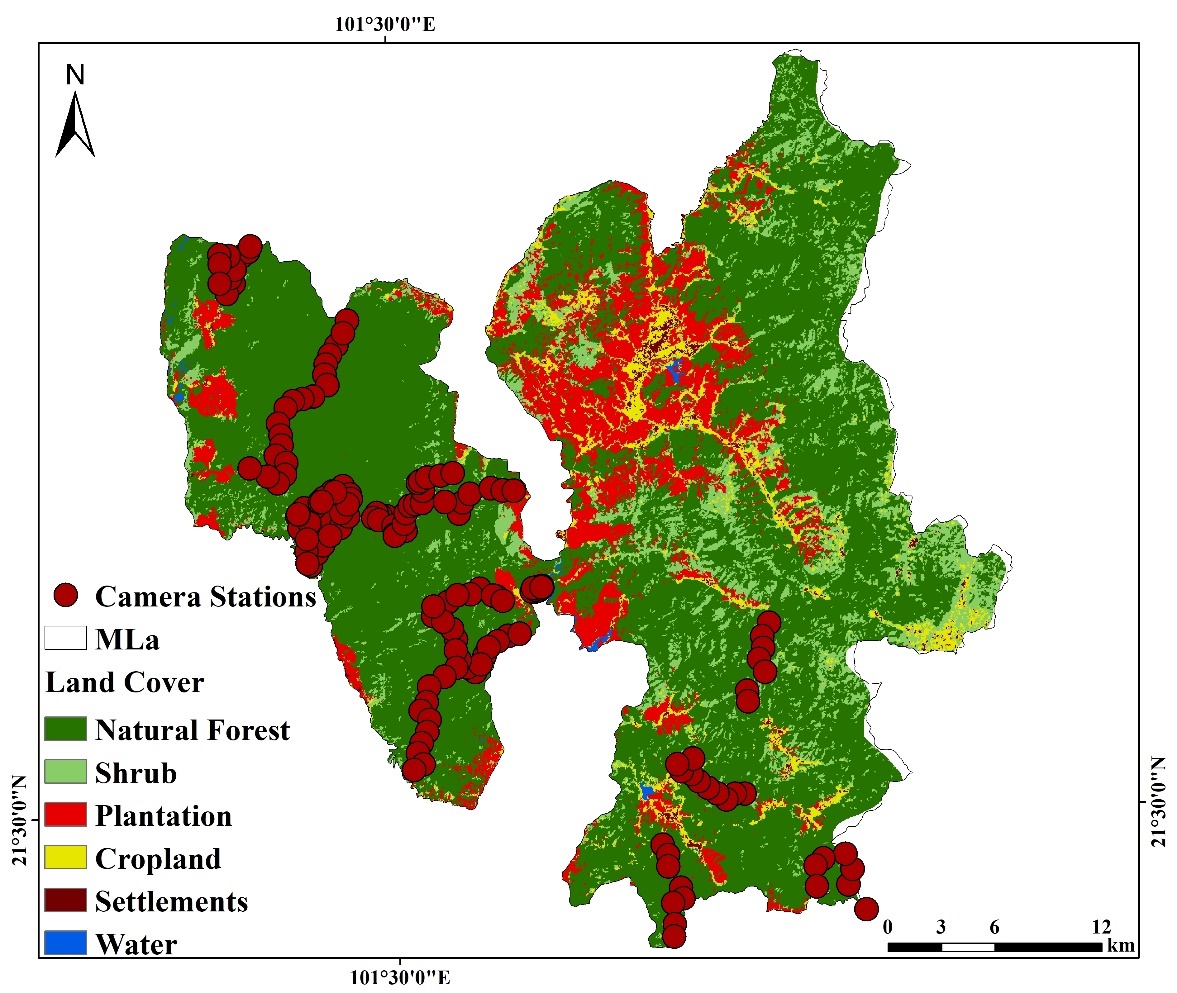


Fig. S14. The land use types of MLa. MLa represents MengLa sub-reserve.


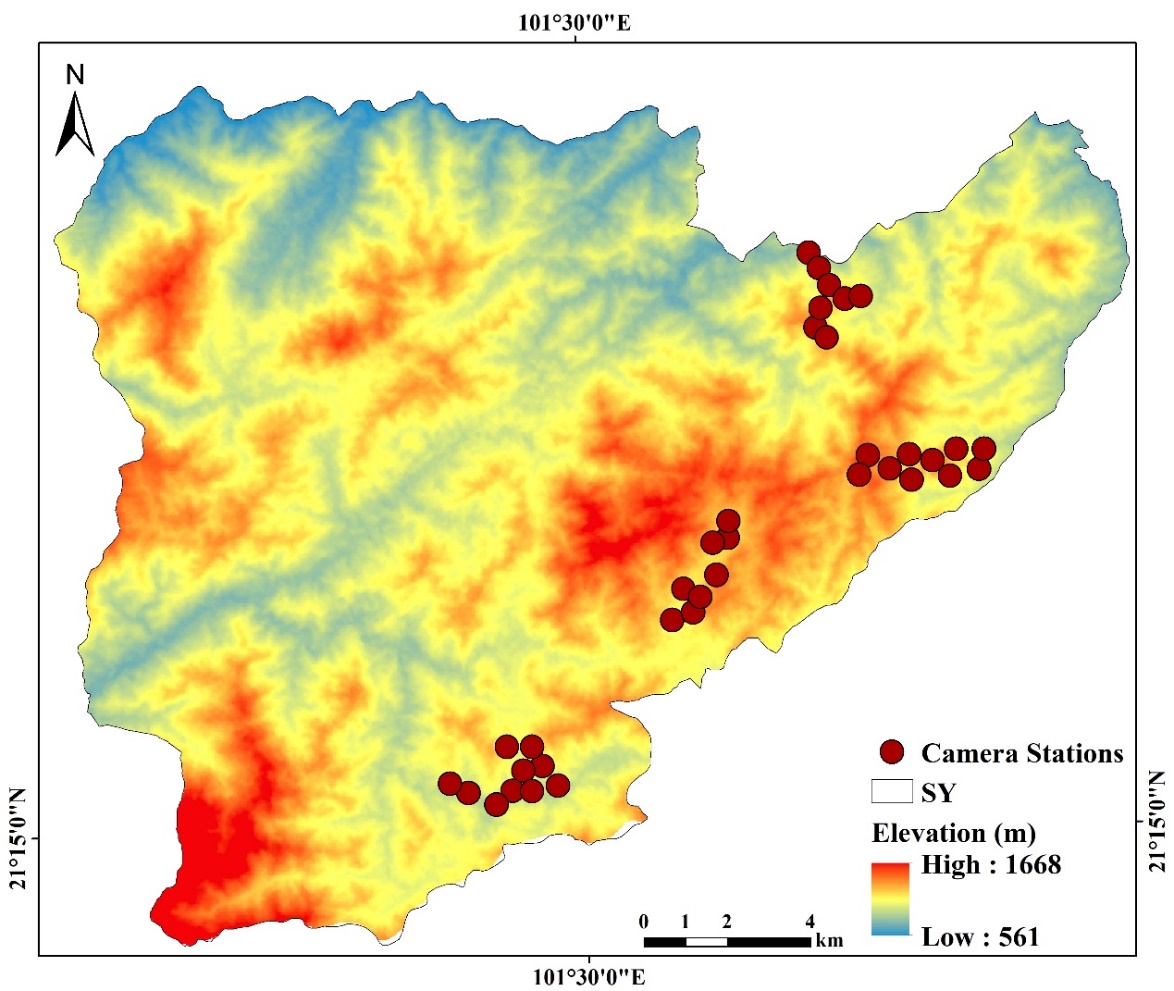


Fig. S15. The elevation range of SY. SY represents ShangYong sub-reserve.


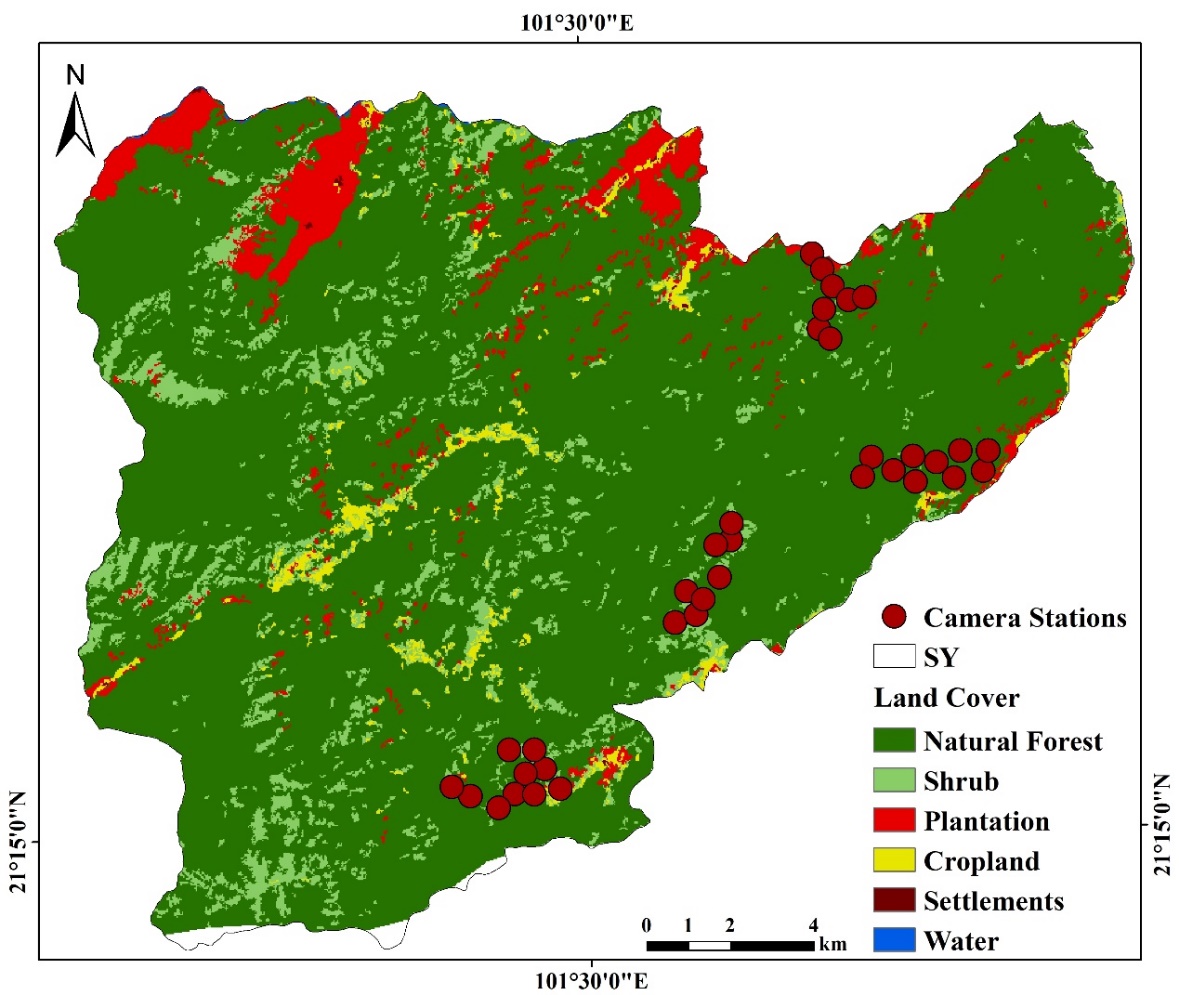


Fig. S16. The land use types of SY. SY represents ShangYong sub-reserve.


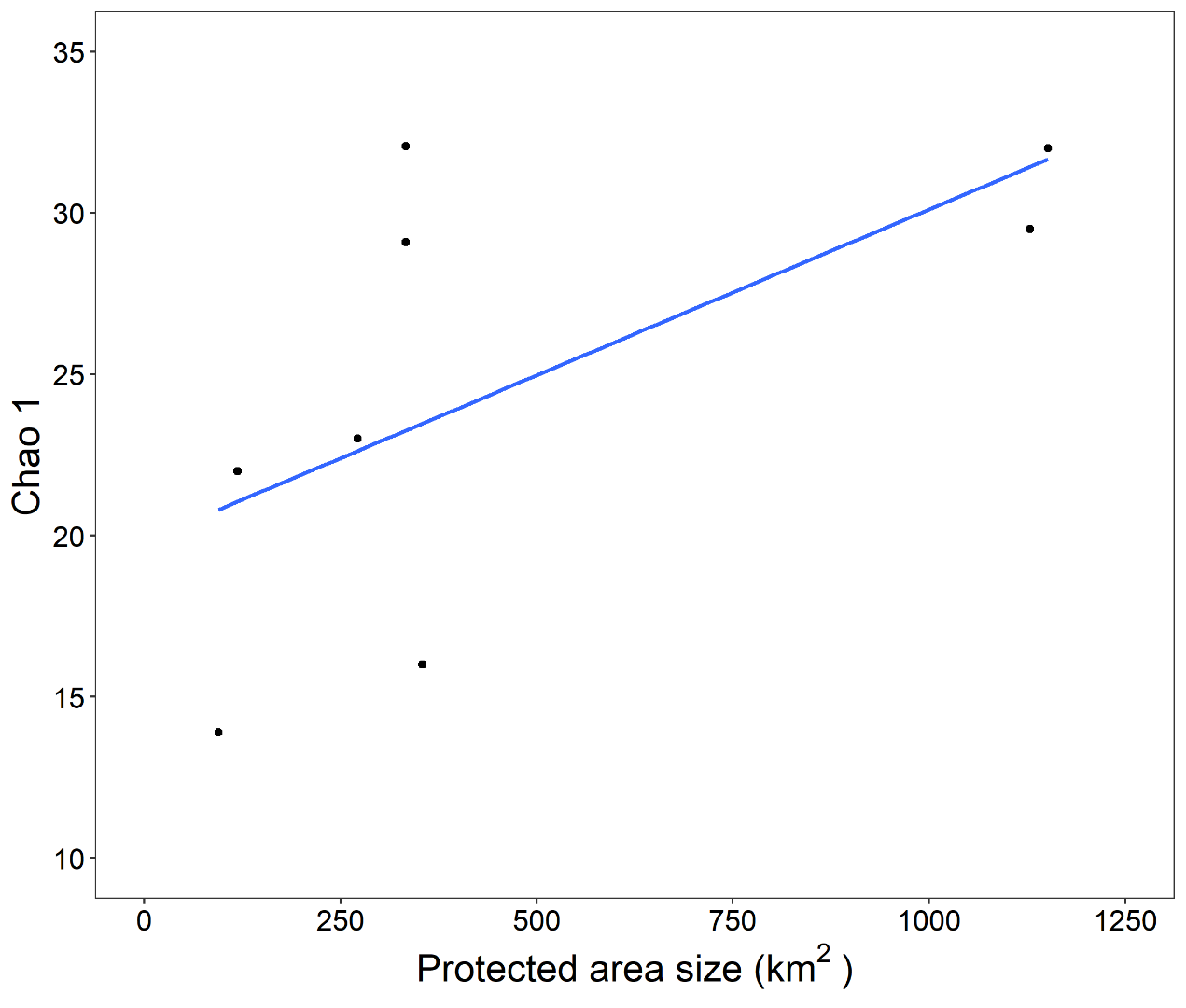


Fig. S17. The relationship between protected area size and estimated species richness chao 1


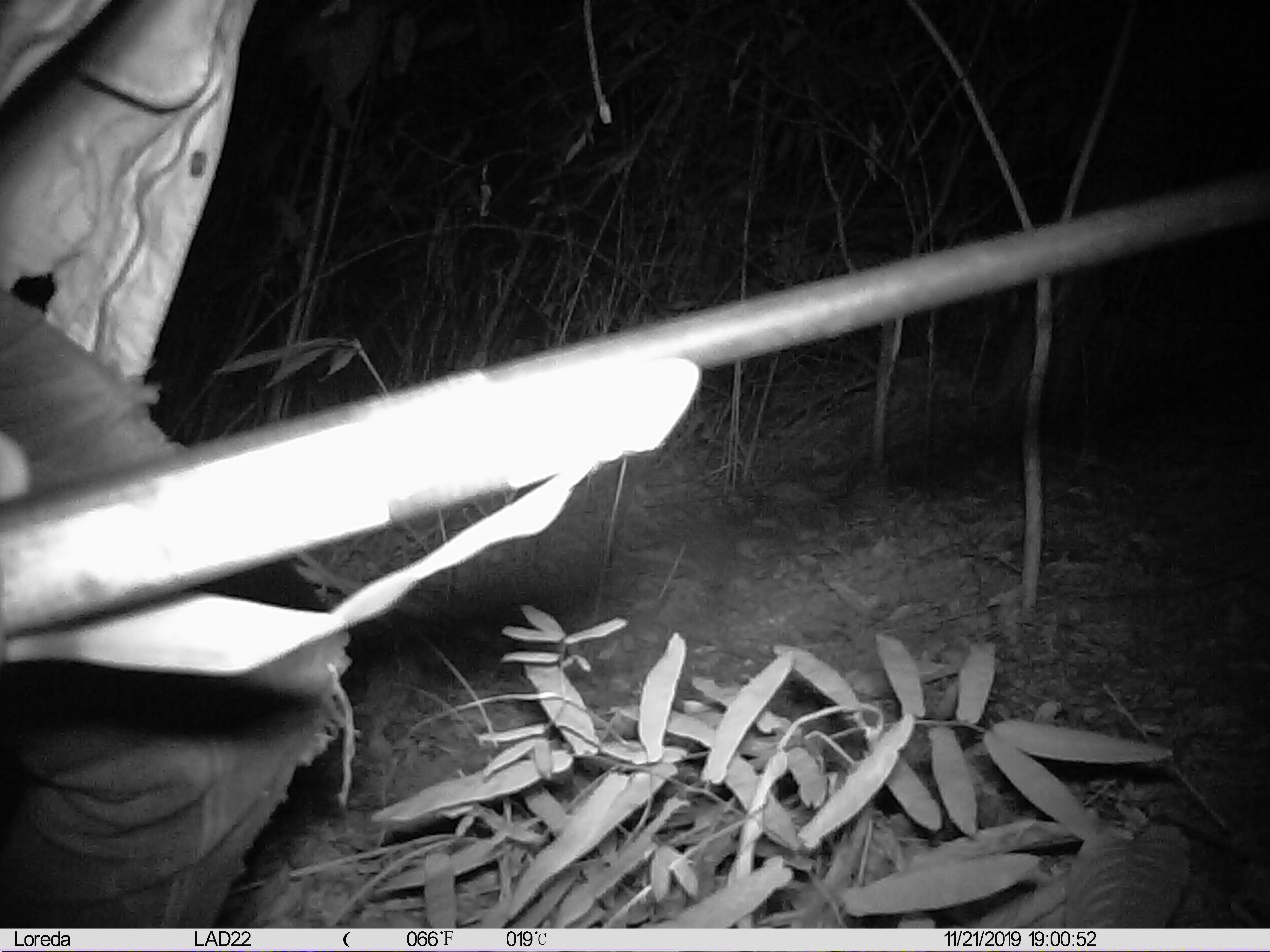


Fig. S18. An armed hunter from camera-trap surveys


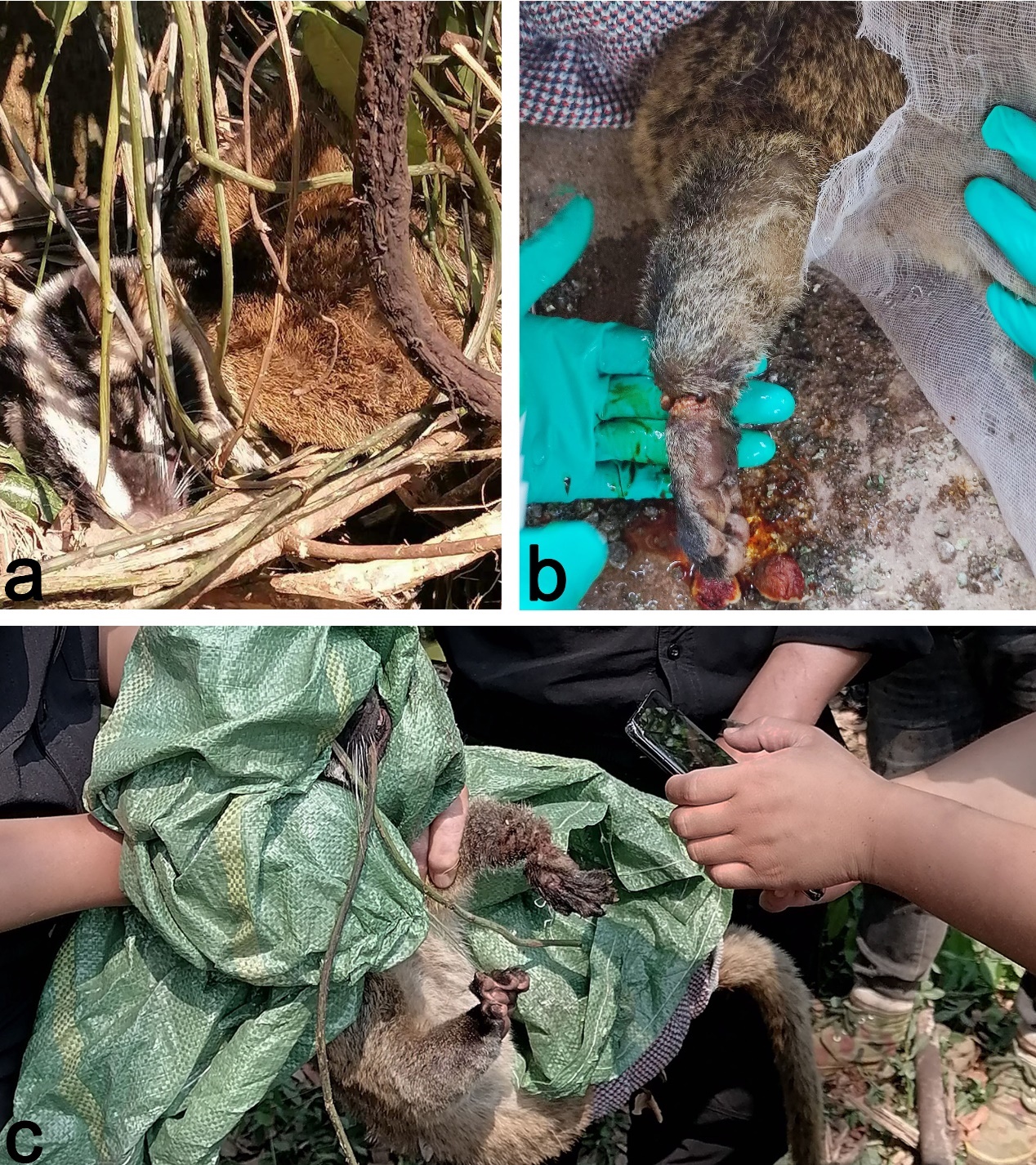


Fig. S19. A *P. larvata* caught in a trap

**Data**

| Camera Site | Position^1^ | Reserve^2^ | Obs | Jack1ab | Jack1abP | Jack2ab | Jack2abP | Chao1 | Chao1P |
| --- | --- | --- | --- | --- | --- | --- | --- | --- | --- |
| MG002 | W | MG | 2 | 2 | 2 | 2 | 2 | 2 | 2 |
| MG003 | W | MG | 3 | 3 | 3 | 3 | 3 | 3 | 3 |
| MG006 | W | MG | 4 | 4 | 4 | 4 | 4 | 4 | 4 |
| MG007 | W | MG | 3 | 3 | 3 | 3 | 3 | 3 | 3 |
| MG008 | W | MG | 4 | 4 | 4 | 4 | 4 | 4 | 4 |
| MG009 | W | MG | 5 | 5 | 5 | 5 | 5 | 5 | 5 |
| MG010 | W | MG | 3 | 3 | 3 | 3 | 3 | 3 | 3 |
| MG011 | W | MG | 1 | 1 | 1 | 1 | 1 | 1 | 1 |
| MG012 | W | MG | 8 | 8 | 8 | 8 | 8 | 8 | 8 |
| MG017 | W | MG | 4 | 4 | 4 | 4 | 4 | 4 | 4 |
| MG019 | W | MG | 1 | 1 | 1 | 1 | 1 | 1 | 1 |
| MG020 | W | MG | 1 | 1 | 1 | 1 | 1 | 1 | 1 |
| MG022 | W | MG | 1 | 1 | 1 | 1 | 1 | 1 | 1 |
| MG023 | W | MG | 3 | 3 | 3 | 3 | 3 | 3 | 3 |
| MG024 | W | MG | 3 | 3 | 3 | 3 | 3 | 3 | 3 |
| MG025 | W | MG | 3 | 3 | 3 | 3 | 3 | 3 | 3 |
| MG027 | W | MG | 4 | 4 | 4 | 4 | 4 | 4 | 4 |
| MG028 | W | MG | 2 | 2 | 2 | 2 | 2 | 2 | 2 |
| MG029 | W | MG | 1 | 1 | 1 | 1 | 1 | 1 | 1 |
| MG030 | W | MG | 1 | 1 | 1 | 1 | 1 | 1 | 1 |
| MG032 | W | MG | 2 | 2 | 2 | 2 | 2 | 2 | 2 |
| MG035 | W | MG | 1 | 1 | 1 | 1 | 1 | 1 | 1 |
| MG037 | W | MG | 2 | 2 | 2 | 2 | 2 | 2 | 2 |
| MG039 | W | MG | 1 | 1 | 1 | 1 | 1 | 1 | 1 |
| MG040 | W | MG | 1 | 1 | 1 | 1 | 1 | 1 | 1 |
| MG041 | W | MG | 3 | 3 | 3 | 3 | 3 | 3 | 3 |
| MG044 | W | MG | 2 | 2 | 2 | 2 | 2 | 2 | 2 |
| MG045 | W | MG | 5 | 5 | 5 | 5 | 5 | 5 | 5 |
| BL001 | W | BNR | 7 | 7 | 7 | 7 | 7 | 7 | 7 |
| BL002 | W | BNR | 5 | 5 | 5 | 5 | 5 | 5 | 5 |
| BL003 | W | BNR | 5 | 5 | 5 | 5 | 5 | 5 | 5 |
| BL004 | W | BNR | 5 | 5 | 5 | 5 | 5 | 5 | 5 |
| BL005 | W | BNR | 7 | 7 | 7 | 7 | 7 | 7 | 7 |
| BL006 | W | BNR | 8 | 8 | 8 | 8 | 8 | 8 | 8 |
| BL008 | W | BNR | 6 | 6 | 6 | 6 | 6 | 6 | 6 |
| BL009 | W | BNR | 6 | 6 | 6 | 6 | 6 | 6 | 6 |
| BL010 | W | BNR | 3 | 3 | 3 | 3 | 3 | 3 | 3 |
| BL011 | W | BNR | 5 | 5 | 5 | 5 | 5 | 5 | 5 |
| BL012 | W | BNR | 5 | 5 | 5 | 5 | 5 | 5 | 5 |
| BL013 | W | BNR | 4 | 4 | 4 | 4 | 4 | 4 | 4 |
| BL014 | W | BNR | 5 | 5 | 5 | 5 | 5 | 5 | 5 |
| BL015 | W | BNR | 6 | 6 | 6 | 6 | 6 | 6 | 6 |
| BL016 | W | BNR | 7 | 7 | 7 | 7 | 7 | 7 | 7 |
| BL017 | W | BNR | 6 | 6 | 6 | 6 | 6 | 6 | 6 |
| BL018 | W | BNR | 5 | 5 | 5 | 5 | 5 | 5 | 5 |
| BL019 | W | BNR | 6 | 6 | 6 | 6 | 6 | 6 | 6 |
| BL020 | W | BNR | 5 | 5 | 5 | 5 | 5 | 5 | 5 |
| BL021 | W | BNR | 5 | 5 | 5 | 5 | 5 | 5 | 5 |
| BL022 | W | BNR | 4 | 4 | 4 | 4 | 4 | 4 | 4 |
| BL023 | W | BNR | 5 | 5 | 5 | 5 | 5 | 5 | 5 |
| BL024 | W | BNR | 6 | 6 | 6 | 6 | 6 | 6 | 6 |
| BL025 | W | BNR | 3 | 3 | 3 | 3 | 3 | 3 | 3 |
| BL026 | W | BNR | 6 | 6 | 6 | 6 | 6 | 6 | 6 |
| BL027 | W | BNR | 6 | 6 | 6 | 6 | 6 | 6 | 6 |
| BL028 | W | BNR | 5 | 5 | 5 | 5 | 5 | 5 | 5 |
| BL029 | W | BNR | 5 | 5 | 5 | 5 | 5 | 5 | 5 |
| BL030 | W | BNR | 3 | 3 | 3 | 3 | 3 | 3 | 3 |
| BL032 | W | BNR | 1 | 1 | 1 | 1 | 1 | 1 | 1 |
| BL033 | W | BNR | 3 | 3 | 3 | 3 | 3 | 3 | 3 |
| BL034 | W | BNR | 3 | 3 | 3 | 3 | 3 | 3 | 3 |
| BL035 | W | BNR | 3 | 3 | 3 | 3 | 3 | 3 | 3 |
| BL036 | W | BNR | 1 | 1 | 1 | 1 | 1 | 1 | 1 |
| BL037 | W | BNR | 3 | 3 | 3 | 3 | 3 | 3 | 3 |
| BL038 | W | BNR | 1 | 1 | 1 | 1 | 1 | 1 | 1 |
| BL040 | W | BNR | 2 | 2 | 2 | 2 | 2 | 2 | 2 |
| BL042 | W | BNR | 6 | 6 | 6 | 6 | 6 | 6 | 6 |
| BL043 | W | BNR | 5 | 5 | 5 | 5 | 5 | 5 | 5 |
| BL044 | W | BNR | 3 | 3 | 3 | 3 | 3 | 3 | 3 |
| BL045 | W | BNR | 2 | 2 | 2 | 2 | 2 | 2 | 2 |
| BL046 | W | BNR | 4 | 4 | 4 | 4 | 4 | 4 | 4 |
| BLEX001 | W | BNR | 2 | 2 | 2 | 2 | 2 | 2 | 2 |
| BLEX002 | W | BNR | 5 | 5 | 5 | 5 | 5 | 5 | 5 |
| BLEX003 | W | BNR | 6 | 6 | 6 | 6 | 6 | 6 | 6 |
| BL1-2 | W | BNR | 1 | 1 | 1 | 1 | 1 | 1 | 1 |
| BL1-4 | W | BNR | 1 | 1 | 1 | 0 | 0 | 1 | 1 |
| BL1-5 | W | BNR | 3 | 3 | 3 | 3 | 3 | 3 | 3 |
| BL2-1 | W | BNR | 4 | 4 | 4 | 4 | 4 | 4 | 4 |
| BL2-2 | W | BNR | 3 | 3 | 3 | 3 | 3 | 3 | 3 |
| BL2-3 | W | BNR | 4 | 4 | 4 | 4 | 4 | 4 | 4 |
| BL2-4 | W | BNR | 5 | 5 | 5 | 5 | 5 | 5 | 5 |
| BL2-5 | W | BNR | 5 | 5 | 5 | 5 | 5 | 5 | 5 |
| BL2-6 | W | BNR | 5 | 5 | 5 | 5 | 5 | 5 | 5 |
| BL3-4 | W | BNR | 1 | 1 | 1 | 1 | 1 | 1 | 1 |
| BL3-5 | W | BNR | 2 | 2 | 2 | 2 | 2 | 2 | 2 |
| BL3-6 | W | BNR | 2 | 2 | 2 | 2 | 2 | 2 | 2 |
| BL3-7 | W | BNR | 3 | 3 | 3 | 3 | 3 | 3 | 3 |
| BL3-8 | W | BNR | 1 | 1 | 1 | 1 | 1 | 1 | 1 |
| BL3-9 | W | BNR | 1 | 1 | 1 | 1 | 1 | 1 | 1 |
| BL3-10 | W | BNR | 1 | 1 | 1 | 1 | 1 | 1 | 1 |
| BL4-1 | W | BNR | 1 | 1 | 1 | 1 | 1 | 1 | 1 |
| BL4-2 | W | BNR | 3 | 3 | 3 | 3 | 3 | 3 | 3 |
| BL4-5 | W | BNR | 1 | 1 | 1 | 1 | 1 | 1 | 1 |
| BL4-6 | W | BNR | 3 | 3 | 3 | 3 | 3 | 3 | 3 |
| BL5-2 | W | BNR | 3 | 3 | 3 | 3 | 3 | 3 | 3 |
| BL6-1 | W | BNR | 2 | 2 | 2 | 2 | 2 | 2 | 2 |
| BL6-4 | W | BNR | 2 | 2 | 2 | 2 | 2 | 2 | 2 |
| BL7-1 | W | BNR | 6 | 6 | 6 | 6 | 6 | 6 | 6 |
| BL7-3 | W | BNR | 2 | 2 | 2 | 2 | 2 | 2 | 2 |
| BL8-1 | W | BNR | 3 | 3 | 3 | 3 | 3 | 3 | 3 |
| BL8-4 | W | BNR | 3 | 3 | 3 | 3 | 3 | 3 | 3 |
| MLu001 | E | MLu | 9 | 9 | 9 | 9 | 9 | 9 | 9 |
| MLu002 | E | MLu | 10 | 10 | 10 | 10 | 10 | 10 | 10 |
| MLu003 | E | MLu | 12 | 12 | 12 | 12 | 12 | 12 | 12 |
| MLu004 | E | MLu | 11 | 11 | 11 | 11 | 11 | 11 | 11 |
| MLu005 | E | MLu | 10 | 10 | 10 | 10 | 10 | 10 | 10 |
| MLu006 | E | MLu | 11 | 11 | 11 | 11 | 11 | 11 | 11 |
| MLu007 | E | MLu | 11 | 11 | 11 | 11 | 11 | 11 | 11 |
| MLu008 | E | MLu | 12 | 12 | 12 | 12 | 12 | 12 | 12 |
| MLu009 | E | MLu | 8 | 8 | 8 | 8 | 8 | 8 | 8 |
| MLu010 | E | MLu | 10 | 10 | 10 | 10 | 10 | 10 | 10 |
| MLu011 | E | MLu | 13 | 13 | 13 | 13 | 13 | 13 | 13 |
| MLu012 | E | MLu | 14 | 14 | 14 | 14 | 14 | 14 | 14 |
| MLu013 | E | MLu | 9 | 9 | 9 | 9 | 9 | 9 | 9 |
| MLu014 | E | MLu | 9 | 9 | 9 | 9 | 9 | 9 | 9 |
| MLu015 | E | MLu | 6 | 6 | 6 | 6 | 6 | 6 | 6 |
| MLu016 | E | MLu | 5 | 5 | 5 | 5 | 5 | 5 | 5 |
| MLu017 | E | MLu | 9 | 9 | 9 | 9 | 9 | 9 | 9 |
| MLu018 | E | MLu | 7 | 7 | 7 | 7 | 7 | 7 | 7 |
| MLu019 | E | MLu | 11 | 11 | 11 | 11 | 11 | 11 | 11 |
| MLu020 | E | MLu | 7 | 7 | 7 | 7 | 7 | 7 | 7 |
| MLu017_2017 | E | MLu | 3 | 3 | 3 | 3 | 3 | 3 | 3 |
| MLu018_2017 | E | MLu | 3 | 3 | 3 | 3 | 3 | 3 | 3 |
| MLu021 | E | MLu | 8 | 8 | 8 | 8 | 8 | 8 | 8 |
| MLu022 | E | MLu | 7 | 7 | 7 | 7 | 7 | 7 | 7 |
| MLu023 | E | MLu | 7 | 7 | 7 | 7 | 7 | 7 | 7 |
| MLu024 | E | MLu | 9 | 9 | 9 | 9 | 9 | 9 | 9 |
| MLu025 | E | MLu | 9 | 9 | 9 | 9 | 9 | 9 | 9 |
| MLu026 | E | MLu | 7 | 7 | 7 | 7 | 7 | 7 | 7 |
| MLu027 | E | MLu | 6 | 6 | 6 | 6 | 6 | 6 | 6 |
| MLu028 | E | MLu | 8 | 8 | 8 | 8 | 8 | 8 | 8 |
| MLu029 | E | MLu | 9 | 9 | 9 | 9 | 9 | 9 | 9 |
| MLu030 | E | MLu | 9 | 9 | 9 | 9 | 9 | 9 | 9 |
| MLu031 | E | MLu | 9 | 9 | 9 | 9 | 9 | 9 | 9 |
| MLu032 | E | MLu | 4 | 4 | 4 | 4 | 4 | 4 | 4 |
| MLu0321 | E | MLu | 8 | 8 | 8 | 8 | 8 | 8 | 8 |
| MLu033 | E | MLu | 7 | 7 | 7 | 7 | 7 | 7 | 7 |
| MLu034 | E | MLu | 8 | 8 | 8 | 8 | 8 | 8 | 8 |
| MLu035 | E | MLu | 6 | 6 | 6 | 6 | 6 | 6 | 6 |
| MLu036 | E | MLu | 7 | 7 | 7 | 7 | 7 | 7 | 7 |
| MLu037 | E | MLu | 8 | 8 | 8 | 8 | 8 | 8 | 8 |
| MLu038 | E | MLu | 6 | 6 | 6 | 6 | 6 | 6 | 6 |
| MLu039 | E | MLu | 7 | 7 | 7 | 7 | 7 | 7 | 7 |
| MLu040 | E | MLu | 6 | 6 | 6 | 6 | 6 | 6 | 6 |
| MLu041 | E | MLu | 9 | 9 | 9 | 9 | 9 | 9 | 9 |
| LSL1004 | E | MLu | 1 | 1 | 1 | 1 | 1 | 1 | 1 |
| LSL1005 | E | MLu | 1 | 1 | 1 | 1 | 1 | 1 | 1 |
| LSL1006 | E | MLu | 4 | 4 | 4 | 4 | 4 | 4 | 4 |
| LSL2002 | E | MLu | 2 | 2 | 2 | 2 | 2 | 2 | 2 |
| LSL2005 | E | MLu | 1 | 1 | 1 | 1 | 1 | 1 | 1 |
| LSL2006 | E | MLu | 1 | 1 | 1 | 1 | 1 | 1 | 1 |
| SY001 | E | SY | 10 | 10 | 10 | 10 | 10 | 10 | 10 |
| SY002 | E | SY | 16 | 16 | 16 | 16 | 16 | 16 | 16 |
| SY003 | E | SY | 10 | 10 | 10 | 10 | 10 | 10 | 10 |
| SY004 | E | SY | 22 | 22 | 22 | 22 | 22 | 22 | 22 |
| SY005 | E | SY | 12 | 12 | 12 | 12 | 12 | 12 | 12 |
| SY006 | E | SY | 12 | 12 | 12 | 12 | 12 | 12 | 12 |
| SY007 | E | SY | 15 | 15 | 15 | 15 | 15 | 15 | 15 |
| SY008 | E | SY | 9 | 9 | 9 | 9 | 9 | 9 | 9 |
| SY009 | E | SY | 12 | 12 | 12 | 12 | 12 | 12 | 12 |
| SY010 | E | SY | 15 | 15 | 15 | 15 | 15 | 15 | 15 |
| SY012 | E | SY | 8 | 8 | 8 | 8 | 8 | 8 | 8 |
| SY013 | E | SY | 5 | 5 | 5 | 5 | 5 | 5 | 5 |
| SY014 | E | SY | 6 | 6 | 6 | 6 | 6 | 6 | 6 |
| SY015 | E | SY | 9 | 9 | 9 | 9 | 9 | 9 | 9 |
| SY016 | E | SY | 6 | 6 | 6 | 6 | 6 | 6 | 6 |
| SY017 | E | SY | 7 | 7 | 7 | 7 | 7 | 7 | 7 |
| SY019 | E | SY | 7 | 7 | 7 | 7 | 7 | 7 | 7 |
| SY020 | E | SY | 13 | 13 | 13 | 13 | 13 | 13 | 13 |
| SY021 | E | SY | 5 | 5 | 5 | 5 | 5 | 5 | 5 |
| SY022 | E | SY | 8 | 8 | 8 | 8 | 8 | 8 | 8 |
| SY023 | E | SY | 6 | 6 | 6 | 6 | 6 | 6 | 6 |
| SY024 | E | SY | 8 | 8 | 8 | 8 | 8 | 8 | 8 |
| SY025 | E | SY | 12 | 12 | 12 | 12 | 12 | 12 | 12 |
| SY026 | E | SY | 13 | 13 | 13 | 13 | 13 | 13 | 13 |
| SY027 | E | SY | 1 | 1 | 1 | 1 | 1 | 1 | 1 |
| SY028 | E | SY | 4 | 4 | 4 | 4 | 4 | 4 | 4 |
| SY029 | E | SY | 10 | 10 | 10 | 10 | 10 | 10 | 10 |
| SY030 | E | SY | 13 | 13 | 13 | 13 | 13 | 13 | 13 |
| SY031 | E | SY | 13 | 13 | 13 | 13 | 13 | 13 | 13 |
| SY032 | E | SY | 8 | 8 | 8 | 8 | 8 | 8 | 8 |
| SY033 | E | SY | 12 | 12 | 12 | 12 | 12 | 12 | 12 |
| SY034 | E | SY | 9 | 9 | 9 | 9 | 9 | 9 | 9 |
| SY035 | E | SY | 10 | 10 | 10 | 10 | 10 | 10 | 10 |
| SY036 | E | SY | 8 | 8 | 8 | 8 | 8 | 8 | 8 |
| SY038 | E | SY | 12 | 12 | 12 | 12 | 12 | 12 | 12 |
| SY039 | E | SY | 9 | 9 | 9 | 9 | 9 | 9 | 9 |
| GYL001 | E | SFF | 8 | 8 | 8 | 8 | 8 | 8 | 8 |
| GYL003 | E | SFF | 7 | 7 | 7 | 7 | 7 | 7 | 7 |
| GYL004 | E | SFF | 7 | 7 | 7 | 7 | 7 | 7 | 7 |
| GYL005 | E | SFF | 5 | 5 | 5 | 5 | 5 | 5 | 5 |
| GYL006 | E | SFF | 6 | 6 | 6 | 6 | 6 | 6 | 6 |
| GYL007 | E | SFF | 3 | 3 | 3 | 3 | 3 | 3 | 3 |
| GYL008 | E | SFF | 6 | 6 | 6 | 6 | 6 | 6 | 6 |
| GYL009 | E | SFF | 4 | 4 | 4 | 4 | 4 | 4 | 4 |
| GYL010 | E | SFF | 8 | 8 | 8 | 8 | 8 | 8 | 8 |
| GYL011 | E | SFF | 9 | 9 | 9 | 9 | 9 | 9 | 9 |
| GYL012 | E | SFF | 3 | 3 | 3 | 3 | 3 | 3 | 3 |
| GYL013 | E | SFF | 5 | 5 | 5 | 5 | 5 | 5 | 5 |
| GYL014 | E | SFF | 4 | 4 | 4 | 4 | 4 | 4 | 4 |
| GYL015 | E | SFF | 5 | 5 | 5 | 5 | 5 | 5 | 5 |
| GYL016 | E | SFF | 6 | 6 | 6 | 6 | 6 | 6 | 6 |
| GYL017 | E | SFF | 3 | 3 | 3 | 3 | 3 | 3 | 3 |
| GYL018 | E | SFF | 4 | 4 | 4 | 4 | 4 | 4 | 4 |
| GYL019 | E | SFF | 2 | 2 | 2 | 2 | 2 | 2 | 2 |
| GYL020 | E | SFF | 2 | 2 | 2 | 2 | 2 | 2 | 2 |
| GYL021 | E | SFF | 6 | 6 | 6 | 6 | 6 | 6 | 6 |
| GYL022 | E | SFF | 3 | 3 | 3 | 3 | 3 | 3 | 3 |
| GYL023 | E | SFF | 5 | 5 | 5 | 5 | 5 | 5 | 5 |
| GYL024 | E | SFF | 4 | 4 | 4 | 4 | 4 | 4 | 4 |
| GYL025 | E | SFF | 1 | 1 | 1 | 1 | 1 | 1 | 1 |
| GYL026 | E | SFF | 10 | 10 | 10 | 10 | 10 | 10 | 10 |
| GYL027 | E | SFF | 5 | 5 | 5 | 5 | 5 | 5 | 5 |
| GYL028 | E | SFF | 8 | 8 | 8 | 8 | 8 | 8 | 8 |
| GYL030 | E | SFF | 3 | 3 | 3 | 3 | 3 | 3 | 3 |
| GYL031 | E | SFF | 4 | 4 | 4 | 4 | 4 | 4 | 4 |
| AT002 | W | NNR | 6 | 6 | 6 | 6 | 6 | 6 | 6 |
| AU002 | W | NNR | 4 | 4 | 4 | 4 | 4 | 4 | 4 |
| AV002 | W | NNR | 8 | 8 | 8 | 8 | 8 | 8 | 8 |
| AW002 | W | NNR | 3 | 3 | 3 | 3 | 3 | 3 | 3 |
| AZ002 | W | NNR | 5 | 5 | 5 | 5 | 5 | 5 | 5 |
| AT003 | W | NNR | 6 | 6 | 6 | 6 | 6 | 6 | 6 |
| AU003 | W | NNR | 6 | 6 | 6 | 6 | 6 | 6 | 6 |
| AV003 | W | NNR | 10 | 10 | 10 | 10 | 10 | 10 | 10 |
| AW003 | W | NNR | 9 | 9 | 9 | 9 | 9 | 9 | 9 |
| AX003 | W | NNR | 6 | 6 | 6 | 6 | 6 | 6 | 6 |
| AY003 | W | NNR | 6 | 6 | 6 | 6 | 6 | 6 | 6 |
| AZ003 | W | NNR | 4 | 4 | 4 | 4 | 4 | 4 | 4 |
| AV004 | W | NNR | 3 | 3 | 3 | 3 | 3 | 3 | 3 |
| AW004 | W | NNR | 9 | 9 | 9 | 9 | 9 | 9 | 9 |
| AX004 | W | NNR | 5 | 5 | 5 | 5 | 5 | 5 | 5 |
| AY004 | W | NNR | 14 | 14 | 14 | 14 | 14 | 14 | 14 |
| AZ004 | W | NNR | 9 | 9 | 9 | 9 | 9 | 9 | 9 |
| AV005 | W | NNR | 7 | 7 | 7 | 7 | 7 | 7 | 7 |
| AW005 | W | NNR | 5 | 5 | 5 | 5 | 5 | 5 | 5 |
| AX005 | W | NNR | 10 | 10 | 10 | 10 | 10 | 10 | 10 |
| BC005 | W | NNR | 5 | 5 | 5 | 5 | 5 | 5 | 5 |
| BB006 | W | NNR | 5 | 5 | 5 | 5 | 5 | 5 | 5 |
| BA007 | W | NNR | 3 | 3 | 3 | 3 | 3 | 3 | 3 |
| BB007 | W | NNR | 7 | 7 | 7 | 7 | 7 | 7 | 7 |
| BC007 | W | NNR | 9 | 9 | 9 | 9 | 9 | 9 | 9 |
| AZ008 | W | NNR | 3 | 3 | 3 | 3 | 3 | 3 | 3 |
| BA008 | W | NNR | 4 | 4 | 4 | 4 | 4 | 4 | 4 |
| BB008 | W | NNR | 4 | 4 | 4 | 4 | 4 | 4 | 4 |
| BC008 | W | NNR | 4 | 4 | 4 | 4 | 4 | 4 | 4 |
| AZ009 | W | NNR | 6 | 6 | 6 | 6 | 6 | 6 | 6 |
| BA009 | W | NNR | 4 | 4 | 4 | 4 | 4 | 4 | 4 |
| BB009 | W | NNR | 5 | 5 | 5 | 5 | 5 | 5 | 5 |
| BC009 | W | NNR | 9 | 9 | 9 | 9 | 9 | 9 | 9 |
| BA010 | W | NNR | 2 | 2 | 2 | 2 | 2 | 2 | 2 |
| BB010 | W | NNR | 5 | 5 | 5 | 5 | 5 | 5 | 5 |
| BC010 | W | NNR | 6 | 6 | 6 | 6 | 6 | 6 | 6 |
| BD010 | W | NNR | 11 | 11 | 11 | 11 | 11 | 11 | 11 |
| BC011 | W | NNR | 4 | 4 | 4 | 4 | 4 | 4 | 4 |
| BD011 | W | NNR | 8 | 8 | 8 | 8 | 8 | 8 | 8 |
| AK014 | W | NNR | 2 | 2 | 2 | 2 | 2 | 2 | 2 |
| AL014 | W | NNR | 1 | 1 | 1 | 1 | 1 | 1 | 1 |
| AI015 | W | NNR | 1 | 1 | 1 | 1 | 1 | 1 | 1 |
| AJ015 | W | NNR | 2 | 2 | 2 | 2 | 2 | 2 | 2 |
| AK015 | W | NNR | 3 | 3 | 3 | 3 | 3 | 3 | 3 |
| AL015 | W | NNR | 1 | 1 | 1 | 1 | 1 | 1 | 1 |
| AI016 | W | NNR | 1 | 1 | 1 | 1 | 1 | 1 | 1 |
| AJ016 | W | NNR | 1 | 1 | 1 | 1 | 1 | 1 | 1 |
| AL016 | W | NNR | 2 | 2 | 2 | 2 | 2 | 2 | 2 |
| AJ017 | W | NNR | 1 | 1 | 1 | 1 | 1 | 1 | 1 |
| AK017 | W | NNR | 2 | 2 | 2 | 2 | 2 | 2 | 2 |
| AL017 | W | NNR | 2 | 2 | 2 | 2 | 2 | 2 | 2 |
| AM017 | W | NNR | 1 | 1 | 1 | 1 | 1 | 1 | 1 |
| G1-1 | W | NNR | 6 | 6 | 6 | 6 | 6 | 6 | 6 |
| G1-1-1 | W | NNR | 6 | 6 | 6 | 6 | 6 | 6 | 6 |
| G1-1-2 | W | NNR | 5 | 5 | 5 | 5 | 5 | 5 | 5 |
| G1-2 | W | NNR | 8 | 8 | 8 | 8 | 8 | 8 | 8 |
| G1-2-1 | W | NNR | 7 | 7 | 7 | 7 | 7 | 7 | 7 |
| G1-2-2 | W | NNR | 10 | 10 | 10 | 10 | 10 | 10 | 10 |
| G1-3 | W | NNR | 12 | 13 | 13.09 | 14 | 14.1 | 12 | 12.08 |
| G1-3-1 | W | NNR | 11 | 11 | 11 | 11 | 11 | 11 | 11 |
| G1-3-2 | W | NNR | 8 | 8 | 8 | 8 | 8 | 8 | 8 |
| G1-4 | W | NNR | 9 | 9 | 9 | 9 | 9 | 9 | 9 |
| G1-4-1 | W | NNR | 2 | 2 | 2 | 2 | 2 | 2 | 2 |
| G1-4-2 | W | NNR | 7 | 7 | 7 | 7 | 7 | 7 | 7 |
| G1-5 | W | NNR | 10 | 10 | 10 | 10 | 10 | 10 | 10 |
| G1-6 | W | NNR | 7 | 7 | 7 | 7 | 7 | 7 | 7 |
| G1-7 | W | NNR | 10 | 10 | 10 | 10 | 10 | 10 | 10 |
| G1-8 | W | NNR | 6 | 6 | 6 | 6 | 6 | 6 | 6 |
| G1-9 | W | NNR | 7 | 7 | 7 | 7 | 7 | 7 | 7 |
| G4-1 | W | NNR | 6 | 6 | 6 | 6 | 6 | 6 | 6 |
| G4-1-1 | W | NNR | 4 | 4 | 4 | 4 | 4 | 4 | 4 |
| G4-1-2 | W | NNR | 5 | 5 | 5 | 5 | 5 | 5 | 5 |
| G4-2 | W | NNR | 5 | 5 | 5 | 5 | 5 | 5 | 5 |
| G4-2-1 | W | NNR | 7 | 7 | 7 | 7 | 7 | 7 | 7 |
| G4-2-2 | W | NNR | 8 | 8 | 8 | 8 | 8 | 8 | 8 |
| G4-3 | W | NNR | 8 | 8 | 8 | 8 | 8 | 8 | 8 |
| G4-3-1 | W | NNR | 8 | 8 | 8 | 8 | 8 | 8 | 8 |
| G4-3-2 | W | NNR | 7 | 7 | 7 | 7 | 7 | 7 | 7 |
| G4-4 | W | NNR | 10 | 10 | 10 | 9 | 9 | 10 | 10 |
| G4-4-1 | W | NNR | 8 | 8 | 8 | 8 | 8 | 8 | 8 |
| G4-4-2 | W | NNR | 8 | 8 | 8 | 8 | 8 | 8 | 8 |
| G4-5 | W | NNR | 9 | 9 | 9 | 9 | 9 | 9 | 9 |
| G4-6 | W | NNR | 3 | 3 | 3 | 3 | 3 | 3 | 3 |
| G4-7 | W | NNR | 9 | 9 | 9 | 9 | 9 | 9 | 9 |
| G5-1 | W | NNR | 6 | 6 | 6 | 6 | 6 | 6 | 6 |
| G5-1-1 | W | NNR | 6 | 6 | 6 | 6 | 6 | 6 | 6 |
| G5-1-2 | W | NNR | 6 | 6 | 6 | 6 | 6 | 6 | 6 |
| G5-2 | W | NNR | 5 | 5 | 5 | 5 | 5 | 5 | 5 |
| G5-2-1 | W | NNR | 3 | 3 | 3 | 3 | 3 | 3 | 3 |
| G5-2-2 | W | NNR | 3 | 3 | 3 | 3 | 3 | 3 | 3 |
| G5-3 | W | NNR | 7 | 7 | 7 | 7 | 7 | 7 | 7 |
| G5-3-1 | W | NNR | 9 | 9 | 9 | 9 | 9 | 9 | 9 |
| G5-3-2 | W | NNR | 7 | 7 | 7 | 7 | 7 | 7 | 7 |
| G5-4 | W | NNR | 7 | 7 | 7 | 7 | 7 | 7 | 7 |
| G5-4-1 | W | NNR | 8 | 8 | 8 | 8 | 8 | 8 | 8 |
| G5-4-2 | W | NNR | 11 | 11 | 11 | 11 | 11 | 11 | 11 |
| G5-5 | W | NNR | 6 | 6 | 6 | 6 | 6 | 6 | 6 |
| G5-6 | W | NNR | 14 | 14 | 14 | 14 | 14 | 14 | 14 |
| G5-7 | W | NNR | 9 | 9 | 9 | 9 | 9 | 9 | 9 |
| G6-1.G6-1-1 | W | NNR | 9 | 9 | 9 | 9 | 9 | 9 | 9 |
| G6-1-2 | W | NNR | 4 | 4 | 4 | 4 | 4 | 4 | 4 |
| G6-2.G6-2-1 | W | NNR | 6 | 6 | 6 | 6 | 6 | 6 | 6 |
| G6-2-2 | W | NNR | 6 | 6 | 6 | 6 | 6 | 6 | 6 |
| G6-3.G6-3-1 | W | NNR | 8 | 8 | 8 | 8 | 8 | 8 | 8 |
| G6-3-2 | W | NNR | 4 | 4 | 4 | 4 | 4 | 4 | 4 |
| G6-4.G6-4-1 | W | NNR | 6 | 6 | 6 | 6 | 6 | 6 | 6 |
| G6-4-2 | W | NNR | 5 | 5 | 5 | 5 | 5 | 5 | 5 |
| G7-1 | W | NNR | 8 | 8 | 8 | 8 | 8 | 8 | 8 |
| G7-1-1 | W | NNR | 7 | 7 | 7 | 7 | 7 | 7 | 7 |
| G7-1-2 | W | NNR | 8 | 8 | 8 | 8 | 8 | 8 | 8 |
| G7-2 | W | NNR | 9 | 9 | 9 | 9 | 9 | 9 | 9 |
| G7-2-1 | W | NNR | 8 | 8 | 8 | 8 | 8 | 8 | 8 |
| G7-2-2 | W | NNR | 5 | 5 | 5 | 5 | 5 | 5 | 5 |
| G7-3 | W | NNR | 8 | 8 | 8 | 8 | 8 | 8 | 8 |
| G7-3-1 | W | NNR | 7 | 7 | 7 | 6 | 6 | 7 | 7 |
| G7-3-2 | W | NNR | 6 | 6 | 6 | 6 | 6 | 6 | 6 |
| G7-4 | W | NNR | 12 | 12 | 12 | 12 | 12 | 12 | 12 |
| G7-5 | W | NNR | 9 | 9 | 9 | 9 | 9 | 9 | 9 |
| Q1-1.2 | W | NNR | 9 | 10 | 10.12 | 11 | 11.14 | 9 | 9.11 |
| Q1-10 | W | NNR | 5 | 5 | 5 | 5 | 5 | 5 | 5 |
| Q1-11 | W | NNR | 6 | 6 | 6 | 6 | 6 | 6 | 6 |
| Q1-12 | W | NNR | 9 | 9 | 9 | 9 | 9 | 9 | 9 |
| Q1-13 | W | NNR | 3 | 3 | 3 | 3 | 3 | 3 | 3 |
| Q1-14 | W | NNR | 6 | 6 | 6 | 6 | 6 | 6 | 6 |
| Q1-3 | W | NNR | 8 | 8 | 8 | 8 | 8 | 8 | 8 |
| Q1-4 | W | NNR | 9 | 9 | 9 | 9 | 9 | 9 | 9 |
| Q1-5 | W | NNR | 6 | 6 | 6 | 6 | 6 | 6 | 6 |
| Q1-6 | W | NNR | 6 | 6 | 6 | 6 | 6 | 6 | 6 |
| Q1-7 | W | NNR | 5 | 5 | 5 | 5 | 5 | 5 | 5 |
| Q1-8 | W | NNR | 4 | 4 | 4 | 4 | 4 | 4 | 4 |
| Q1-9 | W | NNR | 8 | 8 | 8 | 8 | 8 | 8 | 8 |
| Q2-1.2 | W | NNR | 10 | 10 | 10 | 10 | 10 | 10 | 10 |
| Q2-10 | W | NNR | 5 | 5 | 5 | 5 | 5 | 5 | 5 |
| Q2-3 | W | NNR | 7 | 7 | 7 | 7 | 7 | 7 | 7 |
| Q2-4.5 | W | NNR | 6 | 6 | 6 | 6 | 6 | 6 | 6 |
| Q2-6 | W | NNR | 10 | 10 | 10 | 10 | 10 | 10 | 10 |
| Q2-7 | W | NNR | 7 | 7 | 7 | 7 | 7 | 7 | 7 |
| Q2-8.9 | W | NNR | 10 | 10 | 10 | 10 | 10 | 10 | 10 |
| Q3-1 | W | NNR | 8 | 8 | 8 | 8 | 8 | 8 | 8 |
| Q3-11 | W | NNR | 5 | 5 | 5 | 5 | 5 | 5 | 5 |
| Q3-2 | W | NNR | 9 | 9 | 9 | 9 | 9 | 9 | 9 |
| Q3-3.4.5.6 | W | NNR | 10 | 10 | 10 | 10 | 10 | 10 | 10 |
| Q3-7 | W | NNR | 1 | 1 | 1 | 1 | 1 | 1 | 1 |
| Q3-8 | W | NNR | 7 | 7 | 7 | 7 | 7 | 7 | 7 |
| Q3-9.10 | W | NNR | 6 | 6 | 6 | 6 | 6 | 6 | 6 |
| Q4-1 | W | NNR | 4 | 4 | 4 | 4 | 4 | 4 | 4 |
| Q4-10 | W | NNR | 8 | 8 | 8 | 8 | 8 | 8 | 8 |
| Q4-11 | W | NNR | 6 | 6 | 6 | 6 | 6 | 6 | 6 |
| Q4-12 | W | NNR | 2 | 2 | 2 | 2 | 2 | 2 | 2 |
| Q4-2 | W | NNR | 4 | 4 | 4 | 4 | 4 | 4 | 4 |
| Q4-3.4 | W | NNR | 9 | 9 | 9 | 9 | 9 | 9 | 9 |
| Q4-6 | W | NNR | 8 | 8 | 8 | 8 | 8 | 8 | 8 |
| Q4-8 | W | NNR | 4 | 4 | 4 | 4 | 4 | 4 | 4 |
| Q4-9 | W | NNR | 2 | 2 | 2 | 2 | 2 | 2 | 2 |
| Q5-1 | W | NNR | 7 | 7 | 7 | 7 | 7 | 7 | 7 |
| Q5-2 | W | NNR | 11 | 11 | 11 | 11 | 11 | 11 | 11 |
| Q5-3 | W | NNR | 6 | 6 | 6 | 6 | 6 | 6 | 6 |
| Q5-4 | W | NNR | 6 | 6 | 6 | 6 | 6 | 6 | 6 |
| Q5-5 | W | NNR | 7 | 7 | 7 | 7 | 7 | 7 | 7 |
| G5-X | W | NNR | 6 | 6 | 6 | 6 | 6 | 6 | 6 |
| MLa001 | E | MLa | 12 | 12 | 12 | 12 | 12 | 12 | 12 |
| MLa002 | E | MLa | 8 | 8 | 8 | 8 | 8 | 8 | 8 |
| MLa003 | E | MLa | 13 | 13 | 13 | 13 | 13 | 13 | 13 |
| MLa004 | E | MLa | 13 | 13 | 13 | 13 | 13 | 13 | 13 |
| MLa005 | E | MLa | 12 | 12 | 12 | 12 | 12 | 12 | 12 |
| MLa006 | E | MLa | 8 | 8 | 8 | 8 | 8 | 8 | 8 |
| MLa007 | E | MLa | 8 | 8 | 8 | 8 | 8 | 8 | 8 |
| MLa008 | E | MLa | 11 | 11 | 11 | 11 | 11 | 11 | 11 |
| MLa009 | E | MLa | 7 | 7 | 7 | 7 | 7 | 7 | 7 |
| MLa010 | E | MLa | 12 | 12 | 12 | 12 | 12 | 12 | 12 |
| MLa012 | E | MLa | 11 | 11 | 11 | 11 | 11 | 11 | 11 |
| MLa013 | E | MLa | 7 | 7 | 7 | 7 | 7 | 7 | 7 |
| MLa014 | E | MLa | 4 | 4 | 4 | 4 | 4 | 4 | 4 |
| MLa015 | E | MLa | 8 | 8 | 8 | 8 | 8 | 8 | 8 |
| MLa016 | E | MLa | 8 | 8 | 8 | 8 | 8 | 8 | 8 |
| MLa017 | E | MLa | 3 | 3 | 3 | 3 | 3 | 3 | 3 |
| MLa018 | E | MLa | 9 | 9 | 9 | 9 | 9 | 9 | 9 |
| MLa019 | E | MLa | 9 | 9 | 9 | 9 | 9 | 9 | 9 |
| MLa020 | E | MLa | 8 | 8 | 8 | 8 | 8 | 8 | 8 |
| MLa021 | E | MLa | 5 | 5 | 5 | 5 | 5 | 5 | 5 |
| MLa022 | E | MLa | 5 | 5 | 5 | 5 | 5 | 5 | 5 |
| MLa023 | E | MLa | 8 | 8 | 8 | 8 | 8 | 8 | 8 |
| MLa024 | E | MLa | 9 | 9 | 9 | 9 | 9 | 9 | 9 |
| MLa025 | E | MLa | 11 | 11 | 11 | 11 | 11 | 11 | 11 |
| MLa026 | E | MLa | 13 | 13 | 13 | 13 | 13 | 13 | 13 |
| MLa037 | E | MLa | 5 | 5 | 5 | 5 | 5 | 5 | 5 |
| MLa038 | E | MLa | 4 | 4 | 4 | 4 | 4 | 4 | 4 |
| MLa039 | E | MLa | 5 | 5 | 5 | 5 | 5 | 5 | 5 |
| MLa041 | E | MLa | 6 | 6 | 6 | 6 | 6 | 6 | 6 |
| MLa042 | E | MLa | 7 | 7 | 7 | 7 | 7 | 7 | 7 |
| MLa043 | E | MLa | 6 | 6 | 6 | 6 | 6 | 6 | 6 |
| MLa045 | E | MLa | 8 | 8 | 8 | 8 | 8 | 8 | 8 |
| MLa046 | E | MLa | 13 | 13 | 13 | 13 | 13 | 13 | 13 |
| MLa047 | E | MLa | 14 | 14 | 14 | 14 | 14 | 14 | 14 |
| MLa048 | E | MLa | 11 | 11 | 11 | 11 | 11 | 11 | 11 |
| MLa049 | E | MLa | 13 | 13 | 13 | 13 | 13 | 13 | 13 |
| MLa050 | E | MLa | 11 | 11 | 11 | 11 | 11 | 11 | 11 |
| MLa051 | E | MLa | 14 | 14 | 14 | 14 | 14 | 14 | 14 |
| MLa052 | E | MLa | 11 | 11 | 11 | 11 | 11 | 11 | 11 |
| MLa053 | E | MLa | 4 | 4 | 4 | 4 | 4 | 4 | 4 |
| MLa054 | E | MLa | 4 | 4 | 4 | 4 | 4 | 4 | 4 |
| MLa055 | E | MLa | 4 | 4 | 4 | 4 | 4 | 4 | 4 |
| MLa056 | E | MLa | 6 | 6 | 6 | 6 | 6 | 6 | 6 |
| MLa057 | E | MLa | 11 | 11 | 11 | 11 | 11 | 11 | 11 |
| MLa058 | E | MLa | 6 | 6 | 6 | 6 | 6 | 6 | 6 |
| MLa059 | E | MLa | 5 | 5 | 5 | 5 | 5 | 5 | 5 |
| MLa061 | E | MLa | 5 | 5 | 5 | 5 | 5 | 5 | 5 |
| MLa062 | E | MLa | 6 | 6 | 6 | 6 | 6 | 6 | 6 |
| MLa063 | E | MLa | 2 | 2 | 2 | 2 | 2 | 2 | 2 |
| MLa064 | E | MLa | 3 | 3 | 3 | 3 | 3 | 3 | 3 |
| MLa066 | E | MLa | 3 | 3 | 3 | 3 | 3 | 3 | 3 |
| MLa067 | E | MLa | 3 | 3 | 3 | 3 | 3 | 3 | 3 |
| MLa068 | E | MLa | 3 | 3 | 3 | 3 | 3 | 3 | 3 |
| MLa069 | E | MLa | 5 | 5 | 5 | 5 | 5 | 5 | 5 |
| MLa070 | E | MLa | 3 | 3 | 3 | 3 | 3 | 3 | 3 |
| MLa071 | E | MLa | 6 | 6 | 6 | 6 | 6 | 6 | 6 |
| MLa072 | E | MLa | 4 | 4 | 4 | 4 | 4 | 4 | 4 |
| MLa073 | E | MLa | 9 | 9 | 9 | 9 | 9 | 9 | 9 |
| MLa074 | E | MLa | 4 | 4 | 4 | 4 | 4 | 4 | 4 |
| MLa075 | E | MLa | 7 | 7 | 7 | 7 | 7 | 7 | 7 |
| MLa076 | E | MLa | 5 | 5 | 5 | 5 | 5 | 5 | 5 |
| MLa077 | E | MLa | 11 | 11 | 11 | 11 | 11 | 11 | 11 |
| MLa078 | E | MLa | 4 | 4 | 4 | 4 | 4 | 4 | 4 |
| MLa079 | E | MLa | 7 | 7 | 7 | 7 | 7 | 7 | 7 |
| MLa080 | E | MLa | 4 | 4 | 4 | 4 | 4 | 4 | 4 |
| MLa081 | E | MLa | 5 | 5 | 5 | 5 | 5 | 5 | 5 |
| MLa082 | E | MLa | 5 | 5 | 5 | 5 | 5 | 5 | 5 |
| MLa085 | E | MLa | 3 | 3 | 3 | 3 | 3 | 3 | 3 |
| MLa086 | E | MLa | 5 | 5 | 5 | 5 | 5 | 5 | 5 |
| MLa087 | E | MLa | 9 | 9 | 9 | 9 | 9 | 9 | 9 |
| MLa089 | E | MLa | 5 | 5 | 5 | 5 | 5 | 5 | 5 |
| MLa090 | E | MLa | 2 | 2 | 2 | 2 | 2 | 2 | 2 |
| MLa091 | E | MLa | 7 | 7 | 7 | 7 | 7 | 7 | 7 |
| MLa092 | E | MLa | 5 | 5 | 5 | 5 | 5 | 5 | 5 |
| MLa093 | E | MLa | 6 | 6 | 6 | 6 | 6 | 6 | 6 |
| MLa094 | E | MLa | 11 | 11 | 11 | 11 | 11 | 11 | 11 |
| MLa095 | E | MLa | 6 | 6 | 6 | 6 | 6 | 6 | 6 |
| MLa096 | E | MLa | 8 | 8 | 8 | 8 | 8 | 8 | 8 |
| MLa097 | E | MLa | 8 | 8 | 8 | 8 | 8 | 8 | 8 |
| MLa098 | E | MLa | 10 | 10 | 10 | 10 | 10 | 10 | 10 |
| MLa101 | E | MLa | 7 | 7 | 7 | 7 | 7 | 7 | 7 |
| MLa102 | E | MLa | 3 | 3 | 3 | 3 | 3 | 3 | 3 |
| MLa103 | E | MLa | 10 | 10 | 10 | 10 | 10 | 10 | 10 |
| MLa104 | E | MLa | 6 | 6 | 6 | 6 | 6 | 6 | 6 |
| MLa105 | E | MLa | 8 | 8 | 8 | 8 | 8 | 8 | 8 |
| MLa107 | E | MLa | 8 | 8 | 8 | 8 | 8 | 8 | 8 |
| MLa108 | E | MLa | 7 | 7 | 7 | 7 | 7 | 7 | 7 |
| MLa109 | E | MLa | 12 | 12 | 12 | 12 | 12 | 12 | 12 |
| MLa110 | E | MLa | 6 | 6 | 6 | 6 | 6 | 6 | 6 |
| MLa111 | E | MLa | 7 | 7 | 7 | 7 | 7 | 7 | 7 |
| MLa113 | E | MLa | 8 | 8 | 8 | 8 | 8 | 8 | 8 |
| MLa114 | E | MLa | 10 | 10 | 10 | 10 | 10 | 10 | 10 |
| MLa115 | E | MLa | 7 | 7 | 7 | 7 | 7 | 7 | 7 |
| MLa117 | E | MLa | 10 | 10 | 10 | 10 | 10 | 10 | 10 |
| MLa118 | E | MLa | 12 | 12 | 12 | 12 | 12 | 12 | 12 |
| MLa009_171819 | E | MLa | 9 | 9 | 9 | 9 | 9 | 9 | 9 |
| MLa010_171819 | E | MLa | 8 | 8 | 8 | 8 | 8 | 8 | 8 |
| MLa011_171819 | E | MLa | 10 | 10 | 10 | 10 | 10 | 10 | 10 |
| MLa012_171819 | E | MLa | 11 | 11 | 11 | 11 | 11 | 11 | 11 |
| MLa013_171819 | E | MLa | 13 | 13 | 13 | 13 | 13 | 13 | 13 |
| MLa014_171819 | E | MLa | 12 | 12 | 12 | 12 | 12 | 12 | 12 |
| MLa015_171819 | E | MLa | 11 | 11 | 11 | 11 | 11 | 11 | 11 |
| MLa016_171819 | E | MLa | 7 | 7 | 7 | 7 | 7 | 7 | 7 |
| MLa017_171819 | E | MLa | 9 | 9 | 9 | 9 | 9 | 9 | 9 |
| MLa018_171819 | E | MLa | 9 | 9 | 9 | 9 | 9 | 9 | 9 |
| MLa019_171819 | E | MLa | 4 | 4 | 4 | 4 | 4 | 4 | 4 |
| MLa020_171819 | E | MLa | 5 | 5 | 5 | 5 | 5 | 5 | 5 |
| Mengyuan001_171819 | E | MLa | 5 | 5 | 5 | 5 | 5 | 5 | 5 |
| Mengyuan015_171819 | E | MLa | 10 | 10 | 10 | 10 | 10 | 10 | 10 |
| Mengyuan016_171819 | E | MLa | 10 | 10 | 10 | 10 | 10 | 10 | 10 |
| Mengyuan017_171819 | E | MLa | 7 | 7 | 7 | 7 | 7 | 7 | 7 |
| Mengyuan018_171819 | E | MLa | 5 | 5 | 5 | 5 | 5 | 5 | 5 |
| Mengyuan019_171819 | E | MLa | 8 | 8 | 8 | 8 | 8 | 8 | 8 |
| Mengyuan020_171819 | E | MLa | 5 | 5 | 5 | 5 | 5 | 5 | 5 |
| Mengyuan011_17 | E | MLa | 2 | 2 | 2 | 2 | 2 | 2 | 2 |
| NGS14-1-5 | E | MLa | 2 | 2 | 2 | 2 | 2 | 2 | 2 |
| NGS14-1-6 | E | MLa | 1 | 1 | 1 | 1 | 1 | 1 | 1 |
| NGS14-1-10 | E | MLa | 1 | 1 | 1 | 1 | 1 | 1 | 1 |
| NGS14-2-1 | E | MLa | 1 | 1 | 1 | 1 | 1 | 1 | 1 |
| NGS14-2-3 | E | MLa | 1 | 1 | 1 | 1 | 1 | 1 | 1 |
| NGS14-2-7 | E | MLa | 1 | 1 | 1 | 1 | 1 | 1 | 1 |
| NGS14-2-8 | E | MLa | 1 | 1 | 1 | 1 | 1 | 1 | 1 |
| NGS14-2-9 | E | MLa | 1 | 1 | 1 | 0 | 0 | 1 | 1 |
| NGS14-3-5 | E | MLa | 1 | 1 | 1 | 1 | 1 | 1 | 1 |
| NGS14-3-7 | E | MLa | 2 | 2 | 2 | 2 | 2 | 2 | 2 |
| NGS14-4-1 | E | MLa | 1 | 1 | 1 | 1 | 1 | 1 | 1 |
| NGS14-4-2 | E | MLa | 2 | 2 | 2 | 2 | 2 | 2 | 2 |
| NGS15-1-1 | E | MLa | 4 | 5 | 5.31 | 6 | 6.38 | 4 | 4.25 |
| NGS15-1-3 | E | MLa | 4 | 4 | 4 | 4 | 4 | 4 | 4 |
| NGS15-1-4 | E | MLa | 2 | 2 | 2 | 2 | 2 | 2 | 2 |
| NGS15-1-5 | E | MLa | 3 | 3 | 3 | 3 | 3 | 3 | 3 |
| NGS15-1-6 | E | MLa | 2 | 2 | 2 | 2 | 2 | 2 | 2 |
| NGS15-2-1 | E | MLa | 1 | 1 | 1 | 1 | 1 | 1 | 1 |
| NGS15-2-2 | E | MLa | 4 | 4 | 4 | 4 | 4 | 4 | 4 |
| NGS15-2-3 | E | MLa | 2 | 2 | 2 | 2 | 2 | 2 | 2 |
| NGS15-2-4 | E | MLa | 3 | 3 | 3 | 3 | 3 | 3 | 3 |
| NGS15-2-5 | E | MLa | 1 | 1 | 1 | 1 | 1 | 1 | 1 |
| NGS15-2-6 | E | MLa | 6 | 6 | 6 | 6 | 6 | 6 | 6 |
| NGS15-2-7 | E | MLa | 3 | 3 | 3 | 3 | 3 | 3 | 3 |
| NGS15-001 | E | MLa | 3 | 3 | 3 | 3 | 3 | 3 | 3 |
| NGS15-003 | E | MLa | 2 | 2 | 2 | 2 | 2 | 2 | 2 |
| NGS15-005 | E | MLa | 2 | 2 | 2 | 2 | 2 | 2 | 2 |
| NGS15-006 | E | MLa | 5 | 5 | 5 | 5 | 5 | 5 | 5 |
| NGS15-007 | E | MLa | 3 | 3 | 3 | 3 | 3 | 3 | 3 |
| NGS15-008 | E | MLa | 2 | 2 | 2 | 2 | 2 | 2 | 2 |
| NGS15-009 | E | MLa | 5 | 5 | 5 | 5 | 5 | 5 | 5 |
| NGS15-010 | E | MLa | 4 | 4 | 4 | 4 | 4 | 4 | 4 |
| NGS15-011 | E | MLa | 5 | 5 | 5 | 5 | 5 | 5 | 5 |
| NGS15-012 | E | MLa | 2 | 2 | 2 | 2 | 2 | 2 | 2 |
| NGS15-014 | E | MLa | 2 | 2 | 2 | 2 | 2 | 2 | 2 |
| NGS15-015 | E | MLa | 3 | 3 | 3 | 3 | 3 | 3 | 3 |
| NGS15-016 | E | MLa | 2 | 2 | 2 | 2 | 2 | 2 | 2 |
| NGS15-017 | E | MLa | 3 | 3 | 3 | 3 | 3 | 3 | 3 |
| NGS15-018 | E | MLa | 3 | 3 | 3 | 3 | 3 | 3 | 3 |
| NGS15-019 | E | MLa | 7 | 7 | 7 | 7 | 7 | 7 | 7 |
| NGS15-020 | E | MLa | 4 | 4 | 4 | 4 | 4 | 4 | 4 |
| NGS15-021 | E | MLa | 1 | 1 | 1 | 1 | 1 | 1 | 1 |
| NGS15-022 | E | MLa | 1 | 1 | 1 | 1 | 1 | 1 | 1 |
| NGS15-023 | E | MLa | 4 | 4 | 4 | 4 | 4 | 4 | 4 |
| NGS15-024 | E | MLa | 3 | 3 | 3 | 3 | 3 | 3 | 3 |
| NGS15-025 | E | MLa | 2 | 2 | 2 | 2 | 2 | 2 | 2 |
| NGS15-027 | E | MLa | 2 | 2 | 2 | 2 | 2 | 2 | 2 |
| NGS15-028 | E | MLa | 1 | 1 | 1 | 1 | 1 | 1 | 1 |
| mengsong4-1 | W | BNR | 2 | 2 | 2 | 2 | 2 | 2 | 2 |
| mengsong4-2 | W | BNR | 2 | 2 | 2 | 2 | 2 | 2 | 2 |
| mengsong4-3 | W | BNR | 1 | 1 | 1 | 1 | 1 | 1 | 1 |
| mengsong4-4 | W | BNR | 1 | 1 | 1 | 1 | 1 | 1 | 1 |
| mengsong4-5 | W | BNR | 6 | 6 | 6 | 6 | 6 | 6 | 6 |
| mengsong5-1 | W | BNR | 1 | 1 | 1 | 1 | 1 | 1 | 1 |
| mengsong5-2 | W | BNR | 1 | 1 | 1 | 1 | 1 | 1 | 1 |
| BL2-7 | W | BNR | 2 | 2 | 2 | 2 | 2 | 2 | 2 |
| BL3-1 | W | BNR | 5 | 5 | 5 | 5 | 5 | 5 | 5 |
| BL4-3 | W | BNR | 1 | 1 | 1 | 1 | 1 | 1 | 1 |
| BL5-4 | W | BNR | 3 | 3 | 3 | 2 | 2 | 3 | 3 |
| BL6-3 | W | BNR | 2 | 2 | 2 | 2 | 2 | 2 | 2 |
| BL8-3 | W | BNR | 2 | 2 | 2 | 2 | 2 | 2 | 2 |
| BL8-5 | W | BNR | 1 | 1 | 1 | 1 | 1 | 1 | 1 |
| MLa083 | E | MLa | 7 | 7 | 7 | 7 | 7 | 7 | 7 |
| G4-X | W | NNR | 1 | 1 | 1 | 1 | 1 | 1 | 1 |
| AK16 | W | NNR | 2 | 2 | 2 | 2 | 2 | 2 | 2 |
| AM15 | W | NNR | 1 | 1 | 1 | 1 | 1 | 1 | 1 |
| AJ018 | W | NNR | 1 | 1 | 1 | 1 | 1 | 1 | 1 |
| AJ019 | W | NNR | 1 | 1 | 1 | 1 | 1 | 1 | 1 |
| AJ020 | W | NNR | 1 | 1 | 1 | 1 | 1 | 1 | 1 |
| MY001 | E | MY | 5 | 5 | 5 | 5 | 5 | 5 | 5 |
| MY002 | E | MY | 4 | 4 | 4 | 4 | 4 | 4 | 4 |
| MY003 | E | MY | 5 | 5 | 5 | 5 | 5 | 5 | 5 |
| MY004 | E | MY | 5 | 5 | 5 | 5 | 5 | 5 | 5 |
| MY005 | E | MY | 7 | 7 | 7 | 7 | 7 | 7 | 7 |
| MY006 | E | MY | 8 | 8 | 8 | 8 | 8 | 8 | 8 |
| MY007 | E | MY | 6 | 6 | 6 | 6 | 6 | 6 | 6 |
| MY008 | E | MY | 8 | 8 | 8 | 8 | 8 | 8 | 8 |
| MY009 | E | MY | 8 | 8 | 8 | 8 | 8 | 8 | 8 |
| MY010 | E | MY | 12 | 12 | 12 | 12 | 12 | 12 | 12 |
| MY011 | E | MY | 10 | 10 | 10 | 10 | 10 | 10 | 10 |
| MY012 | E | MY | 6 | 6 | 6 | 6 | 6 | 6 | 6 |
| MY013 | E | MY | 11 | 11 | 11 | 11 | 11 | 11 | 11 |
| MY014 | E | MY | 14 | 14 | 14 | 14 | 14 | 14 | 14 |
| MY015 | E | MY | 12 | 12 | 12 | 12 | 12 | 12 | 12 |
| MY016 | E | MY | 5 | 5 | 5 | 5 | 5 | 5 | 5 |
| MY017 | E | MY | 14 | 14 | 14 | 14 | 14 | 14 | 14 |
| MY018 | E | MY | 15 | 15 | 15 | 15 | 15 | 15 | 15 |
| MY019 | E | MY | 7 | 7 | 7 | 7 | 7 | 7 | 7 |
| MY020 | E | MY | 16 | 16 | 16 | 16 | 16 | 16 | 16 |
| MY021 | E | MY | 11 | 11 | 11 | 11 | 11 | 11 | 11 |
| MY023 | E | MY | 12 | 12 | 12 | 12 | 12 | 12 | 12 |
| MY024 | E | MY | 8 | 8 | 8 | 8 | 8 | 8 | 8 |
| MY025 | E | MY | 11 | 11 | 11 | 11 | 11 | 11 | 11 |
| MY026 | E | MY | 10 | 10 | 10 | 10 | 10 | 10 | 10 |
| MY027 | E | MY | 10 | 10 | 10 | 10 | 10 | 10 | 10 |
| MY028 | E | MY | 7 | 7 | 7 | 7 | 7 | 7 | 7 |
| MY029 | E | MY | 9 | 9 | 9 | 9 | 9 | 9 | 9 |
| MY030 | E | MY | 9 | 9 | 9 | 9 | 9 | 9 | 9 |
| MY031 | E | MY | 5 | 5 | 5 | 5 | 5 | 5 | 5 |
| MY032 | E | MY | 10 | 10 | 10 | 10 | 10 | 10 | 10 |
| MY033 | E | MY | 14 | 14 | 14 | 14 | 14 | 14 | 14 |
| MY034 | E | MY | 13 | 13 | 13 | 13 | 13 | 13 | 13 |
| MY035 | E | MY | 4 | 4 | 4 | 4 | 4 | 4 | 4 |
| MY036 | E | MY | 8 | 8 | 8 | 8 | 8 | 8 | 8 |
| MY037 | E | MY | 17 | 17 | 17 | 17 | 17 | 17 | 17 |
| MY038 | E | MY | 12 | 12 | 12 | 12 | 12 | 12 | 12 |
| MY039 | E | MY | 10 | 10 | 10 | 10 | 10 | 10 | 10 |
| MY040 | E | MY | 13 | 13 | 13 | 13 | 13 | 13 | 13 |
| MY041 | E | MY | 8 | 8 | 8 | 8 | 8 | 8 | 8 |
| MY042 | E | MY | 12 | 12 | 12 | 12 | 12 | 12 | 12 |
| MY043 | E | MY | 8 | 8 | 8 | 8 | 8 | 8 | 8 |
| MY044 | E | MY | 7 | 7 | 7 | 7 | 7 | 7 | 7 |
| MY045 | E | MY | 6 | 6 | 6 | 6 | 6 | 6 | 6 |
| MY046 | E | MY | 12 | 12 | 12 | 12 | 12 | 12 | 12 |
| MY047 | E | MY | 5 | 5 | 5 | 5 | 5 | 5 | 5 |
| MY048 | E | MY | 8 | 8 | 8 | 8 | 8 | 8 | 8 |
| MY049 | E | MY | 10 | 10 | 10 | 10 | 10 | 10 | 10 |
| MY050 | E | MY | 10 | 10 | 10 | 10 | 10 | 10 | 10 |
| MY051 | E | MY | 8 | 8 | 8 | 8 | 8 | 8 | 8 |
| MY052 | E | MY | 7 | 7 | 7 | 7 | 7 | 7 | 7 |
| MY053 | E | MY | 4 | 4 | 4 | 4 | 4 | 4 | 4 |
| MY054 | E | MY | 6 | 6 | 6 | 6 | 6 | 6 | 6 |
| MY055 | E | MY | 9 | 9 | 9 | 9 | 9 | 9 | 9 |
| MY057 | E | MY | 11 | 11 | 11 | 11 | 11 | 11 | 11 |
| MY058 | E | MY | 10 | 10 | 10 | 10 | 10 | 10 | 10 |
| MY059 | E | MY | 8 | 8 | 8 | 8 | 8 | 8 | 8 |
| MY060 | E | MY | 11 | 11 | 11 | 11 | 11 | 11 | 11 |
| MY061 | E | MY | 13 | 13 | 13 | 13 | 13 | 13 | 13 |
| MY062 | E | MY | 4 | 4 | 4 | 4 | 4 | 4 | 4 |
| MY063 | E | MY | 4 | 4 | 4 | 4 | 4 | 4 | 4 |
| MY064 | E | MY | 11 | 11 | 11 | 11 | 11 | 11 | 11 |
| MY065 | E | MY | 9 | 9 | 9 | 9 | 9 | 9 | 9 |
| MY066 | E | MY | 8 | 8 | 8 | 8 | 8 | 8 | 8 |
| MY067 | E | MY | 11 | 11 | 11 | 11 | 11 | 11 | 11 |
| MY068 | E | MY | 8 | 8 | 8 | 8 | 8 | 8 | 8 |
| MY069 | E | MY | 8 | 8 | 8 | 8 | 8 | 8 | 8 |
| MY070 | E | MY | 6 | 6 | 6 | 6 | 6 | 6 | 6 |
| MY071 | E | MY | 5 | 5 | 5 | 5 | 5 | 5 | 5 |
| MY072 | E | MY | 9 | 9 | 9 | 9 | 9 | 9 | 9 |
| MY073 | E | MY | 8 | 8 | 8 | 8 | 8 | 8 | 8 |
| MY074 | E | MY | 9 | 9 | 9 | 9 | 9 | 9 | 9 |
| MY075 | E | MY | 7 | 7 | 7 | 7 | 7 | 7 | 7 |
| MY076 | E | MY | 11 | 11 | 11 | 11 | 11 | 11 | 11 |
| MY077 | E | MY | 7 | 7 | 7 | 7 | 7 | 7 | 7 |
| MY078 | E | MY | 4 | 4 | 4 | 4 | 4 | 4 | 4 |
| MY079 | E | MY | 8 | 8 | 8 | 8 | 8 | 8 | 8 |
| MY080 | E | MY | 7 | 7 | 7 | 7 | 7 | 7 | 7 |
| MY081 | E | MY | 5 | 5 | 5 | 5 | 5 | 5 | 5 |
| MY082 | E | MY | 9 | 9 | 9 | 9 | 9 | 9 | 9 |
| MY083 | E | MY | 6 | 6 | 6 | 6 | 6 | 6 | 6 |
| MY084 | E | MY | 6 | 6 | 6 | 6 | 6 | 6 | 6 |
| MY085 | E | MY | 6 | 6 | 6 | 6 | 6 | 6 | 6 |
| MY086 | E | MY | 5 | 5 | 5 | 5 | 5 | 5 | 5 |
| MY087 | E | MY | 9 | 9 | 9 | 9 | 9 | 9 | 9 |
| MY088 | E | MY | 11 | 11 | 11 | 11 | 11 | 11 | 11 |
| MY089 | E | MY | 8 | 8 | 8 | 8 | 8 | 8 | 8 |
| MY090 | E | MY | 9 | 9 | 9 | 9 | 9 | 9 | 9 |
| MY091 | E | MY | 15 | 15 | 15 | 15 | 15 | 15 | 15 |
| MY092 | E | MY | 15 | 15 | 15 | 15 | 15 | 15 | 15 |
| MY093 | E | MY | 11 | 11 | 11 | 11 | 11 | 11 | 11 |
| MY094 | E | MY | 3 | 3 | 3 | 3 | 3 | 3 | 3 |
| MY095 | E | MY | 8 | 8 | 8 | 8 | 8 | 8 | 8 |
| MY096 | E | MY | 6 | 6 | 6 | 6 | 6 | 6 | 6 |
| MY097 | E | MY | 12 | 12 | 12 | 12 | 12 | 12 | 12 |
| MY098 | E | MY | 7 | 7 | 7 | 7 | 7 | 7 | 7 |
| MY099 | E | MY | 10 | 10 | 10 | 10 | 10 | 10 | 10 |
| MY100 | E | MY | 9 | 9 | 9 | 9 | 9 | 9 | 9 |
| MY101 | E | MY | 10 | 10 | 10 | 10 | 10 | 10 | 10 |
| MY103 | E | MY | 7 | 7 | 7 | 7 | 7 | 7 | 7 |
| MY104 | E | MY | 10 | 11 | 11.11 | 12 | 12.12 | 10 | 10.1 |
| MY105 | E | MY | 10 | 10 | 10 | 10 | 10 | 10 | 10 |
| MY106 | E | MY | 11 | 11 | 11 | 11 | 11 | 11 | 11 |
| MY107 | E | MY | 13 | 13 | 13 | 13 | 13 | 13 | 13 |
| MY108 | E | MY | 8 | 8 | 8 | 8 | 8 | 8 | 8 |
| MY109 | E | MY | 11 | 11 | 11 | 11 | 11 | 11 | 11 |
| MY110 | E | MY | 10 | 10 | 10 | 10 | 10 | 10 | 10 |
| MY111 | E | MY | 5 | 5 | 5 | 5 | 5 | 5 | 5 |
| MY112 | E | MY | 12 | 12 | 12 | 12 | 12 | 12 | 12 |
| MY113 | E | MY | 5 | 5 | 5 | 5 | 5 | 5 | 5 |
| MY114 | E | MY | 6 | 6 | 6 | 6 | 6 | 6 | 6 |
| MY115 | E | MY | 6 | 6 | 6 | 6 | 6 | 6 | 6 |
| MY116 | E | MY | 8 | 8 | 8 | 8 | 8 | 8 | 8 |
| MY117 | E | MY | 7 | 8 | 8.16 | 9 | 9.18 | 7 | 7.14 |
| MY118 | E | MY | 6 | 6 | 6 | 6 | 6 | 6 | 6 |
| MY119 | E | MY | 5 | 5 | 5 | 5 | 5 | 5 | 5 |
| MY120 | E | MY | 10 | 10 | 10 | 10 | 10 | 10 | 10 |
| manla001 | E | YNR | 8 | 8 | 8 | 8 | 8 | 8 | 8 |
| manla002 | E | YNR | 2 | 2 | 2 | 2 | 2 | 2 | 2 |
| manla003 | E | YNR | 9 | 9 | 9 | 9 | 9 | 9 | 9 |
| manla004 | E | YNR | 7 | 7 | 7 | 7 | 7 | 7 | 7 |
| manla005 | E | YNR | 4 | 4 | 4 | 4 | 4 | 4 | 4 |
| manla006 | E | YNR | 8 | 8 | 8 | 8 | 8 | 8 | 8 |
| manla007 | E | YNR | 9 | 9 | 9 | 9 | 9 | 9 | 9 |
| manla008 | E | YNR | 6 | 6 | 6 | 6 | 6 | 6 | 6 |
| manla009 | E | YNR | 1 | 1 | 1 | 1 | 1 | 1 | 1 |
| manla010 | E | YNR | 16 | 16 | 16 | 16 | 16 | 16 | 16 |
| manla011 | E | YNR | 12 | 12 | 12 | 12 | 12 | 12 | 12 |
| manla012 | E | YNR | 14 | 14 | 14 | 14 | 14 | 14 | 14 |
| manla013 | E | YNR | 11 | 11 | 11 | 11 | 11 | 11 | 11 |
| manla014 | E | YNR | 9 | 9 | 9 | 9 | 9 | 9 | 9 |
| fanghuotongdao1 | E | YNR | 10 | 10 | 10 | 10 | 10 | 10 | 10 |
| fanghuotongdao2 | E | YNR | 10 | 10 | 10 | 10 | 10 | 10 | 10 |
| fanghuotongdao3 | E | YNR | 12 | 12 | 12 | 12 | 12 | 12 | 12 |
| fanghuotongdao4 | E | YNR | 7 | 7 | 7 | 7 | 7 | 7 | 7 |
| fanghuotongdao5 | E | YNR | 4 | 4 | 4 | 4 | 4 | 4 | 4 |
| fanghuotongdao6 | E | YNR | 10 | 10 | 10 | 10 | 10 | 10 | 10 |
| fanghuotongdao7 | E | YNR | 5 | 5 | 5 | 5 | 5 | 5 | 5 |
| fanghuotongdao8 | E | YNR | 4 | 4 | 4 | 4 | 4 | 4 | 4 |
| xiaohebian1 | E | YNR | 8 | 8 | 8 | 8 | 8 | 8 | 8 |
| xiaohebian2 | E | YNR | 7 | 7 | 7 | 7 | 7 | 7 | 7 |
| xiaohebian3 | E | YNR | 15 | 15 | 15 | 15 | 15 | 15 | 15 |
| xiaohebian4 | E | YNR | 12 | 12 | 12 | 12 | 12 | 12 | 12 |
| xiaohebian5 | E | YNR | 8 | 8 | 8 | 8 | 8 | 8 | 8 |
| heshui1 | E | YNR | 7 | 7 | 7 | 7 | 7 | 7 | 7 |
| heshui2 | E | YNR | 6 | 6 | 6 | 6 | 6 | 6 | 6 |
| heshui3 | E | YNR | 6 | 6 | 6 | 6 | 6 | 6 | 6 |
| heshui4 | E | YNR | 9 | 9 | 9 | 9 | 9 | 9 | 9 |
| heshui5 | E | YNR | 7 | 7 | 7 | 7 | 7 | 7 | 7 |
| laoxiongqing3 | E | YNR | 10 | 10 | 10 | 10 | 10 | 10 | 10 |
| laoxiongqing4 | E | YNR | 15 | 15 | 15 | 15 | 15 | 15 | 15 |
| laoxiongqing5 | E | YNR | 12 | 12 | 12 | 12 | 12 | 12 | 12 |
| huojianshan1 | E | YNR | 14 | 14 | 14 | 14 | 14 | 14 | 14 |
| huojianshan2 | E | YNR | 12 | 12 | 12 | 12 | 12 | 12 | 12 |
| huojianshan3 | E | YNR | 9 | 9 | 9 | 9 | 9 | 9 | 9 |
| huojianshan4 | E | YNR | 6 | 6 | 6 | 6 | 6 | 6 | 6 |
| baishaheliangzi1 | E | YNR | 12 | 12 | 12 | 12 | 12 | 12 | 12 |
| baishaheliangzi2 | E | YNR | 7 | 7 | 7 | 7 | 7 | 7 | 7 |
| baishaheliangzi3 | E | YNR | 8 | 8 | 8 | 8 | 8 | 8 | 8 |
| baishaheliangzi4 | E | YNR | 8 | 8 | 8 | 8 | 8 | 8 | 8 |
| baishahe1 | E | YNR | 4 | 4 | 4 | 4 | 4 | 4 | 4 |
| baishahe2 | E | YNR | 3 | 3 | 3 | 3 | 3 | 3 | 3 |
| baishahe3 | E | YNR | 5 | 5 | 5 | 5 | 5 | 5 | 5 |
| baishahe6 | E | YNR | 8 | 8 | 8 | 8 | 8 | 8 | 8 |
| baishahekuangshan2 | E | YNR | 8 | 8 | 8 | 8 | 8 | 8 | 8 |
| baishahekuangshan3 | E | YNR | 13 | 13 | 13 | 13 | 13 | 13 | 13 |
| baishahekuangshan4 | E | YNR | 12 | 12 | 12 | 12 | 12 | 12 | 12 |
| baishahekuangshan5 | E | YNR | 17 | 17 | 17 | 17 | 17 | 17 | 17 |
| bumaoshan1 | E | YNR | 3 | 3 | 3 | 3 | 3 | 3 | 3 |
| bumaoshan2 | E | YNR | 14 | 14 | 14 | 14 | 14 | 14 | 14 |
| bumaoshan3 | E | YNR | 16 | 16 | 16 | 16 | 16 | 16 | 16 |
| bumaoshan4 | E | YNR | 6 | 6 | 6 | 6 | 6 | 6 | 6 |
| bumaoshan5 | E | YNR | 10 | 10 | 10 | 10 | 10 | 10 | 10 |
| bumaoshan6 | E | YNR | 12 | 12 | 12 | 12 | 12 | 12 | 12 |
| bumaoshan7 | E | YNR | 13 | 13 | 13 | 13 | 13 | 13 | 13 |
| chapinglao2 | E | YNR | 5 | 5 | 5 | 5 | 5 | 5 | 5 |
| chapinglao3 | E | YNR | 2 | 2 | 2 | 2 | 2 | 2 | 2 |
| chapinglao4 | E | YNR | 8 | 8 | 8 | 8 | 8 | 8 | 8 |
| chapinglao5 | E | YNR | 7 | 7 | 7 | 7 | 7 | 7 | 7 |
| chapinglao6 | E | YNR | 5 | 5 | 5 | 5 | 5 | 5 | 5 |
| chapinglao7 | E | YNR | 9 | 9 | 9 | 9 | 9 | 9 | 9 |
| chaping1 | E | YNR | 8 | 8 | 8 | 8 | 8 | 8 | 8 |
| chaping2 | E | YNR | 8 | 8 | 8 | 8 | 8 | 8 | 8 |
| chaping3 | E | YNR | 14 | 14 | 14 | 14 | 14 | 14 | 14 |
| chaping4 | E | YNR | 8 | 8 | 8 | 8 | 8 | 8 | 8 |
| chaping5 | E | YNR | 10 | 10 | 10 | 10 | 10 | 10 | 10 |
| chaping6 | E | YNR | 11 | 11 | 11 | 11 | 11 | 11 | 11 |
| jishiqing1 | E | YNR | 8 | 8 | 8 | 8 | 8 | 8 | 8 |
| jishiqing2 | E | YNR | 9 | 9 | 9 | 9 | 9 | 9 | 9 |
| jishiqing3 | E | YNR | 12 | 12 | 12 | 12 | 12 | 12 | 12 |
| jishiqing4 | E | YNR | 14 | 14 | 14 | 14 | 14 | 14 | 14 |
| jishiqing5 | E | YNR | 11 | 11 | 11 | 11 | 11 | 11 | 11 |
| jishiqing6 | E | YNR | 11 | 11 | 11 | 11 | 11 | 11 | 11 |
| jishiqing7 | E | YNR | 20 | 20 | 20 | 20 | 20 | 20 | 20 |
| jishiqing8 | E | YNR | 15 | 15 | 15 | 15 | 15 | 15 | 15 |
| choushuitian2 | E | YNR | 8 | 8 | 8 | 8 | 8 | 8 | 8 |
| choushuitian3 | E | YNR | 5 | 5 | 5 | 5 | 5 | 5 | 5 |
| choushuitian4 | E | YNR | 6 | 6 | 6 | 6 | 6 | 6 | 6 |
| guangmazhailiangzi3 | E | YNR | 14 | 14 | 14 | 14 | 14 | 14 | 14 |
| guangmazhailiangzi5 | E | YNR | 9 | 9 | 9 | 9 | 9 | 9 | 9 |
| guangmazhailiangzi6 | E | YNR | 13 | 13 | 13 | 13 | 13 | 13 | 13 |
| huangzhushan2 | E | YNR | 4 | 4 | 4 | 4 | 4 | 4 | 4 |
| huangzhushan4 | E | YNR | 8 | 8 | 8 | 8 | 8 | 8 | 8 |
| huangzhushan6 | E | YNR | 4 | 4 | 4 | 4 | 4 | 4 | 4 |
| huangzhushan7 | E | YNR | 10 | 10 | 10 | 10 | 10 | 10 | 10 |
| jindong1 | E | YNR | 6 | 6 | 6 | 6 | 6 | 6 | 6 |
| jindong2 | E | YNR | 10 | 10 | 10 | 10 | 10 | 10 | 10 |
| laoxianghe27 | E | YNR | 17 | 17 | 17 | 17 | 17 | 17 | 17 |
| laoxianghe28 | E | YNR | 11 | 11 | 11 | 11 | 11 | 11 | 11 |
| laoxianghe29 | E | YNR | 12 | 12 | 12 | 12 | 12 | 12 | 12 |
| laoxianghe30 | E | YNR | 16 | 16 | 16 | 16 | 16 | 16 | 16 |
| laoxianghe31 | E | YNR | 9 | 9 | 9 | 9 | 9 | 9 | 9 |
| laoxianghejia1 | E | YNR | 3 | 3 | 3 | 3 | 3 | 3 | 3 |
| maocaoshan2 | E | YNR | 10 | 10 | 10 | 10 | 10 | 10 | 10 |
| maocaoshan33 | E | YNR | 9 | 9 | 9 | 9 | 9 | 9 | 9 |
| maocaoshan34 | E | YNR | 10 | 10 | 10 | 9 | 9 | 10 | 10 |
| maocaoshan35 | E | YNR | 6 | 6 | 6 | 6 | 6 | 6 | 6 |
| maocaoshan39 | E | YNR | 8 | 8 | 8 | 8 | 8 | 8 | 8 |
| maocaoshan40 | E | YNR | 17 | 17 | 17 | 17 | 17 | 17 | 17 |
| maocaoshan43 | E | YNR | 11 | 11 | 11 | 11 | 11 | 11 | 11 |
| mengban38 | E | YNR | 3 | 4 | 4.44 | 5 | 5.56 | 3 | 3.33 |
| mengban41 | E | YNR | 10 | 10 | 10 | 10 | 10 | 10 | 10 |
| mengban42 | E | YNR | 13 | 13 | 13 | 13 | 13 | 13 | 13 |
| mengban44 | E | YNR | 6 | 6 | 6 | 6 | 6 | 6 | 6 |
| mengban46 | E | YNR | 18 | 18 | 18 | 18 | 18 | 18 | 18 |

1. W: the west bank of the Lan Cang River; E: the east bank of the Lan Cang River.

2. NNR: Nabanhe Basin National Nature Reserve; BNR: Bulong Prefectural Nature Reserve; YNR: Yiwu Prefectural Nature Reserve; XNR: Xishuangbanna National Nature Reserve; MG: ManGao sub-reserve; MY: MengYang sub-reserve; MLu: MengLun sub-reserve; MLa: MengLa sub-reserve; SY: ShangYong sub-reserve; SFF: State Forest Farm.
